# USP22 drives tumor immune evasion and checkpoint blockade resistance through EZH2-mediated epigenetic silencing of MHC-I

**DOI:** 10.1172/JCI193162

**Published:** 2025-11-18

**Authors:** Kun Liu, Radhika Iyer, Yi Li, Jun Zhu, Zhaomeng Cai, Juncheng Wei, Yang Cheng, Amy Y. Tang, Hai Wang, Qiong Gao, Nikita Lavanya Mani, Noah Marx, Beixue Gao, D. Martin Watterson, Seema A. Khan, William J. Gradishar, Huiping Liu, Deyu Fang

**Affiliations:** 1 Department of Pathology, Robert H. Lurie Comprehensive Cancer Center and Center for Human Immunology, Northwestern University Feinberg School of Medicine, Chicago, Illinois, USA.; 2 Department of Oncology, 920th Hospital of Joint Logistics Support Force, Kunming, China.; 3 Center of Metabolic Disease Research, Department of Cardiovascular Sciences, Temple University Lewis Katz School of Medicine, Philadelphia, Pennsylvania, USA.; 4 Department of Pharmacology,; 5 Division of Breast Surgery, Robert H. Lurie Comprehensive Cancer Center,; 6 Division of Hematology/Oncology, Department of Medicine, and; 7 Department of Medicine, Division of Hematology and Oncology, Northwestern University Feinberg School of Medicine, Chicago, Illinois, USA.

**Keywords:** Clinical Research, Immunology, Oncology, Immunotherapy, MHC class 1, Ubiquitin-proteosome system

## Abstract

While immune checkpoint blockade (ICB) therapy has revolutionized the antitumor therapeutic landscape, it remains successful in only a small subset of patients with cancer. Poor or loss of MHC-I expression has been implicated as a common mechanism of ICB resistance. Yet, the molecular mechanisms underlying impaired MHC-I remain to be fully elucidated. Herein, we identified USP22 as a critical factor responsible for ICB resistance through suppressing MHC-I–mediated neoantigen presentation to CD8^+^ T cells. Both genetic and pharmacologic USP22 inhibition increased immunogenicity and overcame anti–PD-1 immunotherapeutic resistance. At the molecular level, USP22 functions as a deubiquitinase for the methyltransferase EZH2, leading to transcriptional silencing of MHC-I gene expression. Targeted *Usp22* inhibition resulted in increased tumoral MHC-I expression and consequently enhanced CD8^+^ T cell killing, which was largely abrogated by *Ezh2* reconstitution. Multiplexed immunofluorescence staining detected a strong reverse correlation between USP22 expression and both β2M expression and CD8^+^ T lymphocyte infiltration in solid tumors. Importantly, USP22 upregulation was associated with ICB immunotherapeutic resistance in patients with lung cancer. Collectively, this study highlights the role of USP22 as a diagnostic biomarker for ICB resistance and provides a potential therapeutic avenue to overcome the current ICB resistance through inhibition of USP22.

## Abstract 2

USP22 is a novel target to overcome the current antitumor immunotherapeutic resistance and a better biomarker for therapeutic guidance.

## Introduction

Immune checkpoint blockade (ICB) therapy works by disrupting inhibitory signals thereby preventing T cell activation and has shown remarkable success in cancer treatment (1). However, the success rate of ICB therapy remains limited to a small fraction of patients (2). The efficacy of ICB therapy relies on cytotoxic CD8^+^ T cell (CTL) recognition of neoantigens presented on major histocompatibility complexes (MHC) class I, which comprises of a heavy-chain and β-2-microglobulin (*B2m*) (3). An important mechanism that cancer cells can develop to escape antigen presentation is the downregulation or absence of MHC class I expression. This weak MHC class I expression leads to a lack of antigen presentation to recruit and activate CD8^+^ cytotoxic T lymphocytes and could explain the limited efficiency of ICB therapy (4). Consequently, aberrant expression of key components within MHC-I antigen processing and presentation are frequently observed across various human cancers, posing a significant barrier to ICB effectiveness in treating many, if not all, human solid tumors (1, 3). However, the molecular mechanisms underlying the downregulation of tumoral MHC-I expression remain largely unknown.

The reduction or loss of MHC-I expression in cancers can occur not only through genomic mutations but also through nongenomic mechanisms that leverage epigenetic and transcriptional silencing of the MHC locus and/or antigen-processing machinery. Multiple regulators such as NOD-like receptor (NLR) family, caspase recruitment domain-containing 5 (NLRC5), NF-κB and IFN regulatory factor 1 (IRF-1) promote MHC-I gene exposure to cytokines such as TNF-α and IFN-γ (5). The Enhancer of Zeste Homolog 2 (EZH2), a catalytic component of Polycomb Repressive Complex 2 (PRC2), which, in turn, is involved in regulating chromatin organization (6), has been identified as a potential therapeutic target for multiple cancers due to its frequent overexpression and role in tumor progression (7). Recent studies have shown that EZH2 contributes to tumor immune evasion by trimethylation of lysine 27 of histone H3 (H3K27me3) on the *B2m* promoter in cancer cells (8). Indeed, EZH2 is found to be overexpressed in various cancers with poor neoantigen presentation (8).

In this study, we identified *USP22*, an oncogene involved in promoting cancer cell growth and tumor immune evasion (9), as a negative regulator of MHC-I expression across a variety of human and mouse cancer cell lines. Targeted CRISPR-mediated deletion of *Usp22* resulted in enhanced tumor cell antigen presentation and tumor-specific CD8^+^ cell immunity. At the molecular level, USP22 associates with and deubiquitinates EZH2, thereby protecting it from proteasomal degradation. Analysis of human cancer tissues revealed a positive correlation of USP22 with EZH2, both of which were negatively correlated with MHC-I expression and intratumoral CD8^+^ T cell infiltration. Importantly, increased USP22 expression is associated with ICB immunotherapeutic resistance and pharmacological USP22 inhibition overcomes ICB resistance.

## Results

### USP22 is a negative regulator of MHC-I–mediated neoantigen presentation in tumor cells.

**Figure 1:**
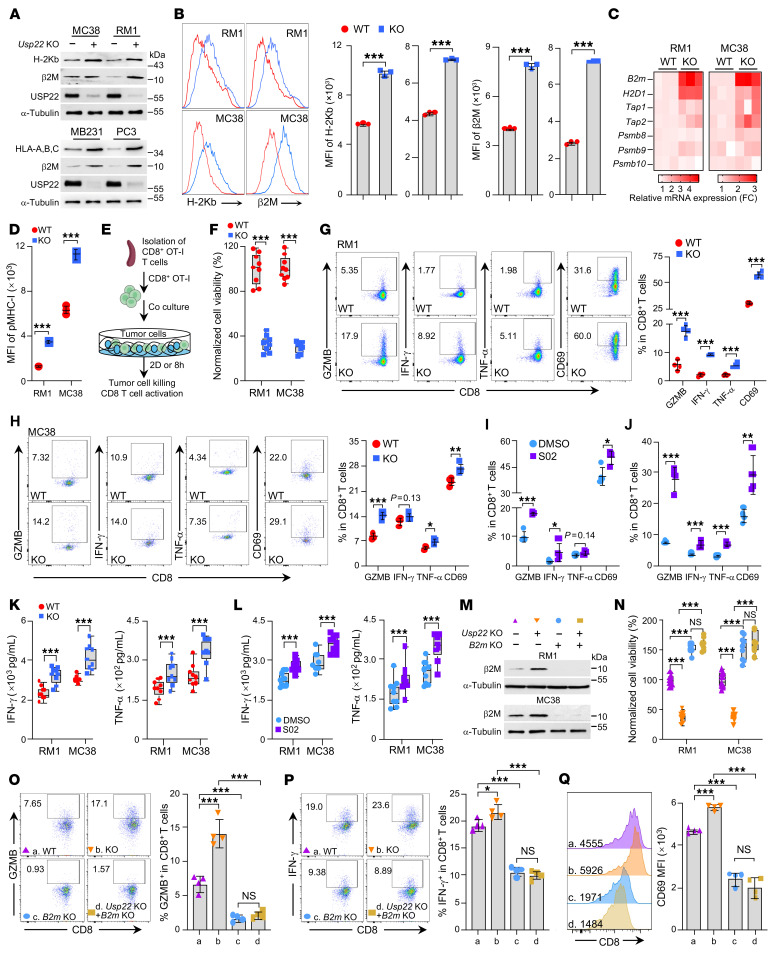
*Usp22* inhibition enhances MHC-I expression. Indicated cells were transfected with control (WT) or *Usp22*-specific guide RNAs (*Usp22* KO). (**A**) Immunoblot analysis of MHC-I proteins in WT and *Usp22* KO tumor cells. (**B**) Cell surface expression of H-2Kb and b2M were determined in WT and *Usp22* KO cells. (**C**) Heatmap summarizing for the mRNA expression of genes involved in antigen presentation in WT and KO tumor cells. (**D**) Cell surface levels of OVA peptide SIINFEKL (pMHC-I) were determined in WT and *Usp22* KO MC38/OVA or RM1/OVA cells. (**E**) Schematic illustration of an in vitro cytotoxicity assay. (**F**) The viability of WT and *Usp22* KO MC38/OVA or RM1/OVA after cocultured with OT-I CD8^+^ T cells. (**G** and **H**) OT-I CD8^+^ T cell activation after cocultured with WT and *Usp22* KO RM1/OVA or MC38/OVA cells were determined. (**I** and **J**) RM1/OVA (**I**) or MC38/OVA (**J**) cells were pretreated with or without 20 μM USP22i-S02 for 48 hours and then cocultured with OT-I CD8^+^ T cells. OT-I CD8^+^ T cell activation was determined as in **I** and **J**. (**K**) WT and *Usp22* KO RM1/OVA or MC38/OVA cells were cocultured with OT-I CD8 T cells. The concentrations of IFN-γ or TNF-α in the supernatant were determined by ELISA (*N* = 9). (**L**) RM1/OVA or MC38/OVA cells were pretreated with USPi-S02 as in **I** and then cocultured with OT-I cells. The concentrations of IFN-γ and TNF-α in the supernatant were determined by ELISA (*N* = 9). (**M**) *B2m* was deleted by CRISPR in WT and *Usp22* KO MC38/OVA and RM1/OVA cells. (**N**–**Q**) The effect of *B2m* deletion on CD8-mediated killing of tumor cells (**N**) and OT-I CD8^+^ T cell activation was determined as in **P** and **Q**. Statistics were calculated by unpaired 2-tailed *t* test (**B**, **D**, and **F**–**L**) or 1-way ANOVA followed by Tukey’s test (**N**–**Q**). *P < 0.05, **P < 0.01, and ***P < 0.001.

We and others have recently revealed that inhibition of *USP22* plays a role in both oncotargeting and boosting the antitumor immune response (9, 10). To further explore the role of tumoral USP22 in evading immune surveillance, we analyzed the potential effect of *Usp22* inhibition on neoantigen presentation. CRISPR-mediated deletion of *Usp22* in mouse prostate cancer RM1, colon cancer MC38, and breast cancer 4T1 cells resulted in a substantial elevation in the expression of both H-2Kb/d and β2M, 2 subunits of the MHC-I complex (Figure 1A and Supplemental Figure 1A; supplemental material available online with this article; https://doi.org/10.1172/JCI193162DS1). Flow cytometry analysis further confirmed the increase in cell surface expression of H-2Kb/d and β2M in *Usp22*-null tumor cells (Figure 1B). *Usp22* inhibition resulted in a similar increase in HLA-ABC and β2M expression in both human prostate cancer PC3 and triple negative breast cancer (TNBC) MDA-MB-231 cells (Figure 1A). Further RT-PCR analysis detected an elevation of *H2D1* (encode H-2Kb) and *B2m* (encode β2M) mRNA levels by *Usp22* ablation (Figure 1C and Supplemental Figure 1B). Consistently, pharmacological USP22 inhibition by USP22i-S02, a small-molecule inhibitor developed by our group (11), boosted both H-2Kb and β2M expression (Supplemental Figure 1C). Conversely, *Usp22* over-expression resulted in a modest but statistically significant decrease in MHC-I expression (Supplemental Figure 1D). In addition, MHC-II was undetectable in RM1 cells, and only very low levels were observed in MC38 cells. In both cell lines, neither *Usp22* knockout nor overexpression altered MHC-II expression (Supplemental Figure 1E). It is well established that IFN-γ is a critical inducer for MHC-I expression (12). To investigate the role of USP22 in IFN-γ–induced tumoral MHC-I expression, we analyzed its effects under these conditions. As expected, IFN-γ treatment substantially increased the expression of β2M and H2Kb/d in WT cancer cells. However, in *Usp22* knockout tumor cells, IFN-γ treatment failed to further enhance MHC-I expression (Supplemental Figure 1, F–H). These results indicate that USP22 is a negative regulator of MHC-I expression possibly at the transcriptional level, implying that USP22 achieves its tumor immune evasive functions through, at least in part, suppression of MHC-I expression.

MHC-I–mediated antigen presentation is crucial for activating CD8^+^ T cells (3). To determine the impact of USP22-mediated MHC-I downregulation on CD8^+^ T cell immunity, we generated WT and *Usp22-*deficient RM1 and MC38 cells stably expressing OVA (13–15). As expected, *Usp22* ablation cells exhibited a higher level of the OVA peptide (SIINFEKL)-bound MHC-I complex (pMHC-I) (Figure 1D and Supplemental Figure 1I). Reconstitution with *Usp22*, but not the catalytically inactive *Usp22* (*Usp22* C185A) mutant, in *Usp22*-deficient cells completely reversed the increased MHC-I levels (Supplemental Figure 1J), suggesting that the deubiquitylase activity of USP22 is required in downregulating MHC-I–mediated antigen presentation. Coculture of CD8^+^ OT-I T cells with either *Usp22* KO MC38 or RM1 cancer cells with stable OVA expression enhanced CD8^+^ T cell activation, indicated by elevated CD69 expression and tumor cell killing (Figure 1, E–H). Furthermore, intracellular staining confirmed the increased production of granzyme B, IFN-γ, and TNF-α by CD8^+^ OT-I T cells (Figure 1, G and H). Consistent with these findings, pharmacological inhibition of *Usp22* in tumor cells by treatment with USP22i-S02 enhanced the activation of CD8^+^ OT-I T cells. (Figure 1, I and J, and Supplemental Figure 1K). Moreover, ELISA analysis detected a substantial increase in both IFN-γ and TNF-α secretion in the supernatant when CD8^+^ OT-I T cells were cocultured with *Usp22* KO or pharmacological inhibition tumor cells (Figure 1, K and L). Collectively, these results indicate that USP22 downregulates MHC-I to suppress CD8^+^ T cell antitumor immunity. To support this conclusion, targeted deletion of *B2m*, an essential component of MHC-I, while having no effect on cell proliferation (Figure 1M and Supplemental Figure 2, A and B) as reported (16), totally abolished the increased CD8^+^ OT-I T cell-mediated killing of *Usp22*-deficient tumor cells (Figure 1N), as well as the activation and increased secretion of IFN-γ and TNF-α (Figure 1, O–Q, and Supplemental Figure 2, C–F). These results indicate that tumoral *Usp22* inhibition-mediated increase in CD8^+^ T cell antitumor immunity is dependent on MHC-I upregulation.

### Tumoral Usp22 inhibition enhances antitumor immune response through upregulating MHC-I–mediated neoantigen presentation to CD8^+^ T cells.

**Figure 2:**
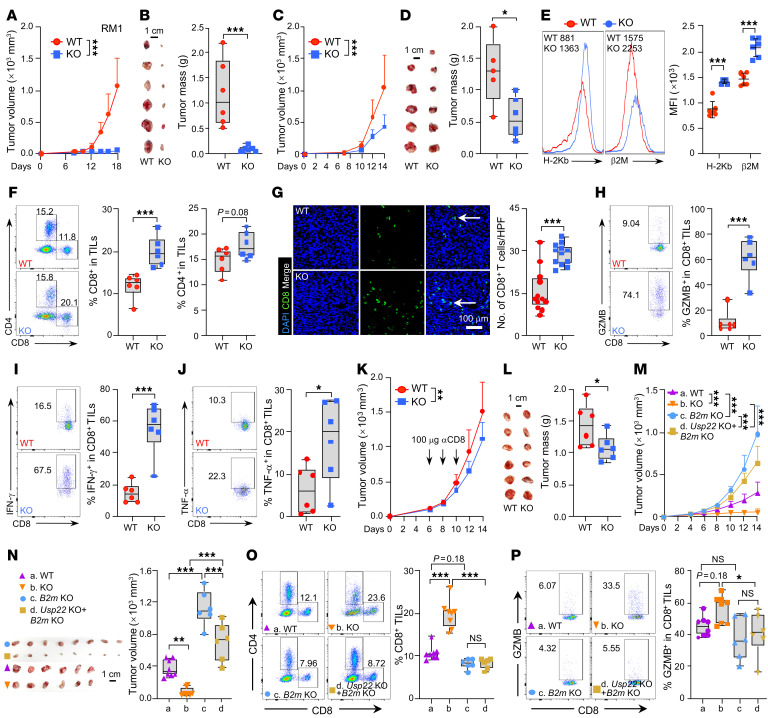
The absence of *Usp22* dampens tumor growth by enhancing tumoral infiltrating CD8^+^ T cells. (**A** and **B**) Effect of *Usp22* depletion on tumorigenesis of RM1 cells in C57BL/6J mice. Tumor volume (**A**), endpoint tumor images and weight (**B**) are shown. Scale bar: 1 cm. (**C** and **D**) Effect of *Usp22* depletion on tumorigenesis of RM1 cells in immunocompromised RAG1 knockout mice were determined as in **A** and **B**, Scale bars: 1 cm. (**E**) Flow cytometric analysis of the expression of H-2Kb or b2M on tumoral cells in **A**. (**F** and **G**) Tumoral infiltrating CD4^+^ and CD8^+^ T cells on the total CD45^+^ cells in tumors shown in **A** were analyzed by flow cytometry (**F**) or immunofluorescence staining (**G**). Scale bar: 100 μm. HPF, high powered field. (**H**–**J**) The production of granzyme B^+^ (**H**), IFN-γ^+^ (**I**), or TNF-α^+^ (**J**) by CD8^+^ in **F**. (**K** and **L**) Tumor-bearing mice were treated with CD8 depleting antibodies (100 mg) on day 6, 9, and 12. Tumor volume (**K**), endpoint tumor images, and weight (**L**) were recorded. Scale bar: 1 cm. (**M** and **N**) WT, *Usp22* KO, *B2m* KO or double KO (dKO) RM1 cells were subcutaneously injected into C57BL/6J mice, tumor volume (**M**), endpoint tumor images, and weight (**N**) are shown. Scale bar: 1 cm. (**O** and **P**) Tumoral infiltrating CD8^+^ T cells (**O**) or their production of GZMB (**P**) were analyzed. Statistics were calculated by unpaired 2-tailed *t* test (**B**, **D**, **E**–**J**, and **L**) or 1-way ANOVA followed by Tukey’s test (**N**–**P**). Two-way ANOVA with multiple comparisons (**A**, **C**, **K**, and **M**). *P < 0.05, **P < 0.01, and ***P < 0.001.

Next, we investigated the functional consequences of *Usp22*-mediated MHC-I downregulation in the antitumor immune response. Importantly, CRISPR deletion of *Usp22* led to a nearly complete rejection of syngeneic RM1 prostate cancer in immunocompetent C57BL/6J mice (Figure 2, A and B). In contrast, *Usp22* suppression only resulted in a modest reduction in RM1 tumor growth both in RAG1 KO mice (Figure 2, C and D), as well as in vitro (Supplemental Figure 3A). These results support our previous conclusion that while *Usp22* is an oncogene and promotes tumor growth (17–20), the antitumor immune response plays a much greater role in tumor rejection. To support this hypothesis, we confirmed the increase in MHC-I expression in *Usp22*-null tumors (Figure 2E), along with a marked increase in intratumoral CD8^+^ T cell infiltration as analyzed by both flow cytometry (Figure 2F) and IHC staining (Figure 2G). Intracellular staining detected a marked increase in CD8^+^ T cell production of granzyme B, IFN-γ and TNF-α (Figure 2, H–J). Therefore, tumoral *Usp22* inhibition enhances CD8^+^ T cell antitumor immunity. To support this, further depletion of CD8^+^ T cells using CD8-depleting antibody (αCD8) largely diminished the increased rejection of *Usp22-*deficient tumors (Figure 2, K and L).

In addition to RM1 prostate cancer, we confirmed that *Usp22* inhibition impeded the growth of both orthotopic 4T1 TNBC (Supplemental Figure 3, B and C) and MC38 syngeneic tumors (Supplemental Figure 4, A and B). Additionally, the tumor suppressive efficacy of *Usp22* inhibition was modest when MC38 tumor cells were implanted into RAG1-KO mice (Supplemental Figure 4, C and D). The increased tumor suppression by *Usp22* deletion is associated with increased tumor cell surface H-2Kb and β2M expression (Supplemental Figure 3D and Supplemental Figure 4E), the elevated tumoral-infiltrating CD8^+^ T cells (Supplemental Figure 3, E and F, and Supplemental Figure 4, F and G) and their production granzyme B, IFN-γ, and TNF-α (Supplemental Figure 3, G–I, and Supplemental Figure 4, H–J). Further depletion of CD8^+^ T cells using CD8-depleting antibody (αCD8) largely diminished the increased regression of *Usp22*-deficient tumors (Supplemental Figure 3, J and K, and Supplemental Figure 4, K and L). These results indicate that *Usp22* promotes the evasion of CD8^+^ T cell antitumor immunity across a broad spectrum of cancer types through, at least in part, downregulating MHC-I expression. To further support this conclusion, we found that silencing of *B2m* expression completely abolished the improved antitumor immune response associated with *Usp22* inhibition (Figure 2, M–P, and Supplemental Figure 4, M–P).

Further, we observed that *Usp22* targeted inhibition synergized with anti–PD-1 treatment leading to a complete rejection of orthotopic 4T1 TNBC, MC38 colon cancer and RM1 prostate cancer (Supplemental Figure 5, A–F). Flow cytometry analysis of intratumoral immune cells confirmed the synergistic effects of tumoral *Usp22* inhibition in boosting antitumor immunity with increased CD8^+^ T cell infiltration and production of granzyme B and IFN-γ (Supplemental Figure 5, G–I). Similar to the CRISPR-targeted *Usp22* inhibition, USP22i-S02 treatment synergized with anti–PD-1 resulted in a nearly complete inhibition of both RM1 and MC38 tumor growth with increased CD8 antitumor immunity (Supplemental Figure 6). These results indicate that tumoral *Usp22* inhibition sensitizes ICB antitumor immunotherapy.

### Usp22 inhibits MCH-I expression through upregulating EZH2 in cancer cells.

**Figure 3:**
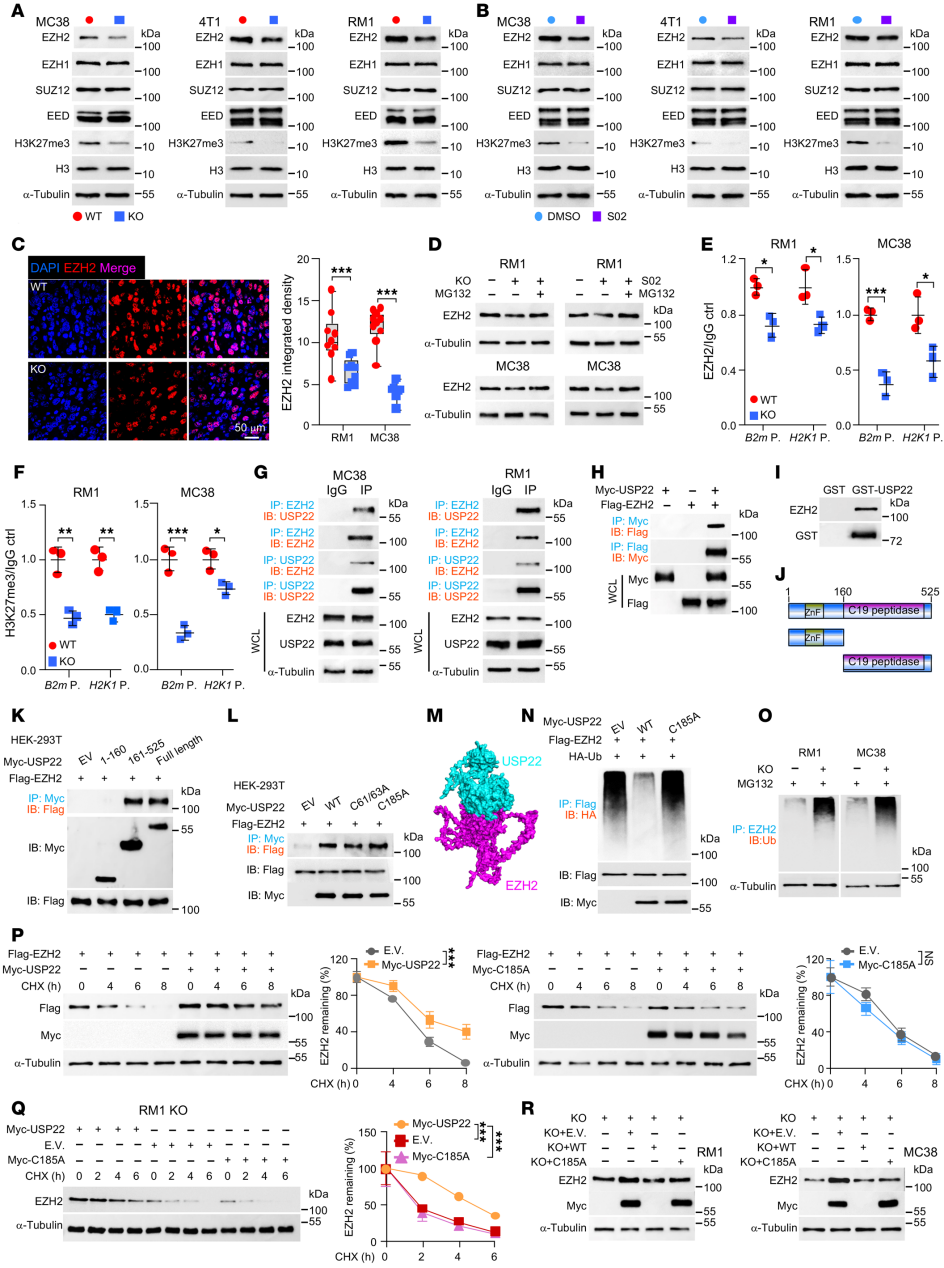
USP22 is an EZH2-specific deubiquitinase. (**A** and **B**) Immunoblot analysis of indicated protein levels in WT and KO tumor cells (**A**) or in tumor cells treated with 20 μM USP22i-S02 (**B**). (**C**) Immunofluorescence staining and quantification of EZH2 in WT and KO RM1 tumors. Scale bars: 50 μm. (**D**) Immunoblot analysis of indicated protein levels in WT and KO cells treated with or without MG132 (10 μM, 8 hours). (**E** and **F**) Ch-IP and qRT-PCR analysis for EZH2 (**E**), H3K27me3 (**F**) enrichment in *B2m* or *H-2K1* genes promoter in WT and KO cells. (**G**) Analysis of USP22 interaction with PCR2 complex proteins by Co-IP and immunoblot. WCL, whole cell lysates. (**H**) Analysis of USP22 interaction with EZH2 in transiently transfected HEK-293T cells. (**I**) Recombinant GST/GST-USP22 were purified from bacteria and incubated with 4T1 cell lysate overnight. The binding proteins were analyzed by immunoblot. (**J**) Schematic illustration of USP22 and its truncated mutants. (**K** and **L**) Analysis of EZH2 interaction with USP22 and its mutants in transiently transfected HEK-293T cells. (**M**) Molecular docking analysis of the interaction between USP22 and EZH2. (**N**) EZH2 ubiquitination was determined from HEK293T cells in the presence of transient transfection of Myc-USP22/C185A, HA-ubiquitin. (**O**) Indicated cells were pretreated with 10 μM MG132 for 8 hours, EZH2 ubiquitination was determined. (**P**) HEK-293T cells cotransfected with FLAG-EZH2 and Myc-USP22 or its C185A mutant. After 24 hours’ transfection, cells were treated with 20 mg/mL cycloheximide (CHX) for the indicated time points and indicated protein levels were determined. (**Q**) RM1 KO cells were transfected with *Usp22*/C185A mutant. EZH2 protein stability was determined as in **P**. (**R**) EZH2 protein stability in WT and KO RM1 and MC38 cells were determined as in **P**. Statistics were calculated by unpaired 2-tailed *t* test (**C**, **E**, and **F**) or 2-way ANOVA with multiple comparisons (**P** and **Q**). *P < 0.05, **P < 0.01, and ***P < 0.001.

As a ubiquitin-specific peptidase, *Usp22* often achieves pathological functions through protecting its substrates from ubiquitination-mediated protein degradation (11, 17, 18). We then analyzed the protein expression levels of previously identified MHC-I regulators, including PRC2 proteins (EZH1, EZH2, SUZ12, and EED) (8), NLRC5 (21), METTL3 (22), and METTL14 (23), in *Usp22*-null versus control cells to identify potential substrates of USP22. Interestingly, among these MHC-I regulators, *Usp22* depletion resulted in a distinct reduction in the protein expression of EZH2, a core component of the PRC2 complex. In contrast, the expression of other PCR2 complex proteins, including EED, EZH1, and SUZ12 were unaffected (Figure 3A). As a methyltransferase, EZH2 has been shown to methylate histone H3 lysine 27 (H3K27me3), which is critical for tumorigenesis in part through the silencing of MHC-I gene transcription (24, 25). Along with EZH2 downregulation, the trimethylation level of histone H3 lysine 27 in *Usp22*-null tumor cells was reduced (Figure 3A). In contrast, *Usp22* depletion did not alter the expression of NLRC5, METTL3, and METTL14 (Supplemental Figure 7A). Similar to that of *Usp22-*targeted deletion, treatment of tumor cells with USP22i-S02 led to a substantial reduction in EZH2, but not EED, EZH1, and SUZ12 (Figure 3B). Furthermore, IHC staining confirmed the reduced EZH2 expression in *Usp22*-null tumor cells (Figure 3C and Supplemental Figure 7B). Intriguingly, neither genetic nor pharmacological *Usp22* inhibition had any effect on *EZH2* mRNA expression in MC38, 4T1, and RM1 tumor cells (Supplemental Figure 7C), suggesting that *Usp22* regulates EZH2 at the posttranscriptional level. Indeed, treatment with the proteasomal inhibitor MG132, but not the lysosomal inhibitor chloroquine, fully rescued EZH2 protein expression in cancer cells either with *Usp22* deletion or treated with the USP22 small-molecule inhibitor S02 (Figure 3D and Supplemental Figure 7D). These results suggest that USP22 suppresses tumoral MHC-I expression through EZH2 upregulation at the posttranscriptional level.

EZH2 is known to silence MHC-I expression through epigenetic suppression (8, 26). Indeed, both the recruitment of EZH2 to the promoter regions of *B2m* and *H2Kb* and their H3K27me3 modification levels, was decreased in USP22-null tumor cells (Figure 3, E and F). Consistently, both genetic or pharmacologic inhibition of EZH2 enhanced H-2Kb and β2M expression (Supplemental Figure 7, E–H) and reduced H3K27me3 modification levels on *H2K1* and *B2m* promoter region in RM1 and MC38 cancer cells (Supplemental Figure 7I). In contrast, the recruitment of KDM6A, a histone demethylase known to regulate MCH-I expression through inhibiting H3K27me3 at *B2m* and *H2Kb* promoter regions, was unaltered by *Usp22* inhibition (Supplemental Figure 7J). Collectively, our results indicate that USP22 represses antitumor immunity in part through potentiating EZH2-mediated transcriptional downregulation of MHC-I expression.

### USP22 is a de novo EZH2-specific deubiquitinase.

To further delineate underlying molecular mechanisms by which USP22 specifically controls EZH2 protein expression in tumor cells, we first determined whether USP22 interacts with EZH2. Indeed, Western blotting detected USP22 protein in the anti-EZH2 immunoprecipitated from the lysates of RM1 and MC38 cells (Figure 3G). Reciprocally, EZH2 was detected in the anti-USP22 pulldown (Figure 3G). The interaction between USP22 and EZH2 was further confirmed in HEK-293T cells transfected with Myc-tagged USP22 and Flag-tagged EZH2 (Figure 3H). Additionally, EZH2 protein was detected from GST-USP22 pulldown but not GST protein alone (Figure 3I). USP22 protein consists of an N-terminal zinc finger domain followed by a C19 ubiquitin-specific peptidase domain. We then generated USP22-truncated mutants and found that the C-terminus C19 peptidase domain, but not the N-terminal zinc finger–containing region, is sufficient to mediate USP22 interaction with EZH2 (Figure 3, J and K). Consistent with this, mutation of the critical cystines in the zinc finger structure did not affect USP22 interaction with EZH2 (Figure 3L). Molecular docking analysis revealed that the USP22 C-terminal U19 domain mediates its interaction with EZH2 (Figure 3M). Collectively, these results indicate that USP22 is a de novo interacting partner of EZH2 in tumor cells.

A deubiquitinase often inhibits the ubiquitination of its interacting proteins (27). Indeed, ectopic expression of *Usp22* inhibited the ubiquitination of EZH2 (Figure 3N). In contrast, the catalytically inactive *Usp22*-C185A while still interacting with EZH2, did not show any effect on EZH2 ubiquitination (Figure 3, L and N). Conversely, targeted deletion of *Usp22* resulted in enhanced EZH2 ubiquitination both RM1 and MC38 tumor cells (Figure 3O). Collectively, our results indicate that USP22 is a de novo deubiquitinase of the MHC-I suppressor EZH2 in cancer cells.

### EZH2 is responsible for USP22-mediated downregulation of MHC-I.

A deubiquitinase suppresses the ubiquitination of its target proteins to regulate their biological functions through degradation or subcellular distribution. Our results that targeted *Usp22* inhibition decreased EZH2 protein, but not its mRNA expression levels (Figure 3, A–C, and Supplemental Figure 7C), indicate that USP22 upregulates EZH2 through suppressing its ubiquitination-mediated protein degradation. As expected, overexpression of WT *Usp22*, but not *Usp22*-C185A, improved EZH2 stability (Figure 3P). In contrast, *Usp22* deletion promoted EZH2 protein degradation, which was fully rescued by WT *Usp22*, but not *Usp22*-C185A (Figure 3, Q and R).

**Figure 4:**
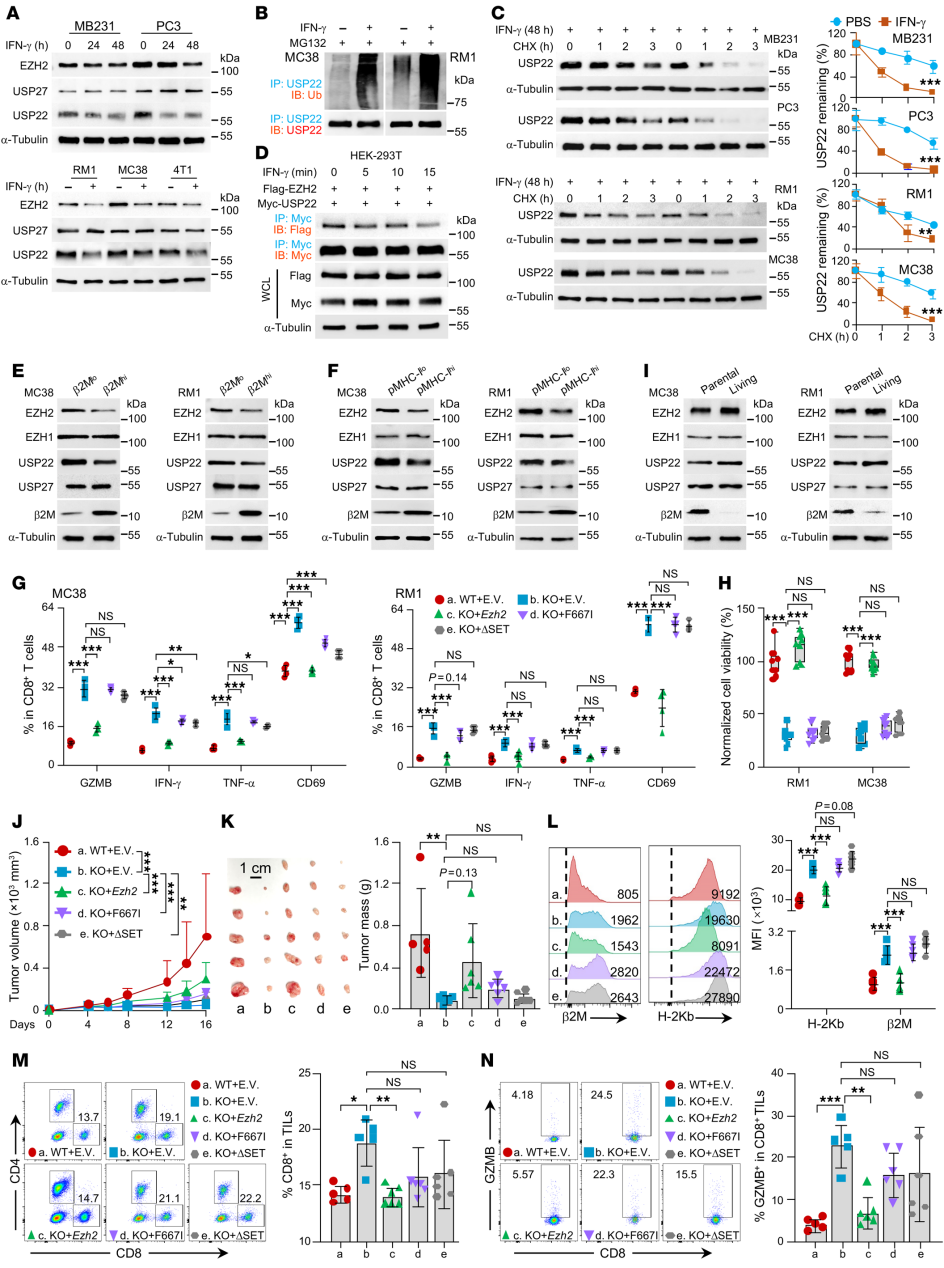
USP22 attenuates antitumor immunity partially through protecting EZH2 from degradation. (**A**–**C**) Indicated cancer cells were treated with 10 ng/mL IFN-γ for indicated time points. The expression of indicated proteins was determined. (**D**) HEK-293T cells were cotransfected with FLAG-EZH2 and Myc-USP22 and then treated with 10 ng/mL IFN-γ for the indicated times. The interaction between USP22 and EZH2 was determined. (**E**) Tumor cells were isolated based on membrane b2M expression. Indicated protein expression was determined. (**F**) MC38/OVA or RM1/OVA were isolated according to cell surface pMHC-I. Indicated protein expression was determined. (**G**) OT-I CD8^+^ T cells were isolated from OT-I mice and cocultured with *Usp22*-deficient RM1/OVA or MC38/OVA cells with or without *Ezh2*, *Ezh2* F667I, or ΔSET mutant reconstitution for 8 hours at the ratio of 1:1 in the presence of CD28 blocking antibodies treatment. Quantification data of flow cytometric analysis of percentages of GZMB^+^, IFN-γ^+^, and TNF-α^+^ producing CD8^+^ T cells are shown. (**H**) Cell viability of indicated cells after coculturing for 48 hours. (**I**) Living tumor cells were collected after coculture with naive OT-I CD8^+^ T cells for 48 hours at a ratio of 1:1 in the presence of CD28 blocking antibody treatment. Indicated protein levels were determined. (**J** and **K**) MC38 cells with *Ezh2*, *Ezh2* F667I, or ΔSET mutant reconstitution in the setting of *Usp22* depletion were inoculated into immunocompetent mice. Tumor volume (**J**) and endpoint mass (**K**) of indicated tumors were recorded. (**L**) The expression of b2M and H-2Kb on indicated tumor cell surface. (**M** and **N**) The frequencies of tumoral-infiltrating CD8^+^ T cells (**M**) or GZMB^+^ producing CD8^+^ T cells (**N**) from indicated MC38 tumors. Statistics were calculated by 1-way ANOVA followed by Tukey’s test (**G**, **H**, and **K**–**N**). Two-way ANOVA with multiple comparisons (**C** and **J**). *P < 0.05, **P < 0.01, and ***P < 0.001.

Unexpectedly, we observed that IFN-γ treatment led to a distinct reduction in USP22 protein levels (Supplemental Figure 1E). Consequently, a marked decrease in EZH2 protein was also detected in tumor cells following IFN-γ treatment (Figure 4A). Notably, IFN-γ treatment did not affect *Usp22* or *Ezh2* mRNA levels (Supplemental Figure 8A), suggesting that IFN-γ regulates USP22 and its substrate EZH2 at a posttranslational level. Supporting this, treatment with the proteasome inhibitor MG132 fully protected USP22 protein from IFN-γ–induced downregulation, whereas the lysosomal inhibitor chloroquine failed to rescue USP22 and EZH2 expression (Supplemental Figure 8B). Further analysis revealed that IFN-γ treatment promotes USP22 ubiquitination and degradation (Figure 4, B and C). Interestingly, IFN-γ also disrupted the interaction between USP22 and EZH2 after treatment with IFN-γ for only 15 minutes even before USP22 and EZH2 degraded (Figure 4D). These findings suggest that IFN-γ induces MHC-I expression by promoting USP22 ubiquitination-mediated degradation. Consistent with this, genetic inhibition of IFN-γ receptor 1 (*IFNGR1*) completely abolished IFN-γ–induced USP22 downregulation (Supplemental Figure 8C).

Our data thus far demonstrate that USP22 protects EZH2, a known negative regulator of MHC-I expression (8), from ubiquitination-mediated proteasomal degradation, suggesting that USP22 promotes tumor evasion of CD8^+^ T cell antitumor immunity through potentiating EZH2-mediated MHC-I downregulation. Indeed, reconstitution of *Ezh2*, but not its inactive methyltransferase mutant either by F667I mutation, or by deletion of the catalytic SET domain, fully reversed MHC-I expression levels in *Usp22*-null tumor cells (Supplemental Figure 8, D–F). Consistent with our data that USP22 represses MHC-I expression through EZH2-mediated H3K27me3 at *B2m* and *H2Kb* promoters (Figure 3F), further real-time-PCR analysis confirmed that the USP22-EZH2 axis controls MHC-I expression at the mRNA level (Supplemental Figure 8G). Consistently, isolated RM1 and MC38 cells with lower β2M and pMHC-I levels exhibited higher levels of USP22 and EZH2 protein, and vice versa (Figure 4, E and F). In contrast, neither USP27 nor EZH1 expression was associated with β2M or pMHC-I levels (Figure 4, E and F). Therefore, when cocultured with OT-I CD8^+^ T cells, expression of EZH2, but not its catalytically inactive mutants in *Usp22*-null tumor cells, totally diminished the increase in OT-I CD8^+^ T cell activation, including the production of granzyme B, IFN-γ, and TNF-α, cell surface expression of CD69, and OT-I mediated cytotoxicity (Figure 4, G and H, and Supplemental Figure 8, H and I). Conversely, further analysis of surviving tumor cells 48 hours after cocultivation with OT-I CD8^+^ T cells showed a higher USP22 and EZH2, but not EZH1 and USP27 expression (Figure 4I), implying that the increased USP22 and EZH2 expression is involved in tumor immune evasion.

Consistent with our in vitro studies, stable reconstitution of *Ezh2*, but not *Ezh2* F667I or ΔSET mutant, largely abrogated the tumor-suppressive effects by the targeted *Usp22* inhibition (Figure 4, J and K, and Supplemental Figure 9, A and B). Cell surface staining of MHC-I expression on tumor cells indicated that overexpression of *Ezh2*, but not *Ezh2* F667I or ΔSET mutant, in *Usp22*-null cells impaired MHC-I expression (Figure 4L and Supplemental Figure 9C). Consequently, the increased CD8^+^ T cell infiltration as well as GZMB production, were largely reversed by the reconstitution of *Ezh2*, but not its *Ezh2* F667I or ΔSET mutants (Figure 4, M and N, and Supplemental Figure 9, D and E). Thus, USP22 drives immune evasion largely in an EZH2-dependent manner.

### Clinical relevance of USP22-EZH2-β2M signaling in tumorigenesis.

**Figure 5:**
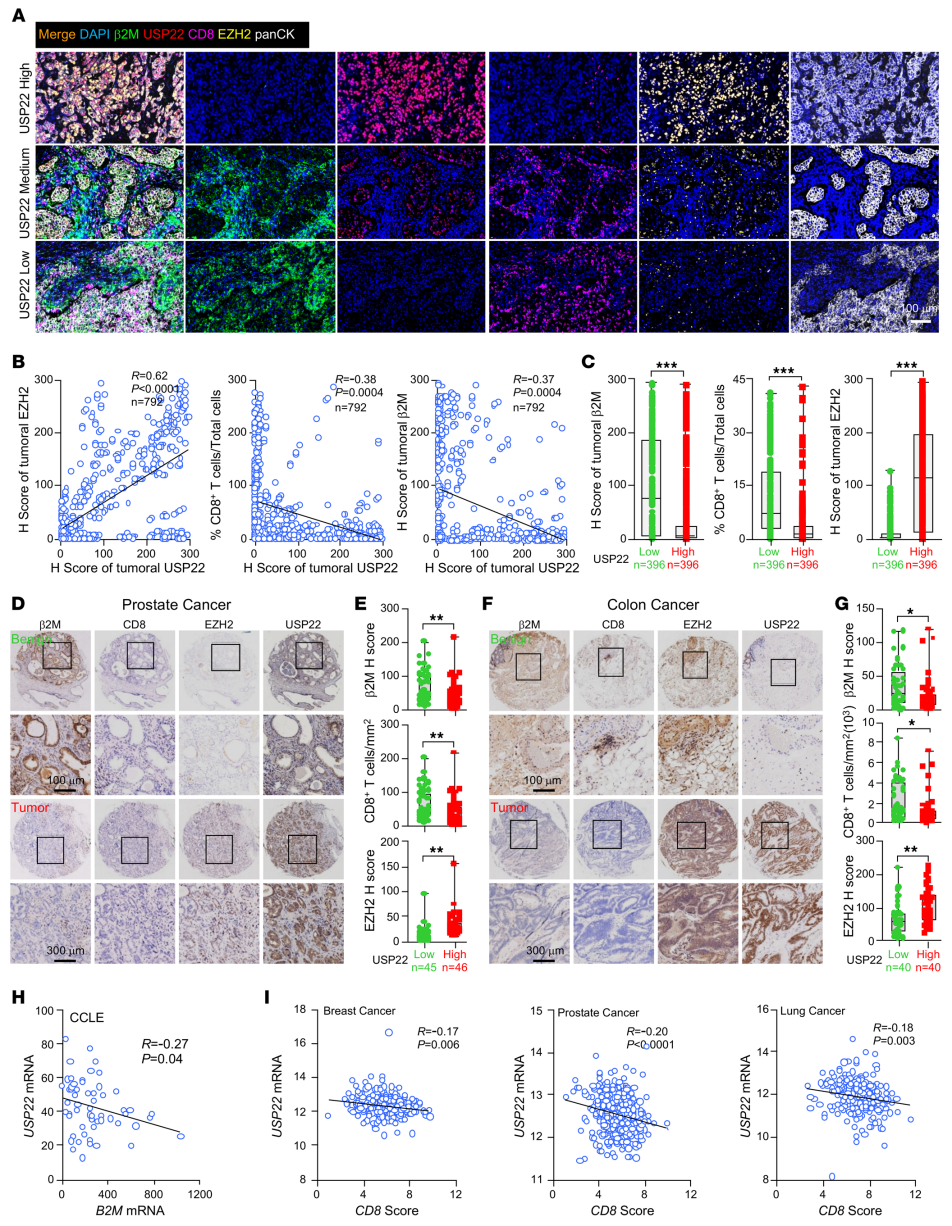
Analysis of USP22/EZH2/b2M expression in multiple types of cancers. (**A**) Representative images of multiplexed immunofluorescence staining of USP22/EZH2/b2M/CD8 in breast cancer tissues with different USP22 intensity. Scale bar: 100 μm. H-score, histochemical scoring assessment. (**B**) The correlation between USP22 with b2M, USP22 with CD8, and USP22 with EZH2. (**C**) Quantification of tumoral-infiltrated CD8^+^ T cells, EZH2, or b2M intensity in breast cancer tissues with different USP22 intensity. Patients were classified into the USP22 intensity low or high group. The median value was used as cutoff. (**D**) Immunohistochemical staining of USP22/EZH2/b2M/CD8 in a prostate cancer tissue microarray. Scale bar: 200 μm. (**E**) The proportion of tumor-infiltrating CD8^+^ T cells or b2M and EZH2 intensity in different intensity cohorts. Patients were classified into the USP22 intensity low or high group. The median value was used as cutoff. (**F**) Immunohistochemical staining of USP22/EZH2/b2M/CD8 in colorectal tissue microarray containing 80 paired benign or colorectal cancer tissues. Scale bar: 200 μm. (**G**) The proportion of tumoral-infiltrated CD8^+^ T cells or b2M and EZH2 intensity in low or high USP22 intensity cohorts. Patients were classified into the USP22 intensity low or high group. The median value was used as cutoff. (**H**) Correlations between the mRNA levels of *USP22* and *B2M* in breast cancer cell lines from Cancer Cell Line Encyclopedia (CCLE). (**I**) Correlations between the mRNA expression of *USP22* and CD8 infiltration score in prostate or colorectal cancer from TCGA database. Statistics were calculated by unpaired 2-tailed *t* test (**C**, **E**, and **G**), 2-tailed Pearson correlation test (**B**, **H**, and **I**). *P < 0.05, **P < 0.01, and ***P < 0.001.

We next determined whether the USP22-EZH2-β2M pathway was associated with CD8^+^ T cell infiltration into tumors. A human breast cancer tissue microarray was used for multiplex immunofluorescence staining, as reported (28). Consistent with our findings that USP22 protects EZH2 from ubiquitination-mediated degradation, both USP22 and EZH2 proteins were highly expressed and positively correlated in tumor tissues compared with adjacent normal tissues (Figure 5, A and B, and Supplemental Figure 10, A and B). Importantly, substantially lower β2M expression levels along with a markedly reduced intratumoral CD8^+^ T cell infiltration were detected in USP22 high compared with low tumor groups (Figure 5, A and C). Therefore, a negative correlation of tumoral USP22 with tumoral β2M, and with CD8^+^ T cell infiltration was detected (Figure 5B). These results support our conclusion that high USP22 expression contributes to tumor immune evasion through potentiating EZH2-mediated HLA-I downregulation.

Consistent with our observations in human breast cancers, IHC staining of EZH2, USP22, β2M, and CD8^+^ in serial tissue sections in human prostate and colon cancer tissue microarrays confirmed the increased expression of USP22 and EZH2 in tumors compared with benign tissues (Figure 5, D–G, Supplemental Figure 11, A and B, and Supplemental Figure 12, A and B). Both β2M and CD8 intratumoral infiltration were markedly lower in the USP22-high compared with USP22-low tumors (Figure 5, D–G). We further unbiasedly analyzed the *USP22* and *B2M* transcripts in breast cancer cell lines listed in Cancer Cell Line Encyclopedia (CCLE). The results demonstrated an inverse correlation between *USP22* and *B2M* expression (Figure 5H). TCGA analysis showed a similar negative association of USP22 expression with CD8 scores in breast, prostate, and lung cancer (Figure 5I). Collectively, these results indicate that the USP22-EZH2-β2M pathway is a common molecular mechanism for poor MHC-I expression in a broad spectrum of human cancers.

### Elevated USP22 expression is linked to poor ICB response.

**Figure 6:**
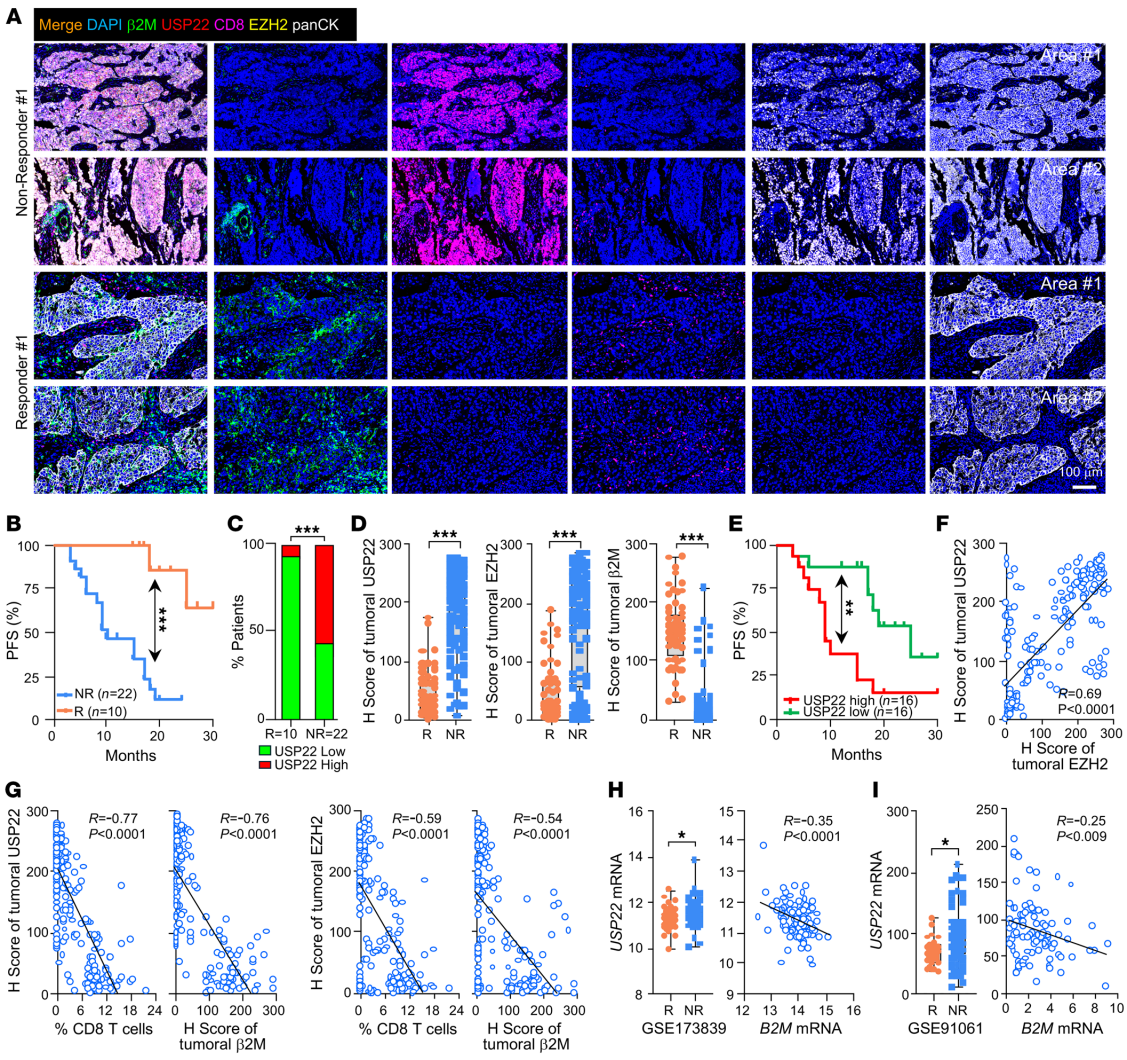
USP22 expression links with ICB resistance. (**A**) Representative images of multiplexed immunofluorescence staining of USP22/EZH2/b2M/CD8 in 32 pretreatment biopsies taken from individual patients who received anti-PD-1 antibody therapy. Scale bar: 100 μm. (**B**) Kaplan-Meier plot of progression-free survival (PFS) for 32 patients with NSCLC who did or did not respond to anti-PD–1 antibody therapy. (**C**) Patients were divided into USP22 low or high groups according to USP22 expression. Frequency of responder or nonresponder with high or low USP22 expressions are shown. R, responder; NR, nonresponder. (**D**) Quantification data of USP22/EZH2/b2M intensity in biopsies from anti-PD–1 responders or nonresponders. (**E**) Kaplan-Meier plot of PFS for patients treated with anti-PD–1 in USP22 low versus high group. Patients were classified into the USP22 low or high groups, with the median expression value across all the samples used as the cutoff. (**F** and **G**) Pearson correlation analyses between indicated proteins expression in biopsies from patients who did or did not respond to anti-PD–1 therapy. (**H**) The mRNA expression of *USP22* in pretreatment biopsies from patients with triple negative breast cancer who received anti-PD–1 therapy. Clinical responses were classified in the original studies GSE173839. Correlations between the mRNA expression of *USP22* and *B2M* are shown. (**I**) The mRNA expression of *USP22* in pretreatment biopsies with melanoma who received anti-PD–1 therapy. Clinical responses were classified in the original studies GSE91061. Correlations between the mRNA expression of *USP22* and *B2M* are shown. Statistics were calculated by unpaired 2-tailed *t* test (**D**, **H** and **I** (left panel)), Fisher exact test (**C**), Log rank *t* test (**B** and **E**), 2-tailed Pearson correlation test (**F** and **G**, and **H** and **I** (right panel)). *P < 0.05, **P < 0.01, and ***P < 0.001.

Poor neoantigen presentation, either due to low mutational load or reduced HLA-I expression, or both, is a critical driver of ICB resistance (1, 3). Our discovery that USP22-mediated MHC-I/HLA-I downregulation prompted us to evaluate the association of USP22 expression levels with ICB resistance. We collected lung biopsies from a cohort of 32 patients diagnosed with non-small cell lung cancer (NSCLC) prior to ICB immunotherapy with anti-PD–1 (sintilimab, tislelizumab, or camrelizumab; all of these have been approved by China national medical products administration for NSCLC treatment). After an up-to 30-month follow up of clinical immunotherapeutic studies, we confirmed that out of these 32 patients, 22 were clinically classified as nonresponders who were resistant to the anti-PD–1 ICB therapy and 10 were responders (Supplemental Table 1). The responders showed prolonged progression-free survival (PFS) relative to nonresponders (Figure 6B). Importantly, we found that high pretreatment expression of USP22 was predictive of ICB resistance (Figure 6C). As expected, a higher frequency of intratumoral CD8^+^ T cell infiltration and high tumor MHC-I expression was detected in responders compared with nonresponders (Figure 6, A and D, and Supplemental Figure 13A). Tumor tissues from ICB nonresponders exhibited higher levels of both tumoral USP22 and EZH2 expression and lower tumoral β2M expression when compared with biopsies from patients who were ICB responsive (Figure 6, A and D). These results suggest that elevated USP22 expression is a potential biomarker to predict ICB responsiveness in lung cancer. To further support this notion, USP22/EZH2/β2M levels and CD8^+^ T cell infiltration were associated with notable differences in PFS following ICB therapy regardless of ICB responsiveness (Figure 6E and Supplemental Figure 13, B–D).

In addition, consistent with our findings in breast, prostate, and colon cancers (Figure 5 and Supplemental Figures 10–12), tumoral USP22 and EZH2 protein expressions exhibited a strong positive correlation, and both were inversely correlated with tumoral β2M expression and with CD8^+^ T lymphocytes infiltration in lung cancer (Figure 6, F and G). We next explored the diagnostic significance of *USP22* expression in ICB responsiveness through unbiased analysis of RNA-seq data from a phase-II I-SPY2 trial, using durvalumab, olaparib, and neoadjuvant paclitaxel in patients with TNBC (29). The average levels of *USP22* transcripts were higher in nonresponders than in responders (Figure 6H), and USP22 transcript levels were inversely associated with levels of *B2M* transcripts (Figure 6H). Consistently, another RNA-seq dataset from patients with advanced melanoma treated with ipilimumab followed by nivolumab (30) revealed an increase in *USP22* transcripts in nonresponders compared with responders (Figure 6I), which were inversely correlated with *B2M* transcript levels (Figure 6I). These results further support our conclusion that increased USP22 is associated with ICB therapy resistance.

### Targeting USP22 overcomes ICB resistance.

**Figure 7:**
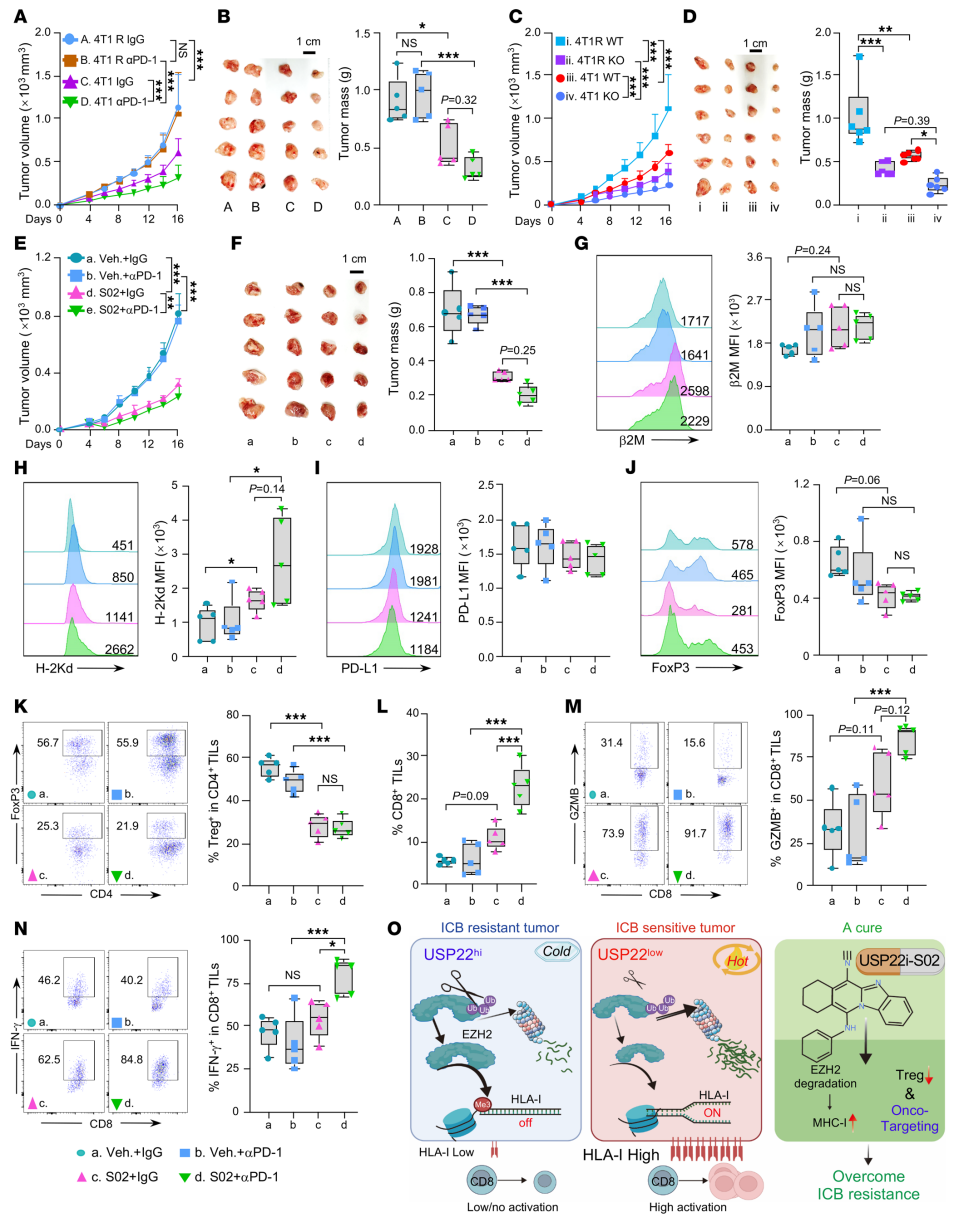
Targeting USP22 overcomes ICB resistance. (**A** and **B**) Effects of administration of anti-PD–1 on 4T1 or 4T1R tumor growth (**A**) and weight (**B**). Scale bar: 1 cm. Image of 4T1 or 4T1 R tumors treated with or without anti-PD–1 are shown. (**C** and **D**) Effects of *Usp22* deficiency on 4T1 or 4T1R tumor growth (**C**) and weight (**D**). (**E** and **F**) Effects of S02 or anti-PD–1 in 4T-1 R tumor growth. Mice were randomly grouped into 4 groups and administered with 10 mg/kg USP22i-S02 and/or 100 mg anti-PD–1. Mice were treated with 10 mg/kg S02 daily from day 4 to day 9, or were given a combination treatment with 100 μg anti-PD-1 antibodies every other day from day 4 to day 8. (**G**–**I**) Representative flow cytometric images and quantification data of cell surface b2M (**G**), H-2Kd (**H**), or PD-L1 (**I**) MFI in indicated tumor cells. (**J**) Representative flow cytometric images and quantification of FoxP3 MFI. (**K**) Representative images of flow cytometric analysis and quantification of frequencies of Tregs among total CD4^+^ lymphocytes in indicated tumors. (**L**) Quantification of frequencies of CD8^+^ T cells among tumor-infiltrating CD45^+^ lymphocytes in indicated tumors. (**M** and **N**) Representative flow cytometric images and quantification of frequencies of GZMB- (**M**) or IFN-γ– (**N**) producing tumor-infiltrating CD8^+^ T cells in indicated tumors. (**O**) Proposed working model showing that USP22 inhibition enhances antitumor immunity through increases in EZH2 proteasomal mediated degradation and MHC-I mediated. CD8^+^ T cells recognition and killing. Pharmacological USP22 inhibition overcomes immune checkpoint blockade resistance. Statistics were calculated by 1-way ANOVA followed by Tukey’s test (**B**, **D**, and **F**–**N**) or 2-way ANOVA with multiple comparisons (**A**, **C**, and **E**). *P < 0.05, **P < 0.01, and ***P < 0.001.

We then established a preclinical orthotopic TNBC 4T1 syngeneic model that is fully resistant to anti-PD–1 immunotherapy to test whether USP22-mediated MHC-I suppression is responsible for ICB resistance. Briefly, mice with preestablished orthotopic 4T1 TNBCs were treated with anti-PD–1 antibody when the tumor volume reached approximately 50–100 mm^3^. At day 18 after tumor inoculation, tumors were harvested, CD45^–^ tumor cells were isolated and cultured for 2–3 passages in vitro, and denoted as 4T1 cycle 1 (C1) (Supplemental Figure 14A). 4T1-C1 cells were then reimplanted followed by the same anti-PD–1 treatment regime. Following 3 sequential cycles (4T1-C3), the tumors exhibited complete resistance to anti-PD–1 treatment (Supplemental Figure 14A and Figure 7, A and B). We then named the anti-PD–1 resistant 4T1 tumor cells as 4T1R. Flow cytometry and Western blotting analysis of 4T1R cells detected a substantial reduction in MHC-I expression levels, with increased USP22 and EZH2 protein expression (Supplemental Figure 14, B and C). In contrast, the surface expression of checkpoint molecules PD-L1, CD73, and CD155 on 4T1R cells were slightly increased when compared with parent 4T1 cells (Supplemental Figure 14B). Further RT-PCR analysis detected a remarkable reduction in several key genes involved in antigen processing and presentation, including *B2m*, *H2D1*, *Tap1*, *Tap2*, and *Psmb9* in 4T1R cells (Supplemental Figure 14D). A remarkable increase in the mRNA expression of *Usp22* and *Ezh2* was detected in 4T1R cells compared with 4T1 cells (Supplemental Figure 14D). Consistent with the in vitro 4T1R characterization results, analysis of CD45^–^ cells from orthotopic 4T1R tumors relative to 4T1 tumors found decreased expression of MHC-I (Supplemental Figure 14E). Unexpectedly, cell surface PD-L1 levels were comparable between 4T1 and 4T1R cells (Supplemental Figure 14F). These results indicate that the increased USP22 expression, which reduces MHC-I, rather than the altered PD-L1 expression, is largely responsible for anti–PD-1 ICB therapeutic resistance.

Flow cytometric analysis of tumoral infiltrating lymphocytes revealed a reduced proportion of CD8^+^ T cells and decreased production of GZMB and IFN-γ in 4T1R tumors (Supplemental Figure 14G). We also noticed that 4T1R tumors showed increased frequencies of total CD4^+^ T cells and regulatory T cells (Tregs, CD4^+^CD25^+^FoxP3^+^), as well as a slight, but not statistically significant, increase of myeloid-derived suppressor cells (MDSCs, CD11b^+^Ly6G^+^) compared with 4T1 tumors (Supplemental Figure 14G). Additionally, we didn’t observe any changes in the frequency of natural killer cells (CD3^–^NK1.1^+^) (Supplemental Figure 14G). Therefore, these results indicate that USP22 represses MHC-I expression to architect an immune-suppressive tumor microenvironment with increased Tregs and MDSCs and decreased CD8^+^ T cells promoting ICB resistance.

We then asked whether *Usp22* inhibition is sufficient to overcome ICB resistance. Indeed, targeted *Usp22* ablation inhibited the growth of both 4T1R and 4T1 orthotopic tumors (Figure 7, C and D), suggesting that elevated *Usp22* expression is largely responsible to ICB resistance. Flow cytometry analysis of cell surface level of MHC-I on 4T1R USP22-null and control tumors showed that *Usp22* deficiency in 4T1R tumors led to increased MHC-I expression relative to 4T1 control tumors level (Supplemental Figure 14H). Consistently, *Usp22*-null 4T1R tumors exhibited a greater frequency of CD8^+^ T cells infiltration as well as a higher proportion of GZMB and IFN-γ producing CD8^+^ T cells (Supplemental Figure 14I).

Consistent with our results from targeted *Usp22* genetic deletion, treatment of mice with preestablished orthotopic 4T1R tumors by USP22i-S02 inhibited tumor growth (Figure 7, E and F). In contrast with our earlier finding that 4T1R is resistant to aPD-1, combined treatment with USP22i-S02 and anti-PD–1 further inhibited 4T1R tumor growth (Figure 7, E and F). Flow cytometry analysis revealed that USP22i-S02 treatment enhanced MHC-I, but not PD-L1 expression (Figure 7, G–I). In contrast, administration anti-PD–1 alone didn’t influence cell surface MHC-I or PD-L1 expression (Figure 7, G–I). Supporting our previous findings that USP22 acts as a Foxp3 stabilizer through deubiquitinating Foxp3 (9, 11), we found a reduction of Foxp3 mean fluorescence intensity as well as reduced percentages of intratumoral Foxp3^+^ Treg cells upon administration of USP22i-S02 (Figure 7, J and K). Consequently, USP22i-S02 in combination with anti-PD1 induced a greater frequency of CD8^+^ T cells compared with mice treated with either USP22i-S02 or anti-PD1 alone (Figure 7L). We also observed that either USP22i-S02 alone or in combination with anti-PD1 contributed to enhanced proportions of GZMB and IFN-γ–producing CD8^+^ T cells (Figure 7, M and N). In contrast, anti-PD–1 administration did not influence CD8^+^ T cells infiltration and function (Figure 7, L–N). Consistent with 4T1 R model, inhibition of *Usp22* in LLC1 cells, a well-established syngeneic tumor model that is resistant to ICB (31), inhibited tumor growth (Supplemental Figure 15, A and B). *Usp22* inhibition combined with anti-PD1 induced a greater tumor regression and resulted in a higher percentage of CD8^+^ T cell infiltration compared with mice treated with anti-PD1 alone (Supplemental Figure 15, A–C). Collectively, our findings reveal the USP22-EZH2-MHC-I axis driving tumor immune evasion. The upregulation of USP22, coupled with its inverse correlation with HLA-I expression and CD8^+^ T cell infiltration, positions USP22 as a potential biomarker for predicting resistance to ICB therapy. Furthermore, pharmacological inhibition of USP22 offers a promising strategy to overcome ICB resistance, providing a therapeutic avenue for the treatment of a wide range of human solid tumors (Figure 7O).

## Discussion

The current study has identified USP22 as a critical regulator responsible for poor MHC-I expression through potentiating EZH2-mediated epigenetic silencing. Furthermore, USP22 inhibition holds great potential to overcome the current limitations with immune checkpoint blockade therapy. This conclusion is supported by the following discoveries: first, immunostaining revealed a strong positive correlation between expression of USP22 and β2M in multiple types of human solid tumors including breast, colon, prostate, and lung cancers; second, both genetic and pharmacological USP22 inhibition increased MHC-I and HLA-I expression in mouse and human cancer cells, respectively; third, USP22 represses MHC-I expression through EZH2-mediated transcriptional silencing; fourth, EZH2 is a bona fide substrate of USP22 in human and mouse tumor cells; fifth, increased USP22 positively correlates with EZH2, but negatively correlates with HLA-I in human tumors, which predicts ICB response in lung adenocarcinoma patients; and, finally, USP22 inhibition overcomes anti-PD1 resistance in the treatment of orthotropic TNBC.

Tumor cells escape antitumor immune surveillance through inhibiting neoantigen presentation, such as downregulating the expression and function of MHC-I molecules, which are crucial for presenting antigens to cytotoxic CD8^+^ T cells (5). Direct mutations in the genes involved in the MHC-I or HLA-I pathway, such as β2M mutation, which can lead to reduced expression or absence of these molecules, have been identified in some cancer patients (32). Studies have implicated epigenetic and transcriptional silencing of MHC-I expression, such as through increased histone methylation mediated by proteins like EZH2, in the development and progression of many types of human cancers. Similarly, the EZH2-containing PRC2 transcriptional cosuppressive complex and other regulatory proteins keep chromatin in a transcriptionally inactive state, reducing the expression of MHC-I and antigen-processing components (8). Our study here identifies USP22 as a critical MHC-I repressor by protecting EZH2-mediated transcriptional inhibition of MHC-I transcription. At the molecular level, USP22 functions as an EZH2-specific deubiquitinase to protect EZH2 from ubiquitin-mediated proteasomal degradation. To support this conclusion, our immunostaining analysis detected a positive correlation between USP22 and EZH2, which were both negatively associated with tumoral β2M expression and CD8^+^ T cell infiltration in human breast, colon, prostate, and lung cancers. Interestingly, USP22 appears to selectively control EZH2 but not any other PCR2 complex proteins including EZH1, SUZ12, and EED in tumor cells. Therefore, our study defines USP22 as a EZH2-specific deubiquitinase to potentiate the epigenetic silencing of MHC-I gene expression. Consistent with our study, a comprehensive genome-wide profiling of the immune-evasive molecular signature of USP22 also identified USP22 as a negative regulator for downregulation of MHC-I in pancreatic tumor cells (33). Importantly, this elegant study also discovered the transcriptional suppressive function of EZH2 complex is regulated by USP22. Our discovery that EZH2 is a bona fide substrate provides a direct connection between USP22 and EZH2 in silencing tumoral MHC-I expression. In addition, a recent genome-wide CRISPR screening also identified USP22 as a hit in regulating MHC-I expression (34). Therefore, USP22-mediated MHC-I suppression appears to be a critical mechanism underlying tumor evasion of CD8^+^ T cell immunity in a variety of human cell types. In addition to USP22, the ubiquitin-like modifier activating enzyme 1 (UBA1) has been shown to downregulate MHC-I expression for tumor immune evasion (35). Moreover, several ubiquitin regulators including the epigenetic regulator ubiquitin-like with PHD and ring finger domains 1 (UHRF1) (36), RNF185 (37), and USP8 (38) regulate tumoral MHC-I expression. On the other hand, the ubiquitin-like protein 3 (UBL3) corporates with the E3 ligase MARCH to target MHC-II for ubiquitination (39). It will be interesting to further delineate first whether, and, then, how different ubiquitin pathways corporately control tumor neoantigen presentation through either MHC-I downregulation or neoantigen processing, or both, during immune evasion. In addition to MHC-I, it has been recently reported that EZH2 inhibition stabilizes PD-L1 expression through USP22-mediated deubiquitination, which is intriguing (40). Our study demonstrates that USP22 stabilizes EZH2, suggesting a potential feedback loop between USP22 and EZH2 in the regulation of PD-L1 expression and possibly also MHC-I. Moreover, the role of EZH2 in PD-L1 regulation appears to be context dependent. For instance, EZH2 has been reported to negatively regulate PD-L1 expression in hepatocellular carcinoma (41), while other studies have shown that shRNA-mediated EZH2 knockdown suppresses both mRNA and protein levels of PD-L1 L1 (42). These observations highlight the complexity of the regulatory network and underscore the need for further investigation into the roles of USP22 and EZH2 in modulating PD-L1 expression across different cancer types.

Cancer immunotherapy has indeed transformed the standard of care for many advanced cancers. However, clinical outcomes of cancer immunotherapy are still limited for most solid tumors. For example, the current checkpoint blockade immune therapy has so far proved disappointing in the treatment of colorectal cancers in the patient population (43). While CRCs have been classified as “cold tumors,” often characterized by low or absent PD-L1 expression, clinical findings indicate that approximately 60% of human CRCs exhibit PD-L1 positivity (44). Thus, the lack of PD-L1 expression does not seem to be the primary factor driving CRCs’ “cold tumor” status. In the case of patients with aggressive TNBC, anti-PD1 immune checkpoint inhibitors (such as pembrolizumab), when combined with chemotherapy, are now part of standard care for high-risk stage II/III and advanced PD-L1^+^ TNBC (45). The pathologic complete response (pCR) rate is 62% in patients with a PD-L1 combined positive score (CPS) greater than or equal to 1 and 50% in those with a PD-L1 CPS less than 1 (46). Yet a substantial proportion (up to 40% of PD-L1^+^ and 50% of PD-L1^low/–^) of TNBCs are classified as “cold tumors.” Therefore, PD-L1 expression does not seem to be the primary determinant of “cold tumor” status for both CRCs and TNBC. Indeed, the ICB-resistant 4T-1R TNBC cells show slightly higher PD-L1 and CD73 expression levels, both of which are USP22 targets (28, 47). Therefore, the reduced MHC-I expression due to increased USP22 appears to be the major driver of anti-PD-1 resistance. Importantly, we observe that all anti–PD-1 responding patients with lung cancer show statistically significant lower USP22 expression levels, which are reversibly associated with increased tumoral HLA-I (β2M) expression and CD8^+^ T cell infiltration. Therefore, our results suggest that USP22 expression levels alone or combined with HLA-I expression levels and CD8^+^ T cell infiltration frequency prior to ICB, could serve as a more accurate biomarker to predict the ICB immunotherapeutic response for lung cancer treatment. Cancer patients can exhibit either primary resistance (lack of initial response) or acquired resistance (loss of response after an initial benefit). Of note, the patients with heterogenous USP22 expression tumors (mixed with high and low expression) exhibit an initial responsiveness to ICB immunotherapy and ultimately develop acquired resistance to the treatment. While our data demonstrate a strong negative correlation between USP22 and β2M across multiple human cancer types, we were only able to recruit 32 patients with lung cancer to assess the predictive value of USP22 expression for anti–PD-1 ICB responsiveness. Future studies with larger cohorts and across additional cancer types will be necessary to establish USP22 expression as a reliable biomarker for predicting ICB therapy responsiveness.

Recent studies reveal 2 main factors that contribute to resistance to ICB therapy in cancer treatment: (a) an immunosuppressive TME due to increased infiltration of Tregs (48–50), myeloid-derived suppressor cells, and immune suppressive macrophages (51); and (b) impaired tumor antigen presentation due to relatively low mutational burden and reduced MHC-I expression (52). Hence, targeting immunosuppressive TME and enhancing neoantigen presentation are essential strategies to improve the efficacy of immunotherapy for treatment of tumors including TNBC and lung cancer. Importantly, our discovery here that tumoral USP22 inhibition increases MHC-I expression, together with our recent works that USP22 deletion diminishes protumor Treg suppressive activity (9, 11), indicate that USP22 plays a critical role in immunotherapeutic resistance. Furthermore, in cancer cells, USP22 promotes expression of PD-L1 and CD73 (28, 47), 2 checkpoint receptors responsible for tumor immune evasion. In addition to its immune evasive functions, elevated expression of USP22 correlates with poor prognosis in a variety of human tumors (53, 54) and functions as an oncogene by targeting cyclins, c-Myc, and p53, to inhibit apoptosis and promote cell cycle progression (17, 18, 55–58). USP22 also promotes chemotherapy resistance by inhibiting Bax-mediated apoptosis (59) and is reportedly a critical cancer stem cell gene (60). Indeed, we have previously demonstrated that USP22 is required to maintain TNBC stemness and that pharmacological USP22 inhibition reduces mouse 4T1 and human TNBC PDX metastasis to the lungs (19). These discoveries indicate that targeting USP22 enhances both immune boosting and oncotargeting dual efficacy in antitumor treatment and holds great potential to overcome the current limitations with ICB resistance. Indeed, our study here demonstrated that both genetic and pharmacological USP22 suppression improved the anti–PD-1 therapeutic activity in treatment of the orthotopic 4T1 TNBCs that are completely resistant to anti–PD-1, providing a strong rationale for USP22 targeting to overcome the ICB therapy resistance. This superior therapeutic efficacy of USP22 inhibition is, in part, through enhancing MHC-I–mediated CD8 cytotoxic activity. In addition to CD8^+^ T cells, MHC-I is a known suppressor of NK cell activation. The increase in MHC-I expression following USP22 inhibition may potentially reduce NK cell–mediated tumor killing. Interestingly, research from the Stanger lab (33) demonstrated that loss of USP22 in pancreatic ductal adenocarcinoma led to enhanced NK cell infiltration, suggesting that additional mechanisms may influence the impact of USP22 targeting on NK cell immunity, which deserve extensive future studies to explore the underlying cellular and molecular mechanisms. Our group is currently evaluating the preclinical efficacy of the first USP22-specific small molecule inhibitor in overcoming the ICB immunotherapeutic resistance for the treatment of a broad spectrum of solid tumors and conducting IND, enabling studies to translate a USP22-specific inhibitor from bench to bedside to treat human cancers.

## Methods

### Sex as a biological variable.

For human samples, both male and female patients were included for lung and colon cancers, while prostate cancer samples were obtained from male patients and breast cancer samples from female patients. In animal studies, both male and female mice were used for the LLC1 lung cancer and MC38 colon cancer syngeneic models. For the 4T1 TNBC orthotopic syngeneic model, only female mice were used, whereas only male mice were used for the RM1 syngeneic tumor model.

### Tumor model and maintenance of mice.

BALB/c, C57BL/6J, Rag1^–/–^, and OT-I C57BL/6J mice were purchased from the Jackson Laboratory and maintained in a specific pathogen-free facility. A total of 5 × 10^5^ RM1 or MC38 WT or *Usp22* KO cells were resuspended in 100 mL PBS and subcutaneously injected into the flank of C57BL/6J or Rag1^–/–^ mice aged at 7–8 weeks. A total of 5 × 10^5^ 4T-1 or 4T-1 R WT or *Usp22* KO cells were orthotopically injected into the mammary fat pad of BALB/c female mice aged at 7–8 weeks. Tumor volume was monitored every other day and calculated using the following formula: Tumor volume = length × width^2^/2. For the S02 treatment, 7–8 week-old C57BL/6J mice were subcutaneously inoculated with 5 × 10^5^ RM1 and MC38 cells. 24 hours later, mice were randomized into treatment groups. When the tumor volume reached around 50–100 mm^3^, mice were treated with S02 (10 mg/kg) and/or 100 μg PD-1 (Bio X Cell, BE0289) or IgG isotype antibody (Bio X Cell, BE0073), or vehicle control (10% DMSO) by intraperitoneal injection over 6 consecutive days. For 4T-1 cells, a total of 4T-1 WT or *Usp22* KO cells were orthotopically inoculated into the fourth MFP of 7–8 week-old female BALB/c mice. Mice were sacrificed by CO_2_ asphyxiation followed by cervical dislocation. Postmortem, tumors were immediately harvested and further processed for downstream experiments. For CD8^+^ T cell depletion assay, tumor-bearing mice were intraperitoneally injected with 100 μg of αIgG2b (BioXCell, Cat# BE090) or αCD8 clone 2.43 (BioXCell, Cat# BE0061) in PBS when tumor volume reached around 50–100 mm^3^.

### Lung cancer patient recruitment and biopsy collection.

Needle biopsies were collected from 32 newly diagnosed lung cancer patients (21 males and 11 females; mean age 59.4 years, range 44–71) prior to any therapy (immunotherapy, chemotherapy, or radiotherapy). Patients were subsequently treated with anti–PD-1 as specified in Supplemental Table 1 and followed for at least 3 months after treatment.

### Tumor-infiltrating T cells in vitro restimulation.

Tumor-bearing mice were sacrificed, and the tumors were harvested, photographed, and processed for further analysis. Collected tumor tissues were cut into small pieces and digested with 1 mg/mL Collagenase D (Worthington, Cat#: LS004189) and 50 μg/mL DNase I (Roche, Cat#10104159001) with gentle shaking for 30 minutes at 37°C. The digestion was stopped by EDTA (pH 8.0) and cells were filtered through 100 μm cell strainers. Afterward, samples were incubated with ACK buffer (Fisher, catalog no. A1049201) to lyse red blood cells, a total of 5 × 10^6^ cells were stimulated with 2.5 mg/mL Phorbol 12-Myristate 13-Acetate (PMA) and 10 mg/mL ionomycin, and blocked with monensin (eBiosciences, Cat#00450551) for 2–3 hours at 37°C. Cells were subjected to surface and intracellular staining as previously described in Flow Cytometry after washing twice with ice cold FACS buffer (PBS supplemented with 3% FBS). Indicated samples were evaluated on the BD LSRFortessa Cell Analyzer. All flow cytometry data were analyzed with FlowJo V10.8.1.

### OT-I CD8^+^ T cell killing assay.

Indicated RM1 or MC38 OVA-expressing cells were seeded in a 96-well plate at a density of 1 × 10^5^ per well. 4 hours after seeding, each well was gently washed twice with 100 mL PBS. 1 × 10^5^ freshly isolated splenic naive OT-I CD8^+^ T cells were cultured in complete T cell media supplemented with 0.5 μg/mL of anti-CD28 antibody. After culturing with CD8^+^ T cells for 48 hours, the supernatant of each well was carefully removed, and the cells were washed with PBS twice. Cells were fixed with 4% paraformaldehyde and adherent tumor cells were stained with 0.5 % crystal violet for 10 minutes at room temperature. After gently washing 6 times with PBS, the OD_450_ values were evaluated by a spectrometer.

### Tissue microarray.

Tissue microarrays (TMA) with detailed clinical and pathological information were purchased and conducted by Shanghai YEPCOME Biotech Co., Ltd. The TNBC TMA included 163 samples, consisting of 133 breast cancer tissues and 30 adjacent healthy controls, all from female patients (mean age 54.4 years, range 26–78). The prostate cancer TMA comprised 91 prostate cancer tissues and 53 matched paratumor tissues, all from male patients (mean age 70.7 years, range 55–84). The colon cancer TMA contained 80 paired tumor and paratumor tissues from 43 male and 37 female patients (mean age 66.5 years, range 31–88).

### IHC.

For IHC staining, tissue specimens were deparaffinized in xylene, rehydrated through graded ethanol solutions, subjected to antigen retrieval, and immersed in a 0.3% hydrogen peroxide solution. Slides were washed thrice with phosphate-buffered saline (PBS) and blocked with 5% bovine serum albumin for 30 minutes at room temperature. The tissue slides were subsequently incubated with primary antibodies overnight at 4°C. HRP conjugated secondary antibody was used to incubate the slides before DAB detection. The quantification analysis of IHC results in multiplexed immunofluorescence. The detailed information of antibodies used in IHC were listed in Supplemental Table 3.

### Multiplexed Immunofluorescence (mIF).

A multiplexed immunofluorescence (mIF) panel comprising β2M, USP22, EZH2, and CD8^+^, panCK and nuclear marker (DAPI), was developed. A TSA (tyramide signal amplification) approach was employed in multiplexed immunofluorescence staining according to our previous report (61). The staining procedure contained sequential cycles of antigen retrieval, nonspecific antigen blocking, primary antibody incubation, secondary antibody incubation, and fluorescent labeling via TSA on whole slides. Briefly, tumor tissues were cut into 4 μm pieces and adhered to microscope slides. Tumor tissues were subsequently incubated with 3% H_2_O_2_ solution for 20 minutes. Sections were then incubated with 10% bovine serum albumin for 20 minutes at room temperature and incubated with antibodies at 4°C overnight in the dark. Samples were rinsed 3 times with PBST (PBS supplemented with 0.2% Tween-20) before incubating with the indicated horseradish peroxidase (HRP) conjugated secondary antibody (1 mg/mL) for 2 hours at room temperature. This was followed by 3 washes with PBST and incubation with tyramide staining dye for 15 minutes at room temperature. Finally, the slides were counterstained with 1 μg/ml DAPI (Life technology) for 5 minutes.

### Quantification of mIF.

USP22/EZH2/β2M expression was quantified specifically in panCK^+^ cells. Briefly, quantification analysis was double-blindly performed using digital image analysis and spectral unmixing HALO software, which isolates individual panCK-positive cells and quantitatively measures USP22/EZH2/β2M expression in panCK-positive tumor cells. The panCK-negative CD8^+^ T cells were also quantified. For each case, 6 random fields (200 × 200 μm per field), containing on average 200–250 cells per field, were analyzed. Quantification was performed using the following formula: H-Score = ∑(*pi* × *i*) = (percentage of negative cells × 0) + (percentage of weak-intensity cells × 1) + (percentage of moderate-intensity cells × 2) + (percentage of strong-intensity cells × 3). Here, 0, 1, 2, and 3 correspond to negative, weak, moderate, and strong expression, respectively, while *pi* and *i* represent the percentage of positive cells and the staining intensity, respectively. The percentage (*pi*) was automatically calculated using HALO software.

More information of methods can be found in supplemental information.

### Statistics.

All sample numbers (*n*) represent biological replicates. Data are represented as the mean ± SD, and error bars indicate standard deviation. Differences with *P* values less than 0.05 were considered significant. All analyses were performed using GraphPad Prism software (GraphPad Software, Inc.). Student’s 2-tailed *t* test was used for comparisons of 2 groups. One-way ANOVA was used for comparisons among more than 2 groups. Two-way ANOVA was used for comparisons tumor growth and survival analysis.

### Study approval.

Human sample collection and use strictly followed the principles of the Declaration of Helsinki and were approved by the Institutional Review Board of the 920th Hospital of the Joint Logistics Support Force, Kunming, China (IRB#2020-035-01). All animal studies were conducted in accordance with protocols approved by the Institutional Animal Care and Use Committee of Northwestern University, Chicago, Illinois, USA (IACUC#IS00029963). Written informed consent was obtained from all participants or their legal guardians. Detailed donor characteristics are provided in Supplemental Table 1.

### Data availability.

The raw data are available to academic researchers from the corresponding author upon reasonable request. Values for all data points in graphs are reported in the Supporting Data Values file.

## Author contributions

All authors read and approved of the final manuscript. DF designed and supervised this project, and wrote the manuscript. KL performed most of the experiments and wrote the manuscript with input from ZC, YC, JW, NM, and HW. RI, AYT, QG, NLM, and BG edited the manuscript and analyzed the data. YL and JZ collected the clinical NSCLC samples. DMW, SAK, WJG, and HL revised the manuscript.

## Funding support

This work is the result of NIH funding, in whole or in part, and is subject to the NIH Public Access Policy. Through acceptance of this federal funding, the NIH has been given a right to make the work publicly available in PubMed Central.

- The Robert H. Lurie Comprehensive Cancer Center of Northwestern University (to KL).
- The National Institutes of Health (NIH) grants R01DK126908, R01DK120330, R01CA257520, R01CA284740 and CA232347 (to DF) and T32GM105538 and T32GM149439 (to AYT).

## Supplementary Material

Supplemental data

Unedited blot and gel images

Supporting data values

## Version 1 11/18/2025

In-Press Preview

## Version 2 01/02/2026

Electronic publication
