# Supplementary material for: USP22 drives tumor immune evasion and checkpoint blockade resistance through EZH2-mediated epigenetic silencing of MHC-I
Source: J Clin Invest. 2025 Nov 18;136(1):e193162. doi: 10.1172/JCI193162 (PMC12721901; doi:10.1172/JCI193162)
Supplement: Supplemental data [file jci-136-193162-s315.pdf]

USP22 drives tumor immune evasion and checkpoint blockade resistance through EZH2-mediated epigenetic silencing of MHC-I

Kun Liu[1], Radhika Iyer[1], Yi Li[2], Jun Zhu[2], Zhaomeng Cai[1], Juncheng Wei [3], Yang Cheng[1], Amy Tang[1], Hai Wang[1], Qiong Gao[1], Nikita Lavanya Mani[1], Noah Marx[1], Beixue Gao[1], D. Martin Watterson[4], Seema A. Khan[5], William J Gradishar [6], Huiping Liu [4, 7] and Deyu Fang[1*]

1. Department of Pathology, Robert H. Lurie Comprehensive Cancer Center and Center for Human Immunology, Northwestern University Feinberg School of Medicine, Chicago, IL, 60611, United States.

2. Department of Oncology, 920th Hospital of Joint Logistics Support Force, Kunming, China.

3. Center of Metabolic Disease Research, Department of Cardiovascular Sciences, Temple University Lewis Katz School of Medicine, Philadelphia, PA, 19140, USA.

4. Department of Pharmacology, Feinberg School of Medicine Northwestern University, Chicago, IL, 60611, United States.

5. Division of Breast Surgery, Robert H. Lurie Comprehensive Cancer Center, Feinberg School of Medicine, Northwestern University, 303 E Superior, 4-220, Chicago, IL 60611, USA.

6. Division of Hematology/Oncology, Department of Medicine, Feinberg School of Medicine, Northwestern University, Chicago, Illinois.

7. Department of Medicine, Division of Hematology and Oncology, Northwestern University Feinberg School of Medicine, Chicago, Illinois.

Key words: USP22, ubiquitination, EZH2, epigenetic regulation, MHC-I, ICB resistance.

Running title: Targeting USP22 overcomes ICB resistance.

Correspondence: fangd@northwestern.edu

Supplementary methods

Cell culture and transfection. PC3 cells were a kind gift from Dr. Bin Zhang at Northwestern University. Mouse splenocytes were cultured in RPMI media supplemented with 10% FBS, 2 mM Glutamine, 5% Penicillin/Streptomycin, 10 mM HEPES, 100 μM NEAA (Non-Essential Amino Acids), 10 U/ml IL-2, 50 μM β-mercaptoethanol. 4T-1 and PC3 cells were maintained in RPMI medium supplemented with 10% FBS, 2 mM Glutamine, 5% Penicillin/Streptomycin, 10 mM HEPES, 100 μM NEAA. HEK-293T, MC38, RM1, and MDA-MB-231 cells were cultured in DMEM medium supplemented with 10% FBS, 1% Penicillin and Streptomycin at 37 °C in a 5% CO2 humidified incubator.

Plasmids, shRNA and CRISPR/Cas9 genome editing. Ezh2 shRNAs were a kind gift from Dr. Jindan Yu at Northwestern University (78). Independent oligonucleotide sequences of Ezh2 shRNA were ligated with the pLKO.1 vector (#10878, Addgene). For the reconstitution of Ezh2/Ezh2 mutants, pMCSV containing mEzh2 (#24926), mEzh2 F667I (#24927) or mEzh2 ΔSET (#49403) plasmid was purchased from Addgene. pcDNA3.1 plasmids containing full length chicken ovalbumin was purchased from Addgene (#64599) and subcloned into pLV vector. Single guide RNA targeting *Usp22* or *B2m* were inserted into lentiCRISPR v2 (#52961). The detailed sequences of sgRNAs used in this study are shown in the supplementary table 2. The lentiviruses were produced from HEK-293T cells by transfecting with lentiviral packaging plasmids, psPAX2, and pMD2.G. Lentiviruses were collected 48 and 72 hours after transfection and filtered with 0.45 μm filters. Indicated target cells were infected with indicated lentiviruses in combination with 2.5 μg/ml of polybrene (Sigma-Aldrich, Cat#H9268). Two days after viral infection, transduced cells were selected with 2 μg/mL puromycin for 7 days. The selected positive cells were used for the further experiments.

RNA extraction and real time PCR. Total RNA was extracted from indicated tumor cells by Trizol reagent (Invitrogen, USA) according to the manufacturer protocols. Complementary DNA was generated by reverse transcriptase (Quantabio, Cat#66255946). Quantitative RT-PCR was then conducted by ChamQ SYBR

qPCR Master Mix (Invitrogen, A46012) on a LightCycler 480 instrument (Bio-Rad). The primers used in this study are listed in Supplementary Table 2.

Flow cytometry. For cultured cell lines, cells were dissociated with the Accutase Cell Detachment Solution (Corning, 25-058Cl), spun down and were rinsed with ice cold PBS. Prior to surface staining, cells were stained with Fixable Viability Dye eFluor 450 (eBiosciences, Cat#: 65086314). Subsequently, the cells were stained with indicated antibodies diluted in FACS buffer (PBS supplemented with 4% FBS) for 1 hour in the dark on ice. For intracellular staining, cells were then washed with ice cold PBS, spun down, and resuspended in fix buffer (eBiosciences, Cat#: 00522356 and 00522343) after surface staining. Cells were then incubated with indicated antibodies for 1 hour in the dark at room temperature with permeabilization wash buffer (eBiosciences, Cat#: 00833356). After rinsing twice with ice cold PBS, cells were then analyzed on an FACSCanto II cytometer (BD) and FlowJo (Tree Star). Antibodies used for flow cytometry are listed in supplemental table 3.

Immunoblot. Cells were lysed in RIPA buffer (50 mM Tris-HCl, pH 8.0, 150 mM sodium chloride, 1.0% NP-40, 0.5% sodium deoxycholate, 0.1% sodium dodecyl sulfate (SDS), protease inhibitor cocktail) for 30 minutes on ice. The supernatant was collected by centrifugation at 15,000 rpm/min for 20 min at 4°C. The same amount of protein was loaded and separated by SDS-polyacrylamide gels. Following transfer to a nitrocellulose membrane, membranes were blocked with 10% milk for 1 hour. Membranes were probed with indicated primary antibodies at 1:1000 dilution at 4°C overnight, washed with TBST buffer (TBS buffer with 0.1% Tween) for three times. The membranes were then incubated with goat anti-rabbit or anti-mouse horseradish peroxidase (HRP)-linked secondary antibody for 1 hour at room temperature and followed by washing with TBST for another three times. Chemiluminescent substrate (MYBioSource, Cat#MBS9719195) was then applied to the visualization of membrane by the ChemiDoc XRS + Imaging System (Bio-Rad). Antibodies used in immunoblot were included in supplementary table 3.

Cell proliferation. RM1 and MC38 cells with varying *USP22* or *B2m* expressions were cultured in 12 well plates in triplicate at a seeding density of 10^4, cell number for each day were evaluated by an automatic cell counter Cellmeter K2 (VC-660-503).

Colony formation assay. For colony formation assay, a total of 500 RM1 cells with varying *USP22* or *B2M* expression status were seeded into 6 well plates. After two weeks, cells were fixed with 4% PFA and then stained with 0.1 % crystal violet for 20 minutes at room temperature.

GST pull-down. The DNA sequence encoding mouse USP22 was cloned into vector pGEX6P-1. The recombinant plasmids were transformed into *E. coli BL21* (DE3). 0.5 mM isopropyl b-D-1-thiogalactopyranoside was added to induce protein expression at 18 °C when cells reached A_{600} = 0.8 at 37 °C. After 15 h induction, cells were harvested by centrifugation using 4,000 g for 30 min at 4 °C. Cells were resuspended in lysis buffer (20 mM Hepes pH 7.5, 500 mM NaCl, 5% glycerol, 20 mM imidazole, 1 mM DTT and 1 mM PMSF) and then sonicated. Cellular debris was removed by centrifugation for 60 min at 10,000 g at 4 °C. The supernatant was mixed with Glutathione Magnetic Agarose Beads (Thermo, Cat#78602) for 2 h, and then the beads were washed three times with lysis buffer. The proteins were incubated with 4T1 cells total cell extract at 4 °C overnight. The input and output samples were loaded onto an SDS-PAGE gel and detected by western blot.

Co-immunoprecipitation. Indicated cells were lysed in lysis buffer (50 mM Tris-HCl (pH 8.0), 150 mM NaCl, 0.5% NP-40, 1 mM phenylmethylsulfonyl fluoride, and 1× protease inhibitor) for 30 minutes on ice. The supernatant was collected by centrifugation at 15,000 rpm/min for 20 min at 4°C. The supernatant was pre-cleared by incubating with protein A/G sepharose beads (Cytiva, 10338420) for 30 minutes with gentle rotation at 4°C. Subsequently, 250 μg of protein was incubated with 2 μg antibodies overnight at 4°C. Followed by incubating with protein A/G sepharose beads for an additional 2 hours, the protein was gently

washed three times with a lysis buffer for three times. The precipitated proteins were eluted from the beads with a 2×SDS loading buffer and boiled for 5 min, followed by immunoblot analyses.

Enzyme-linked immunosorbent assay. The concentrations of TNF-α and IFN-γ in the supernatant of indicated cells co-cultured with $CD8^+$ T cells were determined by "sandwich" ELISA kits according to the manufacturer's instructions (SMTA00B and SMIF00, R&D Systems). Briefly, supernatant from indicated cancer cells co-cultured with isolated naïve OT-I $CD8^+$ T cells were collected. Subsequently, the supernatants and standard samples were added into wells precoated with either IFN-γ or TNF-α antibodies and incubated at room temperature for two hours. Detection antibodies were added followed by the addition of HRP-labeled streptavidin, substrate solution, and stop solution. Absorbance was determined and the exact concentrations of IFN-γ or TNF-α were calculated using the standard curve.

T cell co-culture assay. For indicated co-culture assays of naïve OT-I $CD8^+$ T cells with indicated cancer cells, a total of 1×10^5 RM1 or MC38 OVA expressing cells were seeded into 96-well plates with DMEM or RPMI media supplemented with 10% FBS and 5% Penicillin/Streptomycin, respectively. Spleens from OT-I mice were mechanically crushed and strained through a 100 μm cell strainer, and then incubated with ACK buffer (Fisher, catalog no. A1049201) to lyse red blood cells. Naïve $CD8^+$ T cells were isolated using EasySep Mouse $CD8^+$ T Cell Isolation Kit (STEMCELL, Cat#19853). Above 98% purity was confirmed by flow cytometry. A total of 1×10^5 $CD8^+$ T cells were then cocultured with indicated tumor cells for 8 hours in T cell medium (RPMI medium supplemented with 10% FBS, 2 mM Glutamine, 5% Penicillin/Streptomycin, 10 mM HEPES, 100 mM NEAA, 10 U/ml IL-2, and 0.5 μg/ml anti-CD28). For USP22i-S02 treatment, RM1 or MC38 OVA expressing cells were treated with 20 μM S02 or DMSO for 48 hours. Post S02 treatment, cells were washed twice with PBS and seeded at a density of 1×10^5 cells/well across a 96 well plate. 4 hours after seeding, each well was gently washed with 100 μL PBS. A total of 1×10^5 splenic naïve OT-I $CD8^+$ T were co-cultured with indicated RM1 or MC38 OVA expressing cells in complete T cell media. Subsequently, $CD8^+$ T cells were carefully collected and washed twice with FACS buffer and then subjected

to surface and intracellular staining as previously described. T cells co-cultured with indicated cancer cells were evaluated on the BD LSRFortessa™ Cell Analyzer. For intracellular cytokine staining, monensin was added to block cytokine secretion and incubated for 2 hours before collection.

Definition of responsiveness of immunotherapy. Specimens of lung cancer patients were collected from 920th Hospital of Joint Logistics Support Force, Kunming, China. All patient specimens were obtained either from diagnostic biopsies or surgical resections performed prior to the initiation of chemotherapy or other related treatments. A total of 32 patients with non-small-cell lung cancer were included in this study. Patients who received immunotherapy were divided into responder or non-responder based on the responsiveness according to RECIST criteria version 1.1 (https://www.recist.com/). Responder group includes patients who experienced partial response (PR) or complete response (CR). Non responder group includes patients who experienced disease progression (PD) or stable disease (SD) < 6 months as best response (RECIST 1.1). For the progression-free survival (PFS) measures, we calculated from the date of treatment start to the date of clinical or radiologic progression or death or last follow-up. Follow-up and survival times were calculated according to Kaplan Meier. PFS between groups were calculated by Log-Rank test (Mantel-Cox). Detailed clinic-pathological characteristics were shown in supplemental table 1.

Chromatin immunoprecipitation (Ch-IP) qPCR assay. A total of 5×10^6 indicated cells were cross-linked with 1% formaldehyde for 10 min at 37°C, and the reaction was stopped by the introduction of glycine. The cells were then washed with PBS and suspended in SDS-lysis buffer (50 mM Tris-HCl, 1% SDS, 10 mM EDTA, pH 8.1). The cells lysates were treated with MNase and subsequently sonicated to shear the DNA into fragments of 300-1000bp. After centrifugation, supernatants were incubated with specific antibodies against EZH2 or H3K27me3 at 4 °C overnight with gentle rotation. After reversing the cross-linking of protein/DNA complexes to free DNA, quantitative PCR was used to detect the enrichment of the indicate gene promoter sequences using gene-specific primers and SYBRGreen (Applied Biosystems). Primers for Ch-IP-PCR are provided in Supplementary Table 2

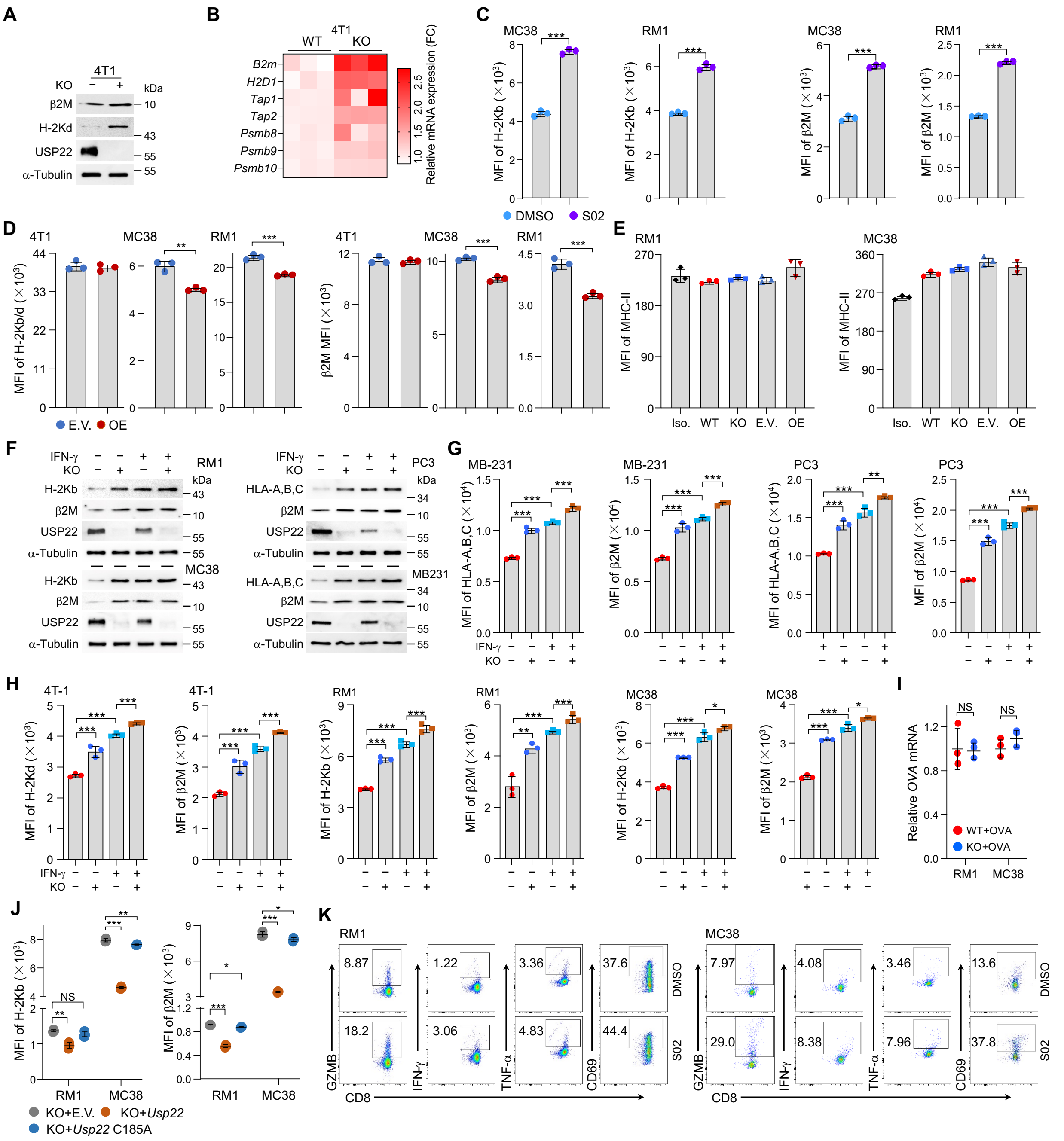

Supplementary Figure 1. *Usp22* ablation enhances MHC-I expression. (A) Immunoblot analysis of indicated proteins in 4T1 WT or *Usp22* KO cells. **(B)** Heatmap summarizing the qRT-PCR results for mRNA expression of the indicated genes normalized to the mean level of each gene in 4T1 cells with or without *Usp22* deficiency. *β-actin* was used as internal control. **(C)** The quantification analysis of flow cytometric analysis of cell surface expression of H-2Kb or β2M on indicated tumor cells treated with or without 20 μM

S02 for 48 hours. MFI, mean fluorescent intensity. **(D)** The quantification analysis of flow cytometric analysis of cell surface expression of H-2Kb/d and β2M in indicated cells with or without *Usp22* overexpression. **(E)** The quantification analysis of flow cytometric analysis of cell surface expression of MHC-II in indicated cells in the presence of *Usp22* depletion or overexpression. **(F)** Immunoblot analysis of indicated proteins in indicated cells treated with or without 10 ng/mL IFN-γ for 48 hours. **(G and H)** The quantification analysis of flow cytometric analysis of cell surface expression of HLA-A, B, C or β2M on indicated human cancer cells treated with or without 10 ng/mL IFN-γ for 48 hours. **(I)** Relative *ovalbumin* mRNA expression in RM1 or MC38 cells with stable *ovalbumin* overexpression. *Ovalbumin* expression was normalized to the mean level in RM1 or MC38 *Usp22* depletion cells. *Actb* was used as internal control. **(J)** The quantification analysis of cell surface level of H2-Kb and β2M in RM1 or MC38 *Usp22* KO cells with or without *Usp22* or catalytically inactive mutant (*Usp22*-C185A) restoration. E.V indicates empty vector. **(K)** RM1/OVA or MC38/OVA cells were pre-treated with 20 μM USP22i-S02 for 48 h and co-cultured with fresh isolated naïve OT-I $CD8^+$ T cells at a ratio of 1:1 (T cell: tumor cell) for 8 hours. Representative images of flow cytometric analysis of GZMB, IFN-γ, TNF-α, and CD69 in $CD8^+$ T cells are shown. Data are presented as the Mean ± SD. **P*< 0.05, ***P*< 0.01, and ****P*< 0.001 Statistics were calculated by unpaired two-tailed t-test (C, D, I) or one-way ANOVA followed by Tukey's (E, G, H) Dunnett’s test (J).

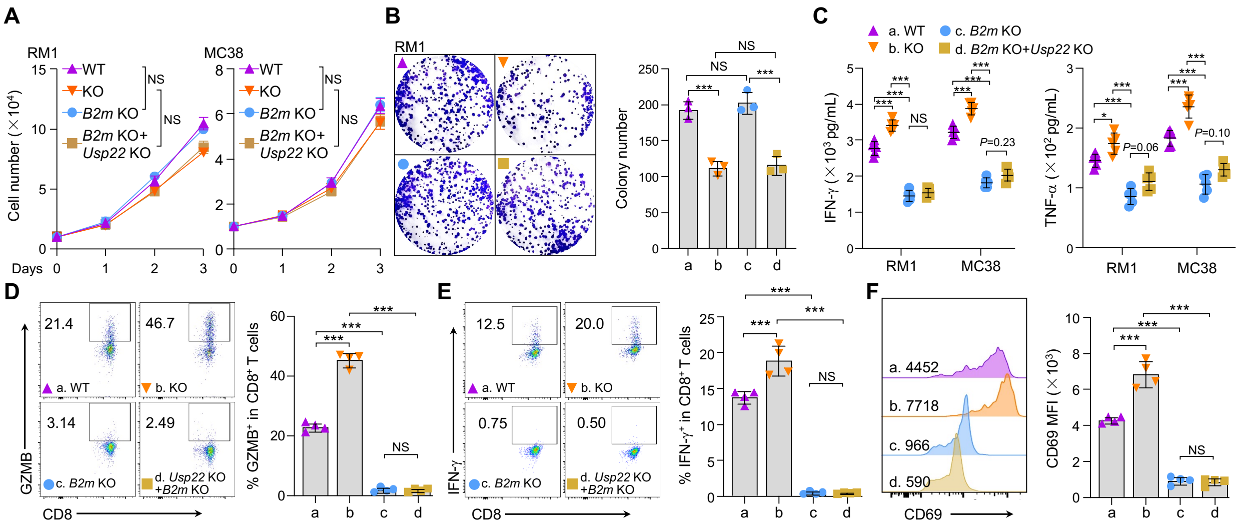

Supplementary Figure 2. Depletion of *B2m* abolishes *Usp22* inhibition-mediated tumor suppressive effects. (A) The exact cell number of indicated cells with varying *Usp22* or *B2m* expression status was recorded at indicated time points. **(B)** Representative images of colony formed from RM1 cells with varying *Usp22* or *B2m* expression status. The quantification data are shown. Scale bar, 1 cm. **(C)** The exact concentrations of IFN-γ or TNF-α in the supernatant of naïve OT-I CD8$^+$ T cells co-cultured with RM1 or MC38 with varying *Usp22* or *B2m* expression status for 48 hours were determined by ELISA. **(D and E)** Naïve OT-I CD8$^+$ T cells were co-cultured with RM1 OVA expressing cells with varying *Usp22 or B2m* expression status at a ratio of 1:1 (T cell: tumor cell) for 8 hours in the presence of CD28 blocking antibodies treatment. Representative images of flow cytometric analysis and quantification of GZMB (D), IFN-γ (E) producing or CD69 expressing (F) CD8$^+$ T cells. **P*< 0.05, ***P*< 0.01, and ****P*< 0.001. Statistics were calculated by paired two-way ANOVA with multiple comparisons (A), one-way ANOVA followed by Tukey's test (B-F).

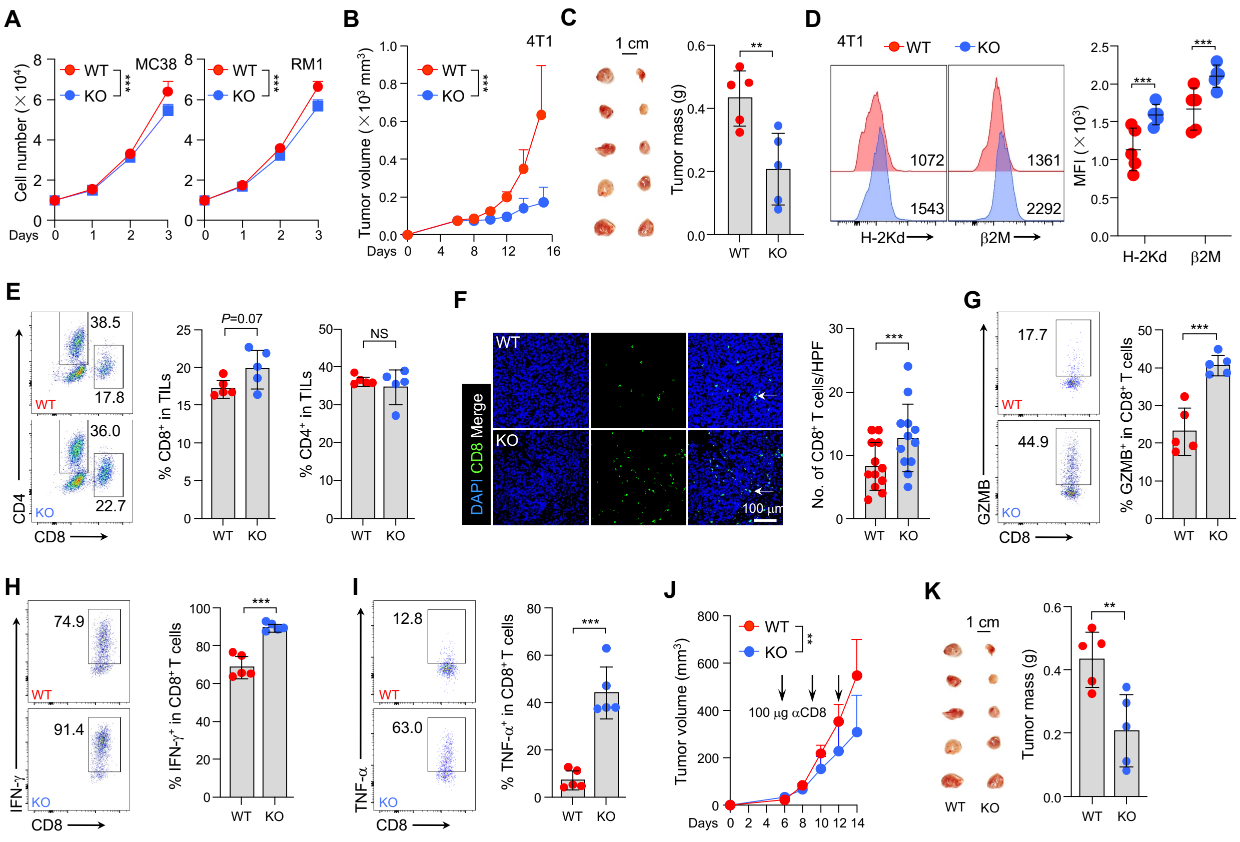

Supplementary Figure 3. *Usp22* inactivation improves anti-tumor immunity. (A) Effects of *Usp22* deficiency on in vitro cell proliferation of MC38 and RM1 cells. The exact cell number was counted every day. n = 3. **(B and C)** Effect of *Usp22* on tumorigenesis of 4T1 cells in BALB/c mice. A total of 5×10^5 4T1 WT or *Usp22* depletion cells were s ubcutaneously inoculated into BALB/c mice. Tumor volume (B) and endpoint weight (C) were recorded. **(D)** Representative images of flow cytometric analysis of tumoral cell surface level of H-2Kb and β2M on 4T1 WT or *Usp22* KO tumors. Quantification showing that *Usp22* deficiency resulted in an elevation of tumoral cell surface expression of H-2Kb and β2M. **(E)** Representative flow cytometric images and quantification of tumor-infiltrating $CD4^+$ or $CD8^+$ T cells in 4T1 tumors with or without *Usp22* deficiency. **(F)** Representative images of immunofluorescence staining of tumoral-infiltrating $CD8^+$ T cells in 4T1 WT or *Usp22* KO tumors. Scale bar, 100 μm. **(G-I)** Representative images of flow cytometric analysis of GZMB (G), IFN-γ (H), or TNF-α (I) producing $CD8^+$ T cells. **(J and K)** Effect of *Usp22* depletion on tumorigenesis of 4T1 cells in BALB/c mice in the presence of injection of αCD8 blocking antibodies. A total of 5×10^5 4T1 with or without *Usp22* depletion cells were subcutaneously inoculated into

BALB/c mice and administered with 100 μg αCD8 blocking antibodies at day 6, tumor volume (J) and endpoint weight (K) were recorded. Photograph of tumors shown. Scale bar: 1 cm. Data are presented as the Mean ± SD. *$P < 0.05$, **$P < 0.01$, and ***$P < 0.001$. Statistics were calculated by unpaired two-tailed t-test (C-I, K) or paired two-way ANOVA with multiple comparisons (A, B, J).

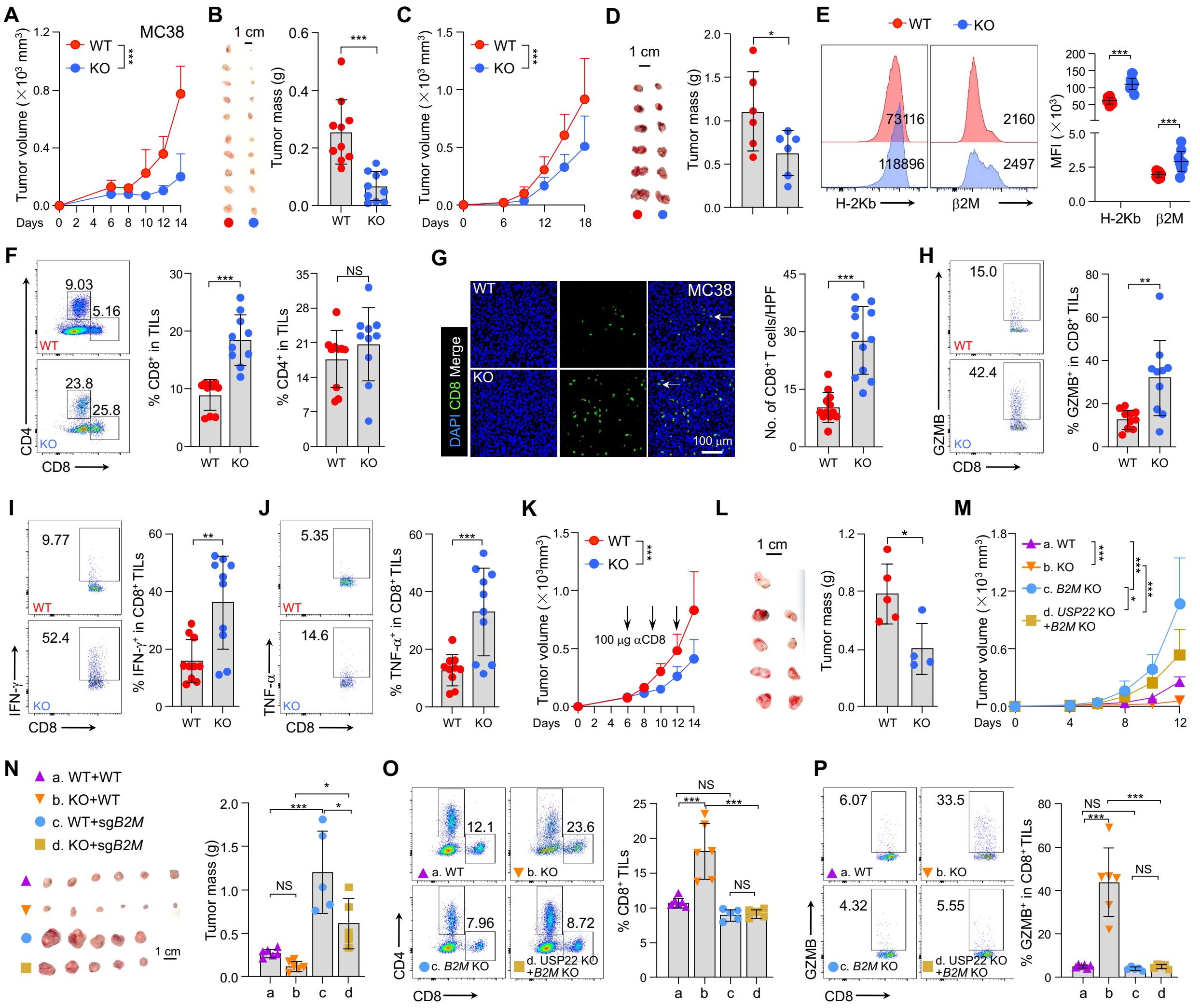

Supplementary Figure 4. *Usp22* inactivation improves anti-tumor immunity. (A-B) Effect of *Usp22* depletion on tumorigenesis of MC38 cells in C57BL/6 mice. A total of 5×10^5 MC38 cells with or without *Usp22* depletion were subcutaneously inoculated into C57BL/6 mice, tumor volume (A) and endpoint weight (B) were recorded. The image of tumors was shown. Scale bar: 1 cm. **(C-D)** Effect of *Usp22* depletion on tumorigenesis of MC38 cells in RAG1 knockout mice. A total of 5×10^5 MC38 cells with or without *Usp22* depletion were subcutaneously inoculated into RAG1 knockout mice, tumor volume (C) and endpoint weight (D) were recorded. Photograph of tumors shown. Scale bar: 1 cm. **(E)** Flow cytometric analysis of the expression of tumoral cell surface H-2Kb or β2M on MC38 tumors with or without *Usp22* deficiency. **(F)** Flow cytometric analysis of percentages of tumor-infiltrating CD4⁺ or CD8⁺ T cells among the total tumor-infiltrating CD45⁺ lymphocytes in MC38 tumors with or without *Usp22* depletion. **(G)** The representative

immunofluorescence staining images depict the infiltration of $CD8^+$ T cells in MC38 *Usp22* depletion or control tumors. Arrows denote $CD8^+$ T cells. Scale bar: 100 μm. Quantification showing that *Usp22* depletion in MC38 cells robustly augments $CD8^+$ T cells infiltrated into tumor. HPF indicates high powered field. **(H-J)** Flow cytometric analysis of percentages of granzyme B^+ (H), IFN-γ^+ (I) or TNF-α^+ (J) producing $CD8^+$ in MC38 tumors with or without *Usp22* depletion. Representative images and quantification of flow cytometric analysis are shown. **(K and L)** Effect of *Usp22* depletion on tumorigenesis of MC38 cells in C57BL/6 mice in the presence of injection of αCD8 blocking antibodies. A total of 5×10^5 MC38 cells with or without *Usp22* depletion were subcutaneously inoculated into C57BL/6 mice and administered with 100 μg αCD8 blocking antibodies at day 6, tumor volume (K) and endpoint weight (L) were recorded. The image of tumors was shown. Scale bar: 1 cm. **(M and N)** Effect of *Usp22* or *B2m* depletion on tumorigenesis of MC38 cells in C57BL/6 mice. A total of 5×10^5 MC38 with varying *Usp22* or *B2m* expression status were subcutaneously inoculated into C57BL/6 mice, tumor volume (M) and endpoint weight (N) were recorded. The image of tumors was shown. Scale bar: 1 cm. **(O and P)** Flow cytometric analysis of percentages of tumor-infiltrating $CD8^+$ T cells or GZMB producing $CD8^+$ T cells in MC38 tumors with varying *Usp22* or *B2m* expression status. Representative images and quantification of flow cytometric analysis are shown. Data are presented as the Mean ± SD. NS indicates no significance. *$P < 0.05$, **$P < 0.01$, and ***$P < 0.001$. Statistics were calculated by unpaired two-tailed t-test (B, D-J, L), paired two-way ANOVA with multiple comparisons (A, C, K, M), one-way ANOVA followed by Tukey's test (O-P).

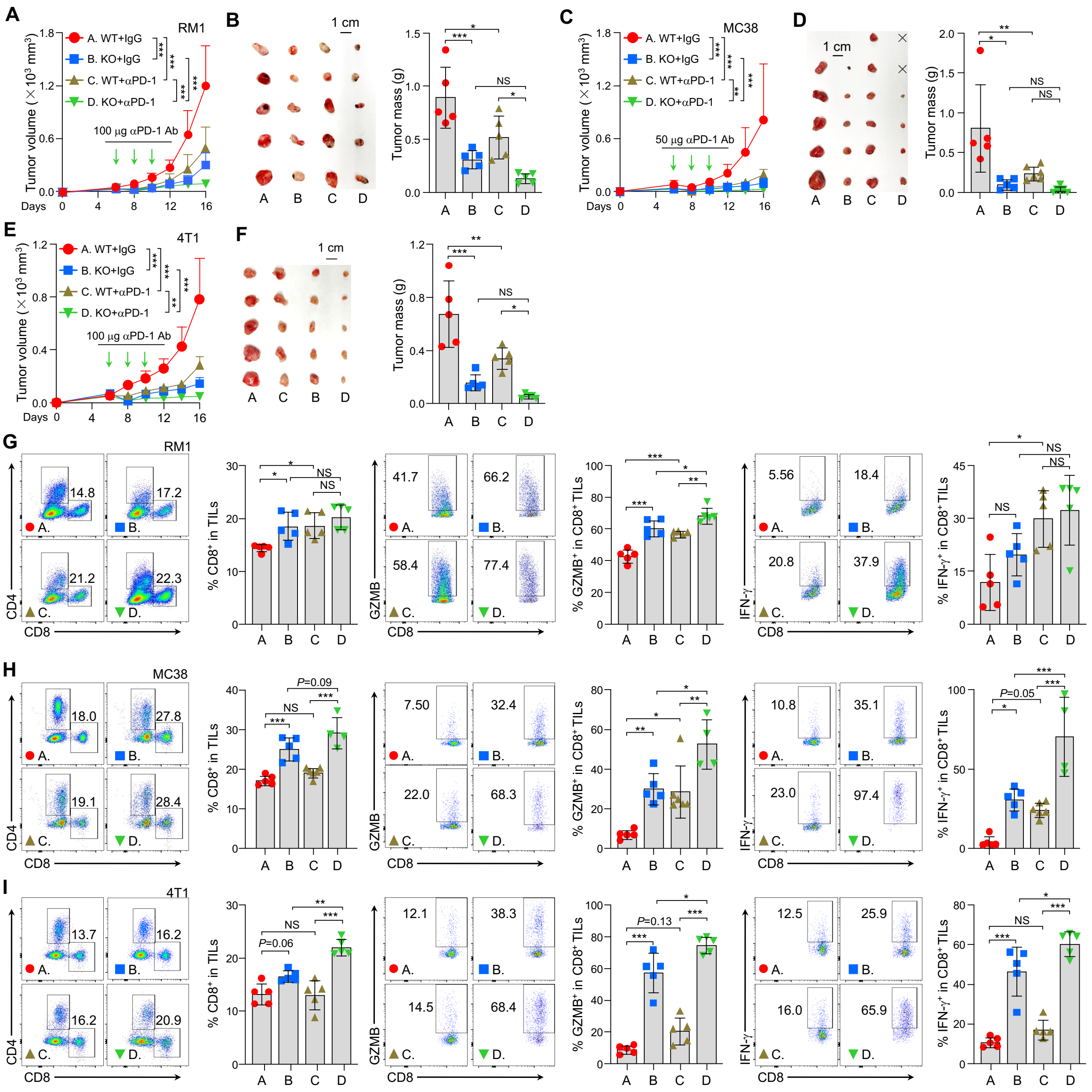

Supplementary Figure 5. Targeting *Usp22* sensitizes to αPD-1 therapy. (A and B) Tumor growth (A) or endpoint weight (B) of RM1 tumors with or without *Usp22* depletion treated with 100 μg per mouse αPD-1 antibody or isotype antibody every other day for three doses after 6 days of tumor cell implantations when the tumors reached about 50-100 mm^3 in volume. Photograph of tumors shown. Scale bar: 1 cm. Green arrows indicate days of αPD1 antibody administration. **(C-D)** Tumor growth (C) or endpoint weight (D) of MC38 tumors with or without *Usp22* depletion treated with 50 μg per mouse αPD-1 antibody or isotype antibody every other day after 6 days of tumor cell implantations when the tumors reached about 50-100

mm^3 in volume. Photograph of tumors shown. Scale bar: 1 cm. Green arrows indicate days of αPD1 antibody administration. **(E and F)** Tumor growth (E) or endpoint weight (F) of 4T1 tumors with or without *Usp22* depletion treated with 100 μg per mouse αPD-1 antibody or isotype antibody every other day after 6 days of tumor cell implantations when the tumors reached about 50-100 mm^3 in volume. Photograph of tumors shown. Scale bar: 1 cm. Green arrows indicate days of αPD1 antibody administration. **(G-I)** Flow cytometric analysis of tumor-infiltrating $CD8^+$ T cells and GMZB or INF-γ producing $CD8^+$ T cells from RM1 (G), MC38 (H), or 4T1 (I) tumors. Quantification and representative images of flow cytometric analysis are shown. Data are presented as the Mean ± SD. NS indicates no significance. $^*P< 0.05$, $^{**}P< 0.01$, and $^{***}P< 0.001$. Statistics were calculated by unpaired two-way ANOVA with multiple comparisons (A, C, E), one-way ANOVA followed by Tukey's test (B, D, F-I).

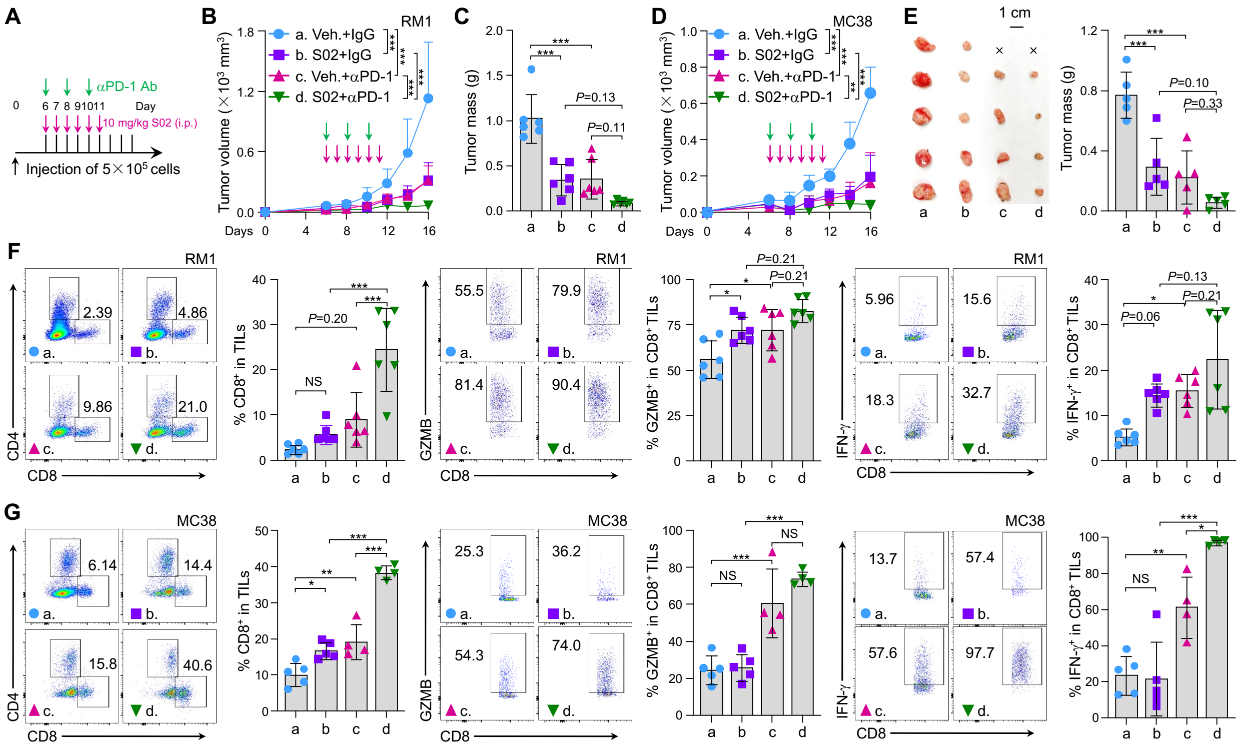

Supplementary Figure 6. Small molecular inhibitor of USP22 improves αPD-1 therapy efficacy. (A) Schematic showing the treatment of S02 alone or combined with mouse α-PD1 blocking antibody. Purple and green arrows indicate administration of 10 mg/kg S02 alone or combined with 100 mg (RM1) or 50 mg (MC38) α-PD1 blocking antibody. Black arrows indicate days of 5×10^5 RM1 or MC38 cells injection. **(B-C)** Tumor growth (B) or weight (C) of from RM1 tumor bearing mice treated with 10 mg/kg S02 alone or combined with 100 μg αPD1 blocking antibody on indicated days. *P* value was calculated by two-way ANOVA with multiple comparisons. **(D-E)** Tumor growth (D) or weight (E) of from MC38 tumor bearing mice treated with 10 mg/kg S02 alone or combined with 50 μg αPD1 blocking antibody. *P* value was calculated by two-way ANOVA with multiple comparisons. **(F-G)** Flow cytometric analysis of percentages of tumor-infiltrating CD8$^+$ T cells among the total CD45$^+$ lymphocytes or GZMB producing CD8$^+$ T cells from RM1 (F) or MC38 (G) tumor bearing mice administered with S02 alone or combined with αPD1 blocking antibody. Quantification and representative images of flow cytometric analysis are shown. Data are presented as the Mean ± SD. NS indicates no significance. *P< 0.05, **P< 0.01, and ***P< 0.001. Statistics were calculated by unpaired two-way ANOVA with multiple comparisons (B, D), one-way ANOVA followed by Tukey's test (C, E-G).

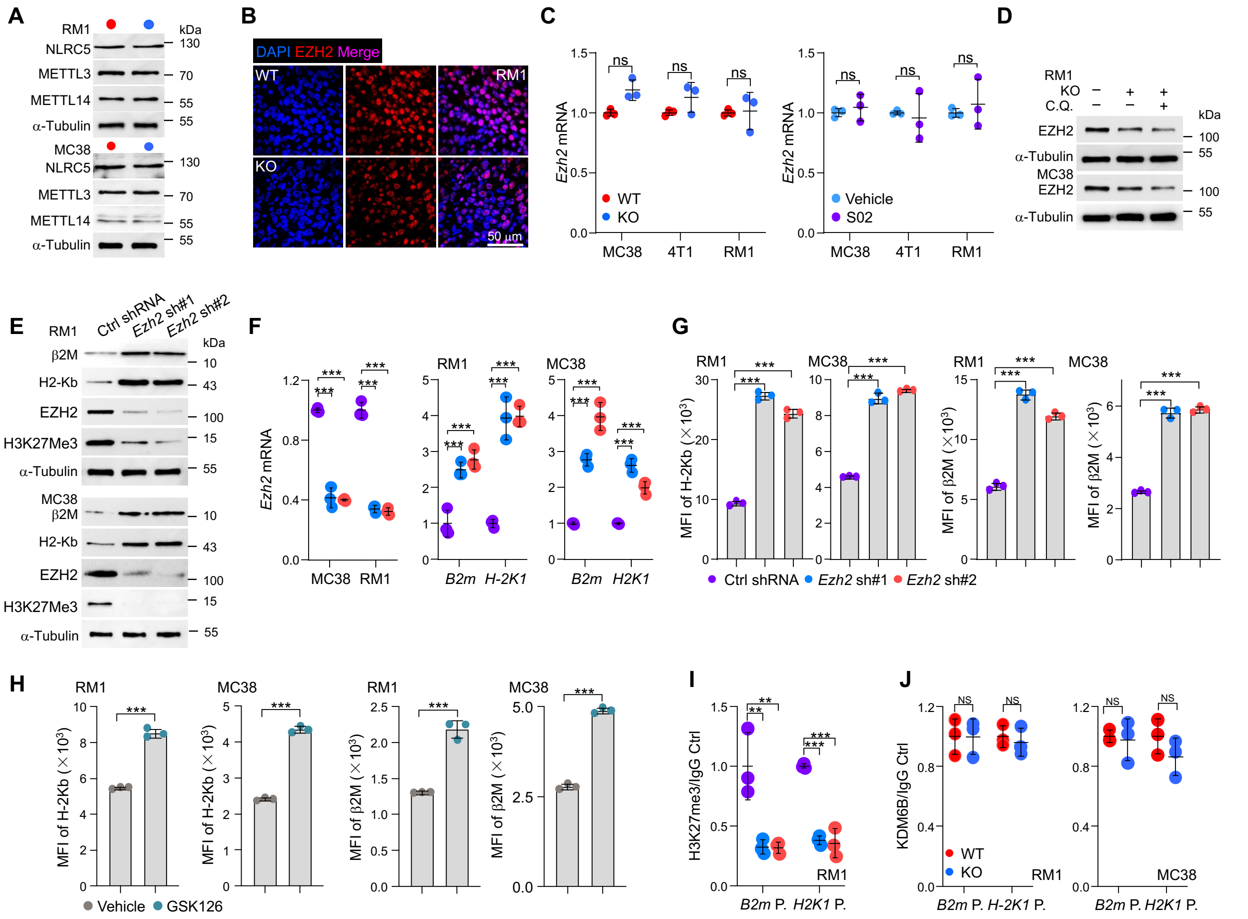

Supplementary Figure 7. USP22 interacts with and stabilizes EZH2. (A) Immunoblot analysis of indicated proteins expression in RM1 or MC38 cells with or without *Usp22* depletion. **(B)** Representative images of immunofluorescence staining and quantification of EZH2 in slides from WT or *Usp22* KO tumors. Immunoblot analysis of indicated proteins expression in RM1 or MC38 cells with or without *Ezh2* genetic inhibition determined by RT-PCR. **(C)** The mRNA expression of *Ezh2* in RM1 or MC38 WT or *Usp22* KO cells (Left) or pharmacological (Right) inhibition were determined by RT-PCR. Indicated genes expression were normalized to *Actb* expression. **(D)** Immunoblotting analysis of indicated proteins expression in RM1 or MC38 WT or *Usp22* KO cells treated with or without or chloroquine (50 μM, 12 hours). C.Q. indicates chloroquine. α-Tubulin was used as internal control. **(E)** Immunoblot analysis of indicated proteins expression in RM1 or MC38 cells with or without *Ezh2* genetic inhibition. **(F-G)** Effects of *Ezh2* genetic inhibition on mRNA (F) or cell surface level (G) of H2-Kb or β2M expression on RM1 or MC38 cells. Quantification showing that H2-Kb or β2M mRNA and cell surface level were increased upon on *Ezh2*

inhibition. **(H)** RM1 or MC38 cells were treated with 10 μM GSK126 (EZH2 inhibitor) for 24 hours. Cell surface level of H2-Kb or β2M on RM1 or MC38 cells treated with or without GSK126 were determined by flow cytometry. **(I)** Ch-IP and subsequent qRT-PCR analysis of H3K27me3 enrichment in selected promoter regions of *B2m* or *H-2K1* genes in *Ezh2* inhibition or control cells. H3K27me3 indicates trimethylation of lysine 27 on histone H3 protein. **(J)** Ch-IP and subsequent qRT-PCR analysis of KDM6B enrichment in selected promoter regions of *B2m* or *H-2K1* genes in RM1 *Usp22* inhibition or control cells. NS indicates no significance. Data are presented as the Mean ± SD. *$P < 0.05$, **$P < 0.01$, and ***$P < 0.001$. Statistics were calculated by unpaired two-tailed t-test (C, H, J) or one-way ANOVA followed by Dunnett's test (F, G, I).

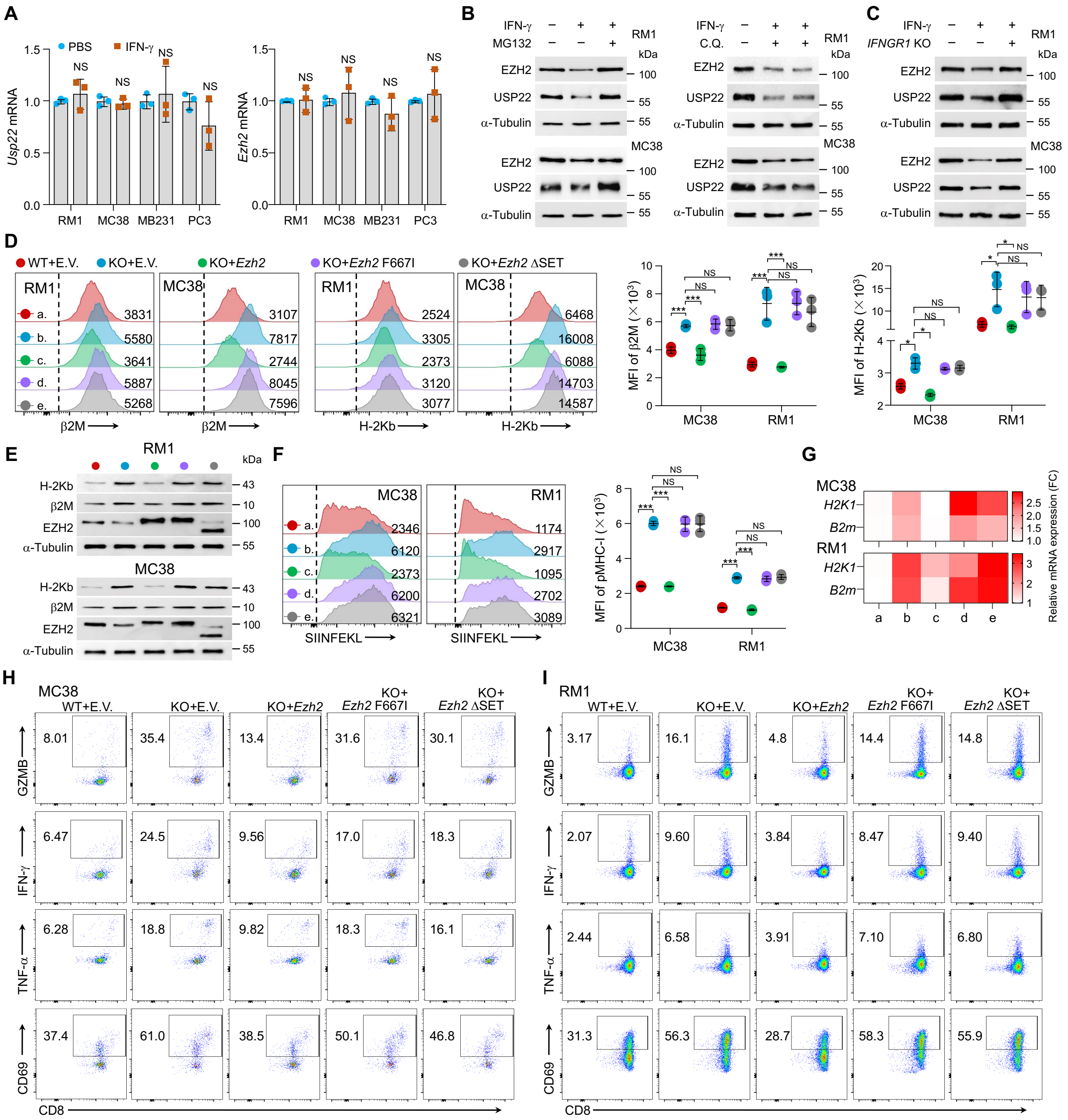

Supplementary Figure 8. *Ezh2* compromises anti-tumor immunity impaired by *Usp22* loss. (A) Relative mRNA expression of *Usp22* and *Ezh2* in indicated tumor cells treated with 10 ng/mL IFN-γ or PBS for 48 hours. *Usp22* and *Ezh2* mRNA expressions were normalized to the mean level in indicated cells treated with PBS. **(B)** Immunoblot analysis of indicated proteins treated with 10 μM MG132 or 50 μM chloroquine for 12 hours. **(C)** Immunoblot analysis of indicated proteins treated with 10 ng/mL IFN-γ or PBS for 48 hours. **(D)** Cell-surface expression of β2M or H-2Kb in *Usp22*-deficient RM1 and MC38 cells with or

without *Ezh2*, *Ezh2* F667I or ΔSET mutant reconstitution. Quantification showing that *Ezh2* compromises the promotion effects of *Usp22* depletion on the surface β2M and H-2Kb expression. E.V. indicates empty vector. Representative flow cytometric plots of β2M or H-2Kb on indicated cell surface are shown. **(E)** Representative images of immune blot analysis of indicated proteins in *Usp22*-deficient RM1 or MC38 cells with or without *Ezh2*, *Ezh2* F667I or ΔSET mutant reconstitution. **(F)** Cell-surface levels of pMHC-I in *Usp22*-deficient RM1 and MC38 cells with or without *Ezh2*, *Ezh2* F667I or ΔSET mutant reconstitution. Ectopic expression of *Ezh2*, but not *Ezh2* F667I or ΔSET mutant, blocked the enhanced neoantigen presentation capacity by *Usp22* depletion. Representative flow cytometric plots of pMHC-I on indicated cell surface and quantification are shown. **(G)** Relative mRNA expression of *B2m* and *H2K1* in *Usp22*-deficient RM1 and MC38 cells with or without *Ezh2*, *Ezh2* F667I or ΔSET mutant reconstitution. *B2m* and *H2K1* mRNA expressions were normalized to the mean level in the WT cells. **(H-I)** Naïve OT-I $CD8^+$ T cells were isolated from OT-I mice and then co-cultured with *Usp22*-deficient MC38 (G) or RM1 (H) OVA expressing cells with or without *Ezh2*, *Ezh2* F667I or ΔSET mutant reconstitution for 8 hours at the ratio of 1:1 (cancer cells to T cells ratio) in the presence of CD28 blocking antibodies treatment. Representative images of flow cytometric analysis of percentages of $GZMB^+$, IFN-γ^+ and TNF-α^+ producing $CD8^+$ T cells followed by co-culturing with indicated RM1 and MC38 OVA expressing cells. Quantification data are shown in Figure 4F. Data are presented as the Mean ± SD. *P< 0.05, **P< 0.01, and ***P< 0.001. Statistics were calculated by unpaired two-tailed t-test (A) or one-way ANOVA followed by Tukey's test (D, F).

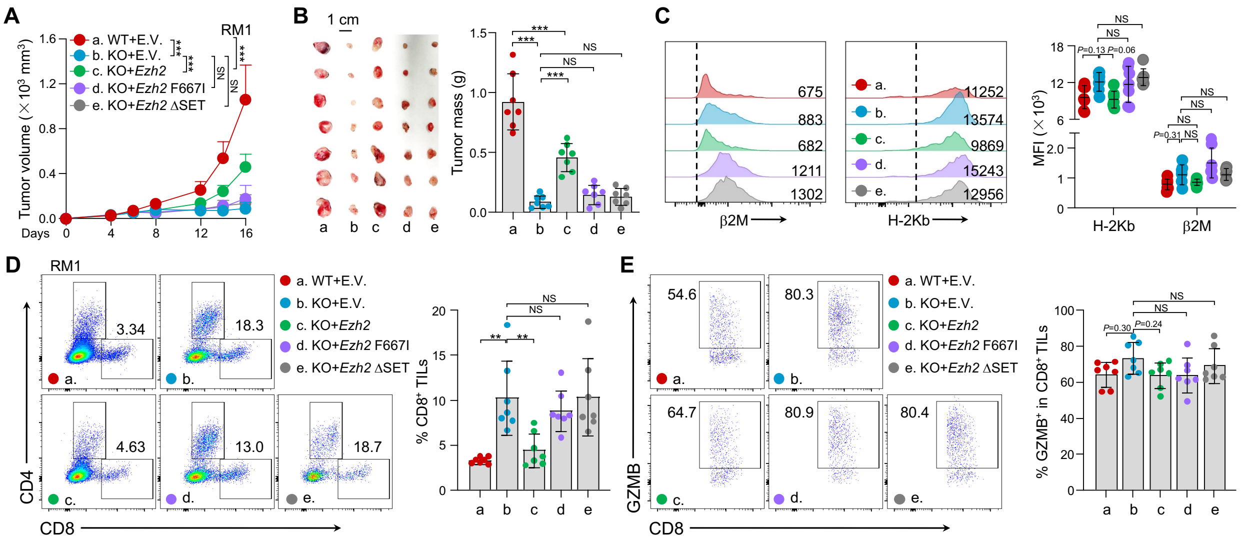

Supplementary Figure 9. *Ezh2* compromises anti-tumor immunity impaired by *Usp22* loss. (A-B) A total of 5×10^5 RM1 cells with *Ezh2*, *Ezh2* F667I, or ΔSET mutant reconstitution in the setting of *Usp22* depletion were subcutaneously inoculated into immunocompetent mice. Tumor volume (A) and endpoint mass (B) of indicated tumors were recorded. *P* value was calculated by two-way ANOVA with multiple comparisons. **(C)** The expression of β2M and H-2Kb on indicated tumor cell surface. The representative flow cytometric plots and quantification are shown. **(D-E)** The frequencies of tumor-infiltrating $CD8^+$ T cells (D) or $GZMB^+$ producing $CD8^+$ T cells (E) from indicated RM1 tumors collected 16 days after tumor cells inoculation. The representative flow cytometric plots and quantification are shown. Data are presented as the Mean ± SD. NS indicates no significance. *$P < 0.05$, **$P < 0.01$, and ***$P < 0.001$. Statistics were calculated by unpaired two-way ANOVA with multiple comparisons (A), one-way ANOVA followed by Tukey's test (B-E).

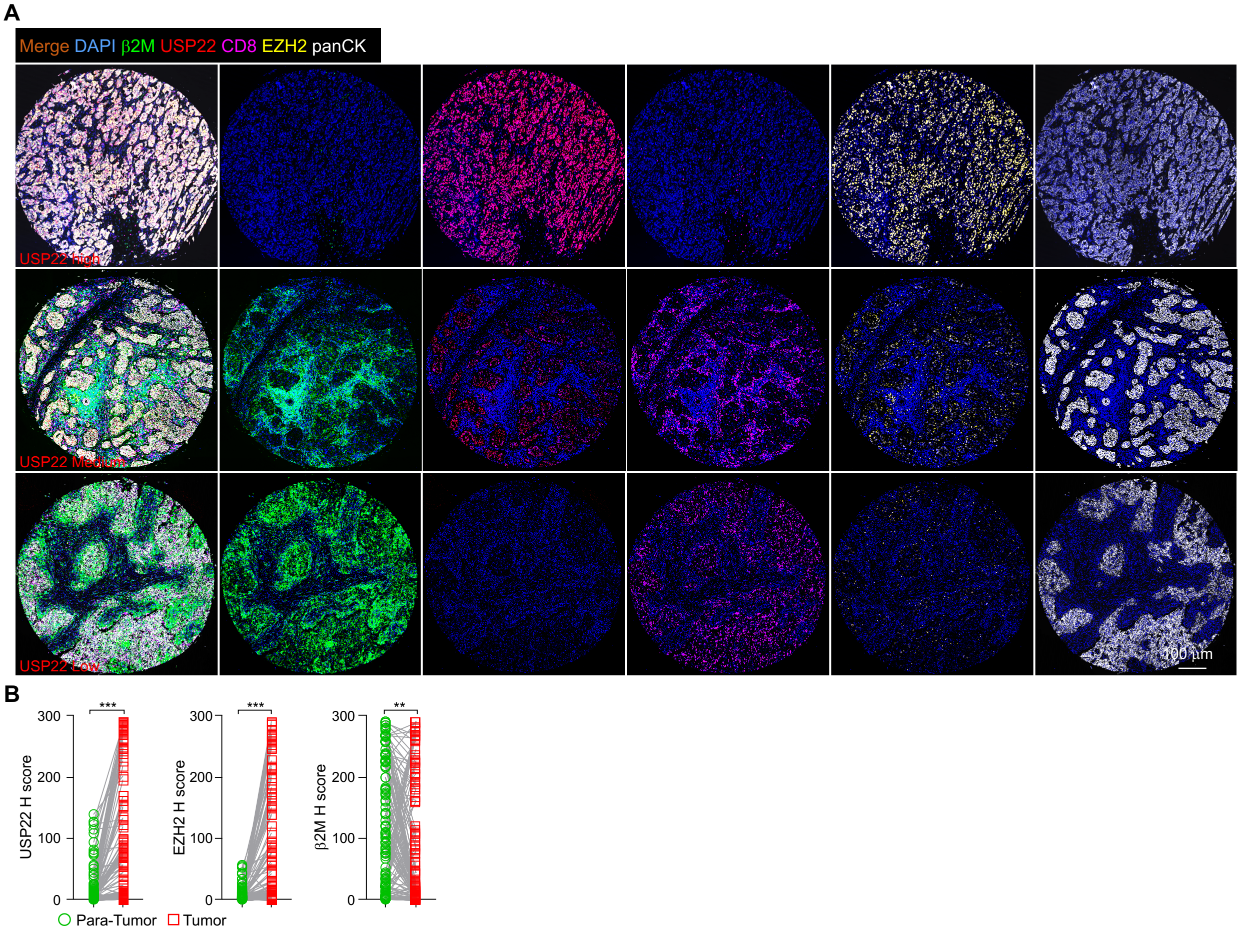

Supplementary Figure 10. USP22 is highly expressed on breast cancer cells with low β2M expression. (A) Whole picture of breast cancer tissue of multiplexed immunofluorescence staining shown in Figure 5A for tumoral and para-tumoral USP22, EZH2, β2M expression and the ratio of CD8 T cells infiltration. **(B)** Quantification data para-tumoral and tumoral USP22, EZH2, β2M in breast cancer in each case. Data are presented as the Mean ± SD. NS indicates no significance. $^{**}P < 0.01$, and $^{***}P < 0.001$. Statistics were calculated by unpaired two-tailed t-test (B).

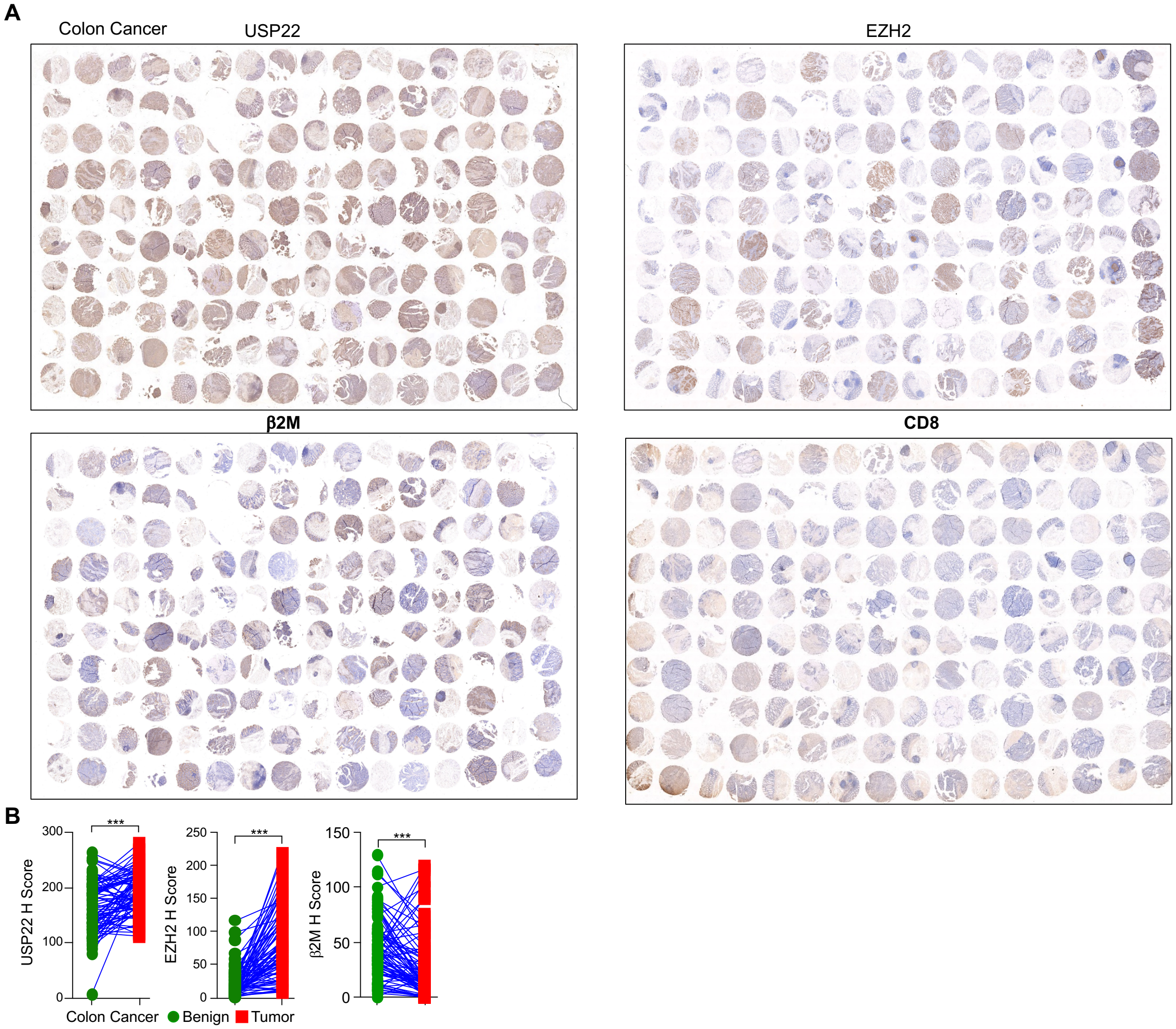

Supplementary Figure 11. USP22 is highly expressed in colon cancer. (A) Whole picture of colon tissue microarray of immunohistochemical staining for USP22, EZH2, β2M and CD8. **(B)** Quantification data of USP22, EZH2, β2M in colon cancer or benign tissues. Data are presented as the Mean ± SD. NS indicates no significance. ***$P < 0.001$. Statistics were calculated by unpaired two-tailed t-test (B).

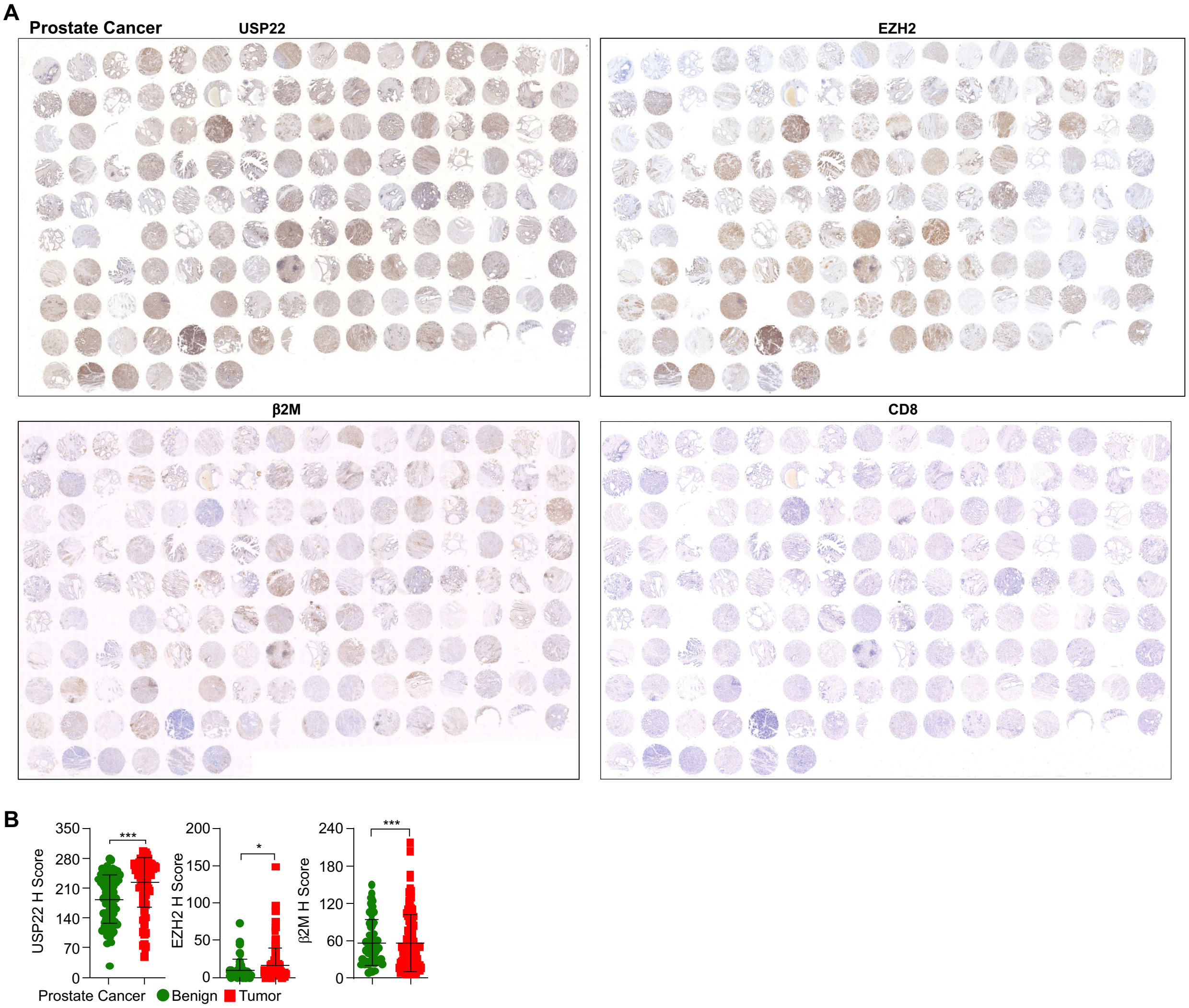

Supplementary Figure 12. USP22 is highly expressed in prostate cancer. (A) Whole picture of prostate tissue microarray of immunohistochemical staining for USP22, EZH2, β2M and CD8. **(B)** Quantification data of USP22, EZH2, β2M in prostate cancer or benign tissues. Data are presented as the Mean ± SD. NS indicates no significance. **P*< 0.05, and ****P*< 0.001. Statistics were calculated by unpaired two-tailed t-test (B).

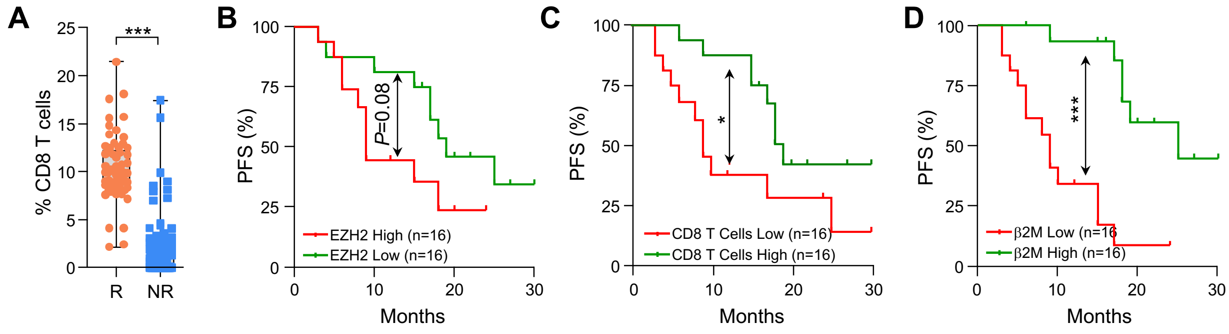

Supplementary Figure 13. USP22 expression links with ICB resistance. (A) Quantification data of percent CD8⁺ T cells among total cells from pretreatment biopsies from responders or non-responders. R. and NR. indicate responders and non-responders, respectively. **(B)** Kaplan-Meier plot of progression-free survival for patients treated with αPD-1 in EZH2 versus high group. Patients were classified into the EZH2 low or high groups, with the median expression value of all the samples used as the cutoff. PFS indicates progression-free survival. **(C)** Kaplan-Meier plot of progression-free survival for patients treated with αPD-1 in CD8⁺ T cells infiltration frequencies low versus high group. Patients were classified into the CD8⁺ T cells infiltration frequencies low or high groups, with the median expression value of all the samples used as the cutoff. **(D)** Kaplan-Meier plot of progression-free survival for patients treated with αPD-1 in β2M low versus high group. Patients were classified into the β2M low or high groups, with the median expression value of all the samples used as the cutoff. Data are presented as the Mean ± SD. NS indicates no significance. **P*< 0.05, and ****P*< 0.001. Statistics were calculated by unpaired two-tailed t-test (A), Log rank t test (C-E).

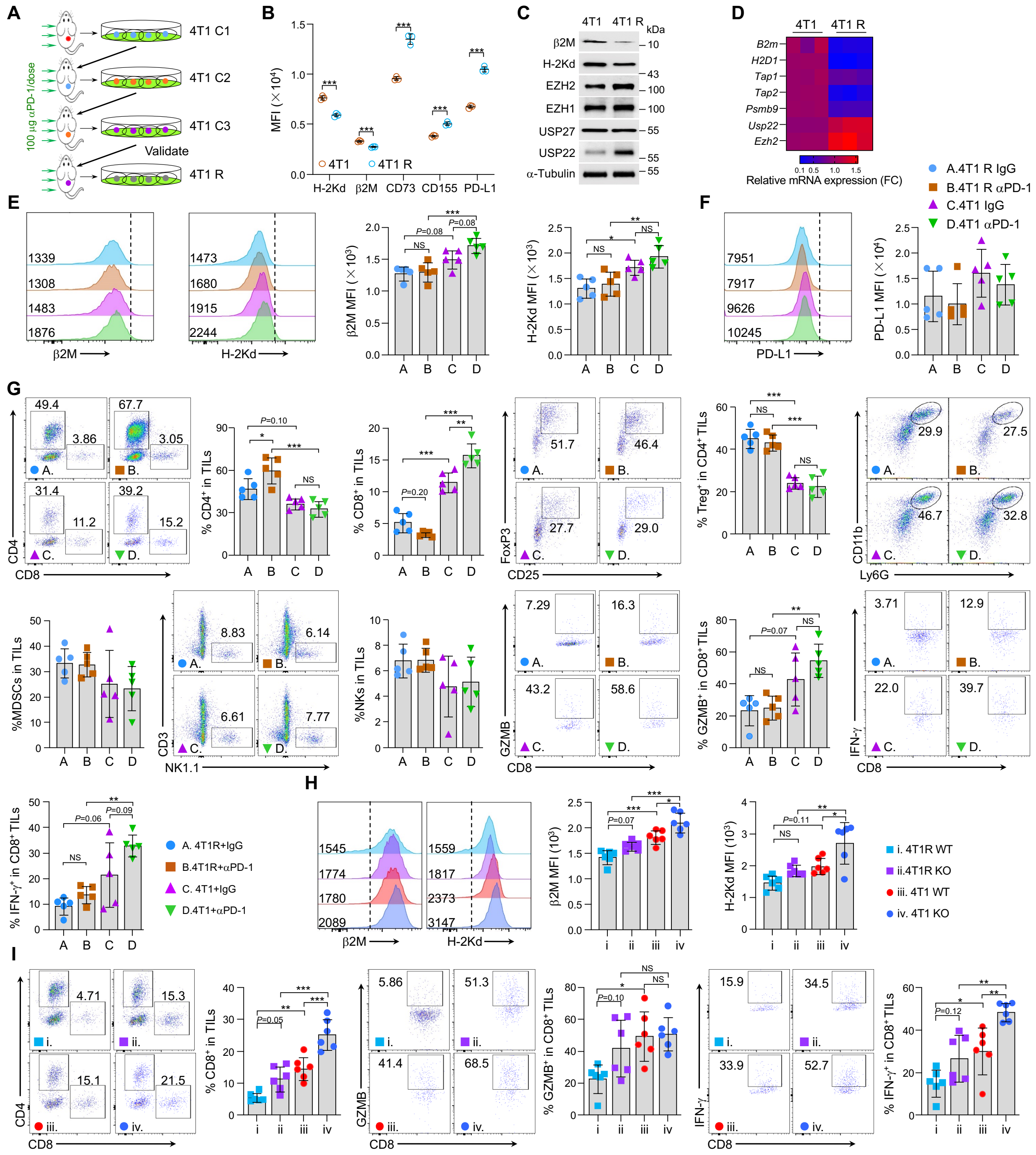

Supplementary Figure 14. Establishment of *in vivo* ICB resistance mice model. (A) A total of 5 × 10^5 4T1 were orthotopically inoculated into the mammary fat pad (MFP) of female BALB/c mice. After 6 days, mice were administered with 100 μg αPD-1 blocking antibodies. After 18 days of injection 4T1 cells, tumors were harvested and CD45$^-$ tumor cells were cultured and subsequently orthotopically inoculated into MFP

of female BALB/c mice. After 6 days, mice were administered with three doses 100 μg αPD-1 antibodies. After 16 days of injection the first passage of 4T1 C1 (4T1 C1) cells, tumors were harvested, and tumor cells were cultured and subsequently orthotopically inoculated into MFP of female BALB/c mice again. A total of 5×10^5 4T1 cells or second passage of 4T1 cells (4T1 C2) were orthotopically inoculated into MFP of BALB/c mice and then treated with three doses 100 μg αPD-1 antibodies. After three cycles of serial transplantation, 4T1 C3 tumors were harvested in days 16 and subjected to following studies. Green arrows indicate mice were injected with 100 μg αPD-1 antibodies. **(B)** Cell surface expression of indicated proteins in 4T1 or 4T1R cells and quantification are shown. **(C)** Immunoblotting analysis of indicated proteins in 4T1 or 4T1R cells. **(D)** Heatmap summarizing for the RT-PCR results of indicated mRNA expression in 4T1 or 4T1R cells. β-actin was used as internal control. **(E)** Tumoral cell surface expression of H-2Kd or β2M in 4T1R or 4T1 tumor bearing mice treated with or without αPD-1 blocking antibodies. The quantification and images of representative flow cytometric analysis are shown. **(F)** Tumoral cell surface PD-L1 expression in 4T1R or 4T1 tumor bearing mice treated with or without αPD-1 blocking antibodies. The quantification and images of representative flow cytometric analysis are shown. **(G)** Representative flow cytometric plots and quantification of indicated cells population in tumor infiltrating lymphocytes from 4T1R or 4T1 tumor bearing mice treated with or without αPD-1 blocking antibodies. **(H)** Tumoral cell surface expression of H-2Kd or β2M in 4T1R or 4T1 tumors with or without *Usp22* deficiency. The images of representative flow cytometric analysis and quantification are shown. **(I)** Representative flow cytometric plots and quantification of tumor infiltrating $CD8^+$ as well as $GZMB^+$ or IFN-γ^+ producing $CD8^+$ T cells in 4T1 or 4T1R tumors with or without *Usp22* deficiency. Data are presented as the Mean ± SD. *$P < 0.05$, **$P < 0.01$, and ***$P < 0.001$. Statistics were calculated by unpaired two-tailed t-test (B), one-way ANOVA followed by Tukey's test (E-I).

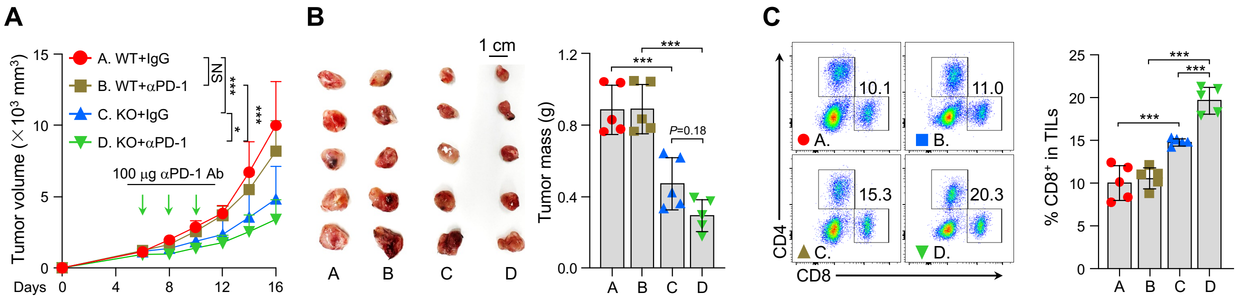

Supplementary Figure 15. Targeting *USP22* sensitizes LLC1 tumors to αPD-1 therapy. (A and B) Tumor growth (A) or endpoint weight (B) of LLC1 tumors with or without *Usp22* depletion treated with 100 μg per mouse αPD-1 antibody or isotype antibody every other day for three doses after 6 days of tumor cell implantations when the tumors reached about 50-100 mm^3 in volume. Photograph of tumors shown. Scale bar: 1 cm. Green arrows indicate days of αPD1 antibody administration. **(C)** Flow cytometric analysis of tumoral-infiltrating $CD8^+$ T cells from LLC1. Quantification and representative images of flow cytometric analysis are shown. Data are presented as the Mean ± SD. NS indicates no significance. *$P < 0.05$, **$P < 0.01$, and ***$P < 0.001$. Statistics were calculated by unpaired two-way ANOVA with multiple comparisons (A), one-way ANOVA followed by Tukey's test (B-C).

Supplementary Table 1.

Clinicopathological characteristics of patients with NSCLC in this study.

Patients NO	Gender	Cancer Type	Age	Smoking	TNM	ICB Type	PFS	Efficacy	Event	Initial therapy
Patient #1	M	LUAD	50	Yes	III A	Sintilimab	16	CR	0	NO
Patient #2	M	LUAD	64	Yes	IV A	Tislelizumab	30	CR	0	NO
Patient #3	F	LUSC	60	NO	IV A	Sintilimab	30	PR	0	NO
Patient #4	M	LUSC	69	Yes	IV	Sintilimab	4	SD	1	NO
Patient #5	M	LUSC	63	Yes	III C	Tislelizumab	24	PD	0	NO
Patient #6	M	LUSC	57	NO	IV	Tislelizumab	6	PD	1	NO
Patient #7	M	LUAD	57	Yes	IV	Camrelizumab	9	PD	1	NO
Patient #8	M	LUAD	50	Yes	IV A	Camrelizumab	9	PD	1	NO
Patient #9	M	LUSC	65	NO	III A	Sintilimab	20	CR	0	NO
Patient #10	M	LCLC	63	Yes	III B	Camrelizumab	5	PD	0	NO
Patient #11	M	LUSC	58	Yes	IV A	Camrelizumab	10	SD	1	NO
Patient #12	M	LUSC	71	Yes	IV A	Sintilimab	6	SD	0	NO
Patient #13	M	LUAD	48	Yes	III A	Camrelizumab	15	SD	1	NO
Patient #14	F	LUAD	55	NO	III B	Tislelizumab	17	SD	1	NO
Patient #15	M	LUAD	57	Yes	III A	Sintilimab	19	SD	1	NO
Patient #16	M	LUSC	62	Yes	III B	Camrelizumab	18	SD	1	NO
Patient #17	F	LUAD	64	NO	III	Camrelizumab	20	PD	0	NO
Patient #18	M	LUSC	44	NO	IV	Sintilimab	5	PD	1	NO
Patient #19	F	LUAD	49	NO	IV A	Camrelizumab	18	PR	1	NO
Patient #20	M	LUAD	59	Yes	IV B	Tislelizumab	15	PD	1	NO
Patient #21	F	LUAD	63	Yes	III C	Sintilimab	8	PD	1	NO
Patient #22	F	LUSC	61	NO	III B	Tislelizumab	9	PD	1	NO
Patient #23	M	LUSC	66	NO	III B	Tislelizumab	3	PD	1	NO
Patient #24	M	LCLC	55	Yes	III C	Sintilimab	3	PD	1	NO
Patient #25	F	LUSC	59	NO	III	Tislelizumab	17	CR	0	NO
Patient #26	F	LUAD	72	NO	III A	Sintilimab	27	PR	0	NO
Patient #27	M	LUSC	50	Yes	IV A	Camrelizumab	12	PD	0	NO
Patient #28	M	LUAD	57	Yes	IV B	Tislelizumab	6	PD	1	NO
Patient #29	F	LUAD	59	NO	III B	Sintilimab	15	PR	0	NO
Patient #30	M	LUSC	63	YES	III A	Camrelizumab	25	PR	1	NO
Patient #31	F	LUSC	65	NO	III C	Camrelizumab	22	PR	0	NO
Patient #32	F	LUAD	66	NO	IV	Sintilimab	17	PD	1	NO

Supplementary Table 2.

The sequence of Ch-IP qPCR, RT-PCR primers, or sgRNA used in this study.

Oligonucleotides of qRT-PCR Primers (5'-3')	
Mouse *Actb*_F	AGATCAAGATCATTGCTCCTCCT
Mouse *Actb*_R	ACGCAGCTCAGTAACAGTCC
Mouse *Ezh2*_F	AGCACAAGTCATCCCGTTAAAG
Mouse *Ezh2*_R	AATTCTGTTGTAAGGGCGACC
Mouse *Psmb8*_F	ATGGCGTTACTGGATCTGTGC
Mouse *Psmb8*_R	GCGGAGAAACTGTAGTGTCCC
Mouse *Psmb9*_F	GGGACAACCATCATGGCAGT
Mouse *Psmb9*_R	CAGCAGCGGAACCTGAGAG
Mouse *Psmb10*_F	GAGGAATGCGTCCTTGGAACA
Mouse *Psmb10*_R	CACAACCGAATCGTTAGTGGC
Mouse *Tap1*_F	GGACTTGCCTTGTTCCGAGAG
Mouse *Tap1*_R	CAGCATCCGACACAGCATGT
Mouse *Tap2*_F	GACATGGCTTTACTTGGGTTGC
Mouse *Tap2*_R	GTGCCCTCTATCCAGAGTCC
Mouse *H-2K1*_F	CAGGTGGAGCCCGAGTATTG
Mouse *H-2K1*_R	CGTACATCCGTTGGAACGTG
Oligonucleotides of Ch-IP qPCR Primers (5'-3')	
B2m Promoter_F	AATAAATGAAGGCGGTCCCAGG
B2m Promoter _R	TGGTGCCCTACTATCTAGGGTG
H2-K1 Promoter -F	ACTTTAAGGAAAAGCCTCTCTCTCC
H2-K1 Promoter -R	AAAGCCTCTTCCGGGAATACAA
Interg1-F	GCTCCGGGTCCTATTCTTGT
Interg1-R	TCTTGGTTTCCAGGAGATGC
Single guide RNA sequences	
Mouse sg*Usp22*	GCCATCGACCTGATGTACGG
Human sg*Usp22*	GCCATTGATCTGATGTACGG
Mouse sg*B2m*	TCACGCCACCCACCGGAGAA
Scrambled sgRNA	GTATTACTGATATTGGTGGG

Supplementary table 3. Antibodies used in this study.

REAGENT or RESOURCE	SOURCE	IDENTIFIER
**Antibodies Used in WB and IHC**		
Rabbit Anti-β2M	Abcam	Ab215889
Mouse Anti-HLA-A-B-C	Invitrogen	311410
Mouse Anti-MHC-I	Abcam	Ab281902
Rabbit Anti-USP22	Abcam	Ab195289
Rabbit Anti-α-Tubulin	Protein tech	11224-1-AP
Rabbit Anti-EZH2	CST	5246T
Rabbit Anti-EZH1	CST	42088
Mouse Anti-CD8	CST	98941T
Rabbit Anti-SUZ12	CST	3737T
Rabbit Anti-EED	CST	85322T
Rabbit Anti-H3K27me3	CST	9733T
Mouse Anti-Myc	Santa Cruz	sc-40
Mouse Anti-Flag	Sigma	1804
Mouse Anti- Flag-HRP	Sigma	A8592
Mouse Anti-UB	CST	3936s
Mouse Anti-HA HRP	Santa cruz	sc-7392
METTL3	CST	96391T
METTL14	CST	Ab98166
NLRC5	Santa Cruz	sc-515668
USP27	Thermo Fisher	PA570389
**Antibodies Used in flow cytometry**		
PE-Granzyme B	Biolegend	372208
PE-Cy7-IFN-γ	Biolegend	505826
Apc-Cy7-TNF-α	Biolegend	506343
Apc-CD8	Biolegend	100712
BV785-CD4	Biolegend	100453
FITC-CD69	Biolegend	104506
Percp-CD45	Biolegend	103130
FITC-β2M	Biolegend	316304
Apc-HLA, A, B, C	Biolegend	395711
Apc-β2M	Biolegend	395711
PE-pMHC-I	Thermo Fisher	12-5743-82
Apc-H-2Kb	Ebioscience	17-5958-82
Apc-H-2Kd	Biolegend	114714
FITC-FoxP3	Ebioscience	11-5773-82
BV510-CD3	Biolegend	100234
Apc-Cy7-NK1.1	Biolegend	1008723
PE-CD11b	Ebioscience	12-0112-81
PE-Cy7-Ly6G	Biolegend	108416
Apc-CD155	Biolegend	131510
PE-Cy7-PD-L1	Biolegend	124314
Apc-CD73	Biolegend	127209

Fixable Viability Dye eFluor 450	Ebioscience	65-0863-14
