# Supplementary material for: USP22 drives tumor immune evasion and checkpoint blockade resistance through EZH2-mediated epigenetic silencing of MHC-I
Source: J Clin Invest. 2025 Nov 18;136(1):e193162. doi: 10.1172/JCI193162 (PMC12721901; doi:10.1172/JCI193162)

Fig. 1A

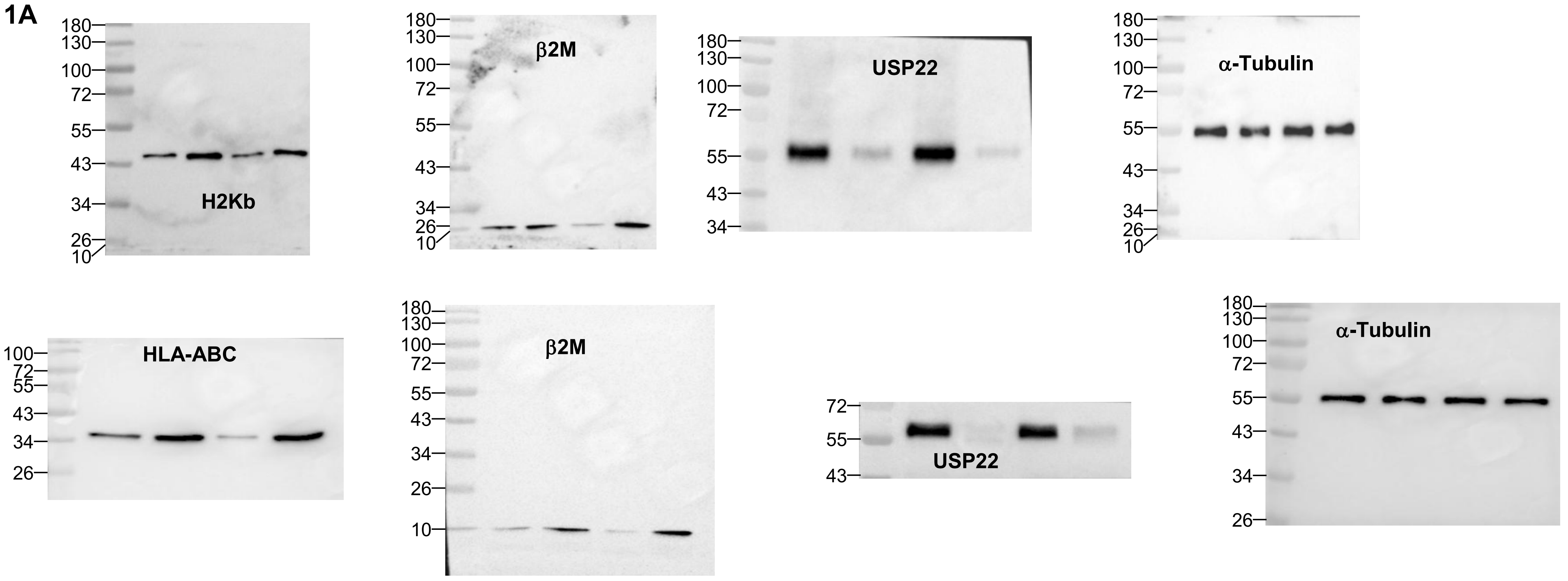

Fig. 1M

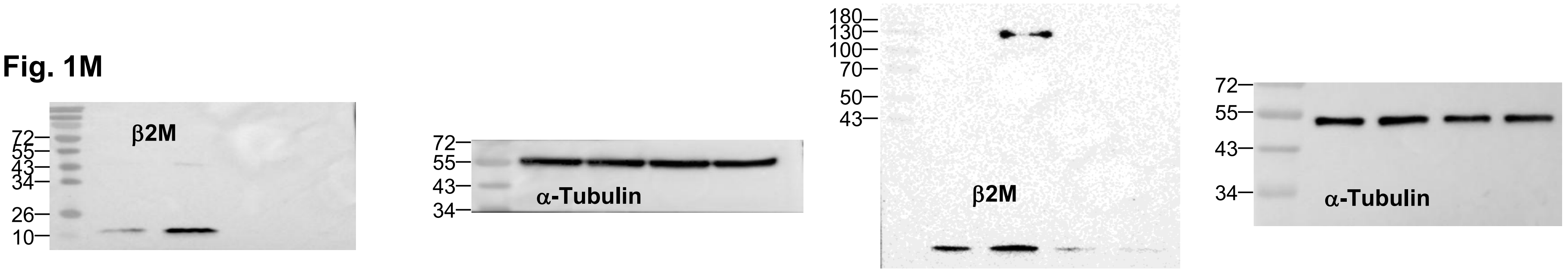

Fig. 3A

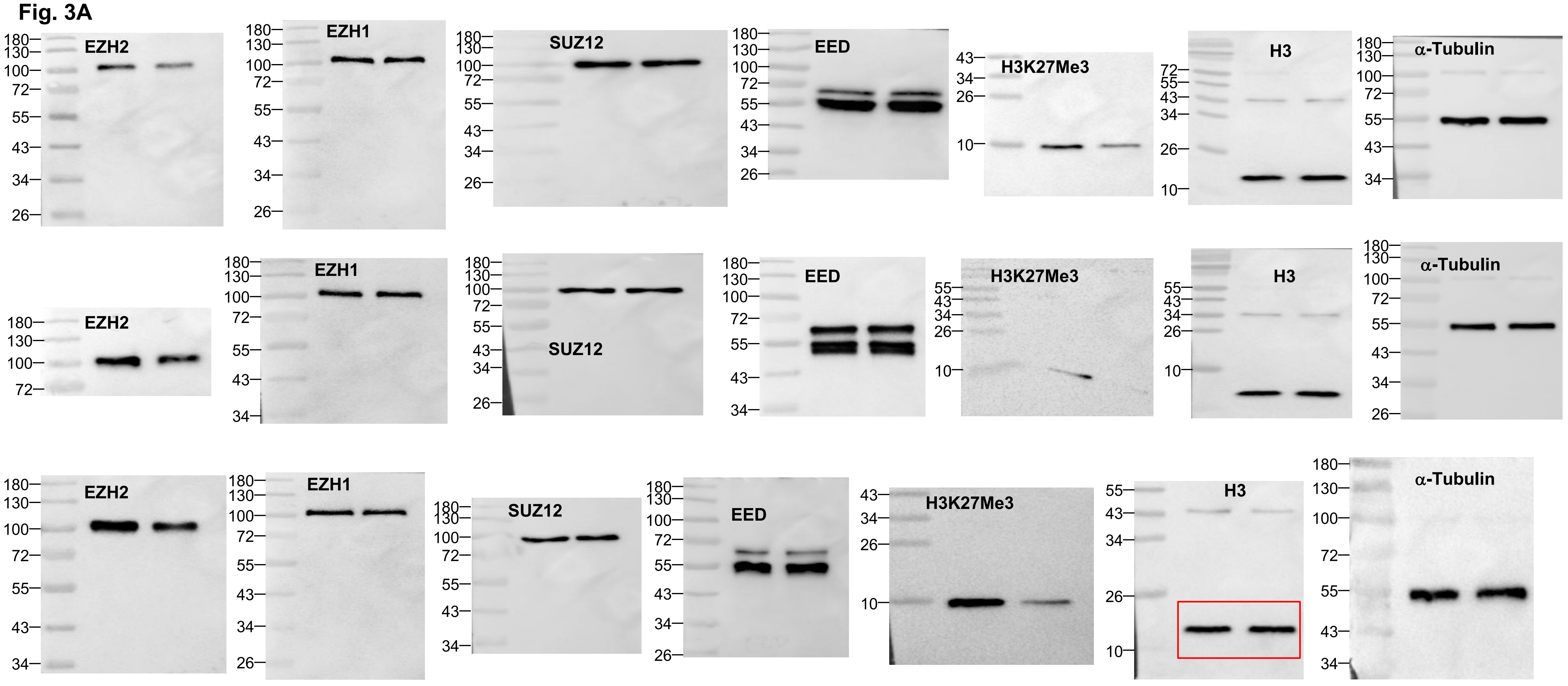

Fig. 3B

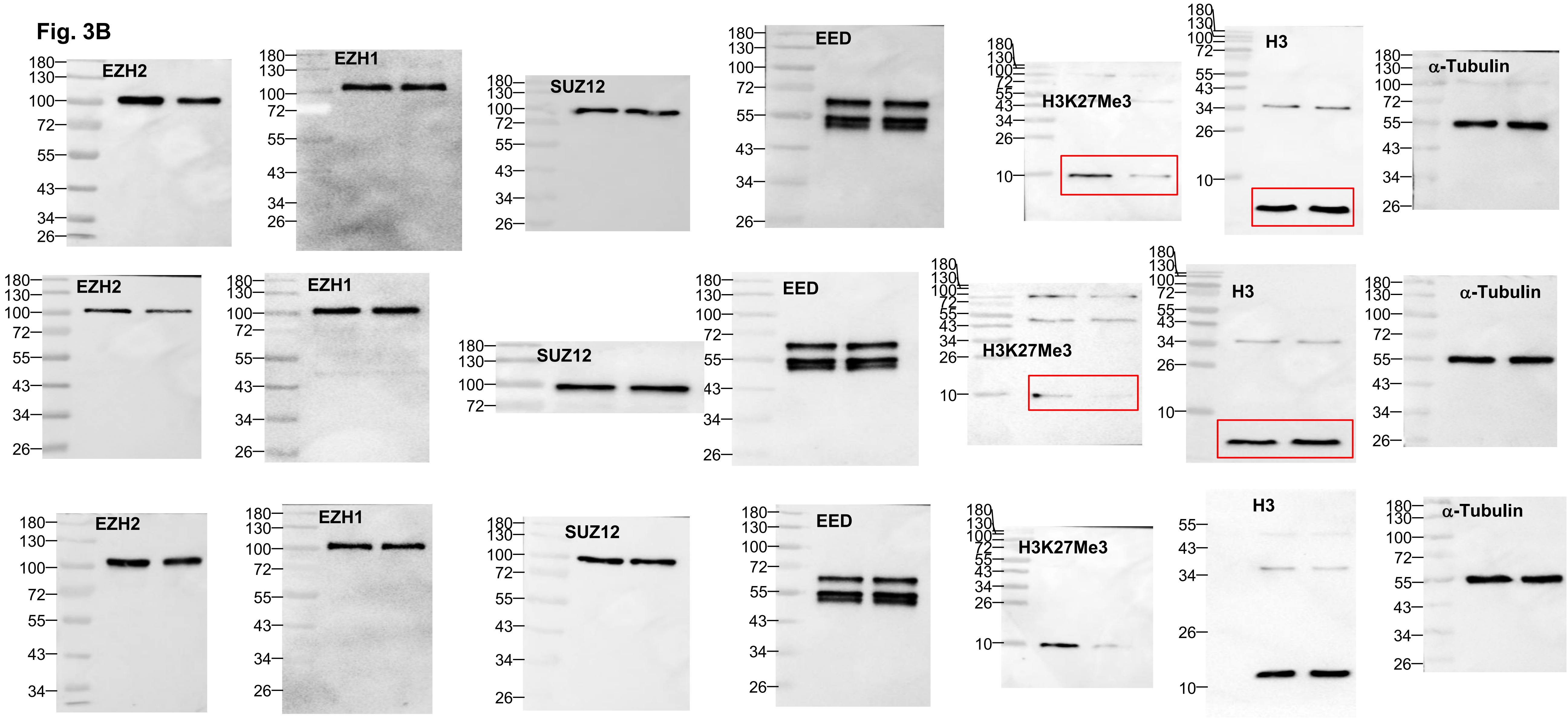

Fig. 3D

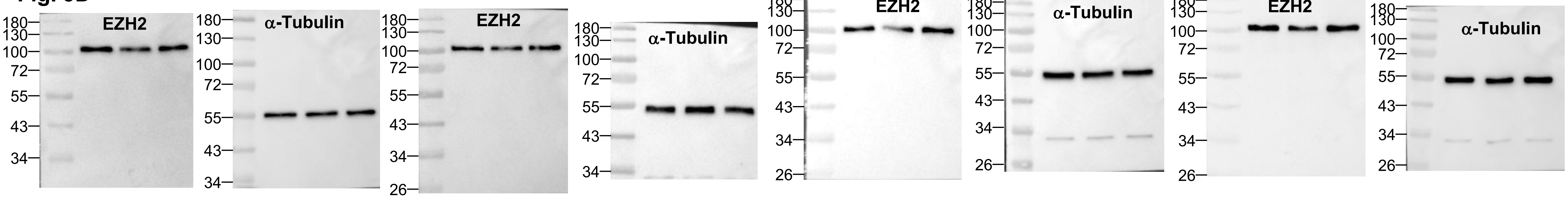

Fig. 3G

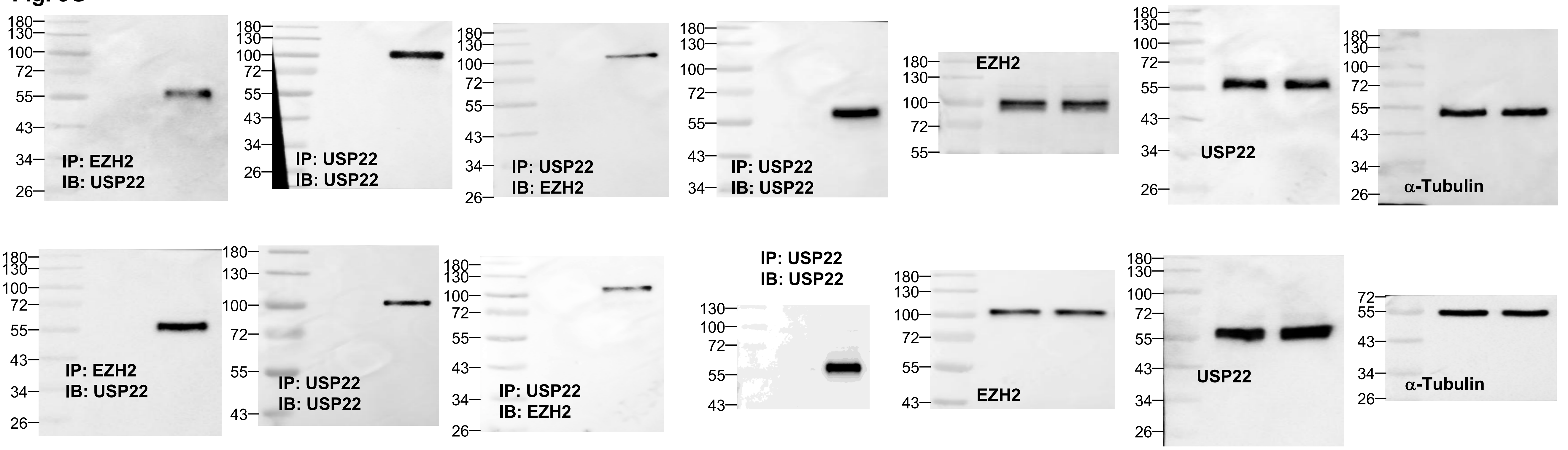

Fig. 3H

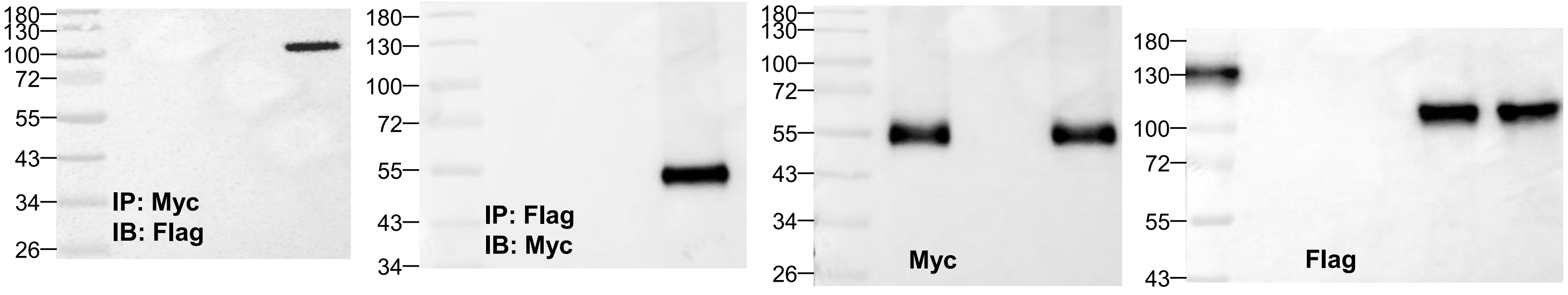

Fig. 3I

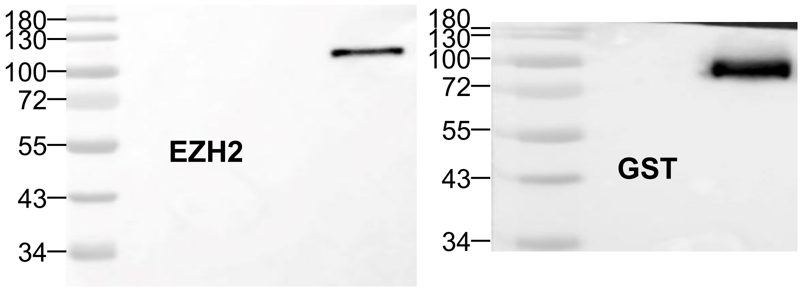

Fig.3K

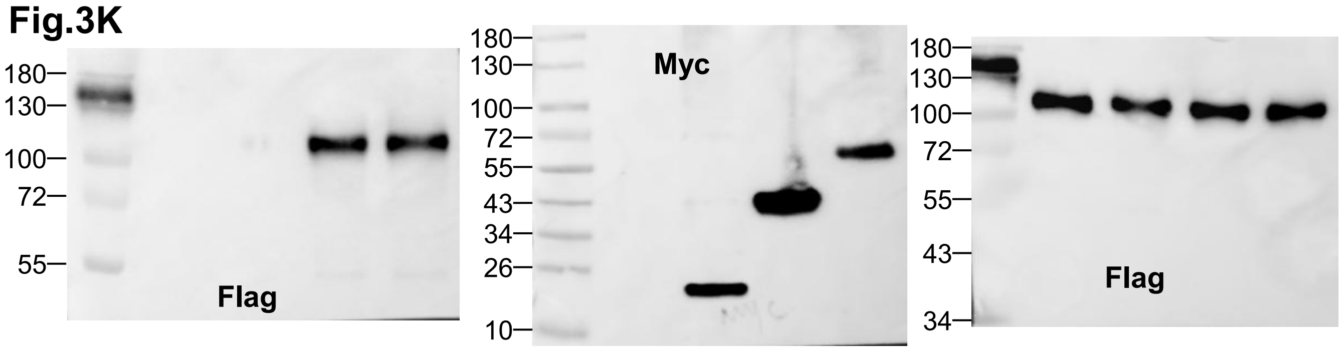

Fig.3L

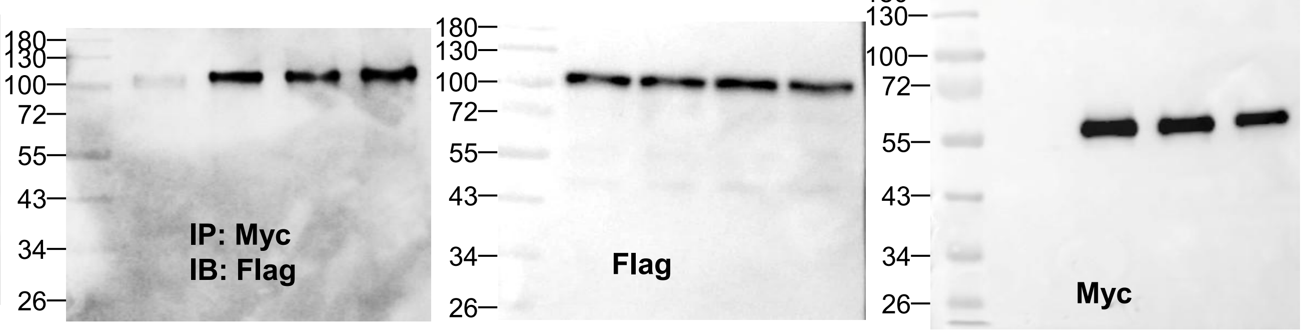

Fig.3N

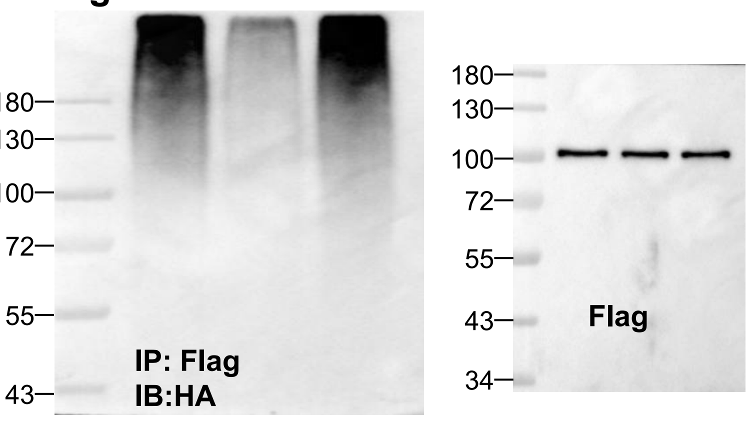

Fig.3O

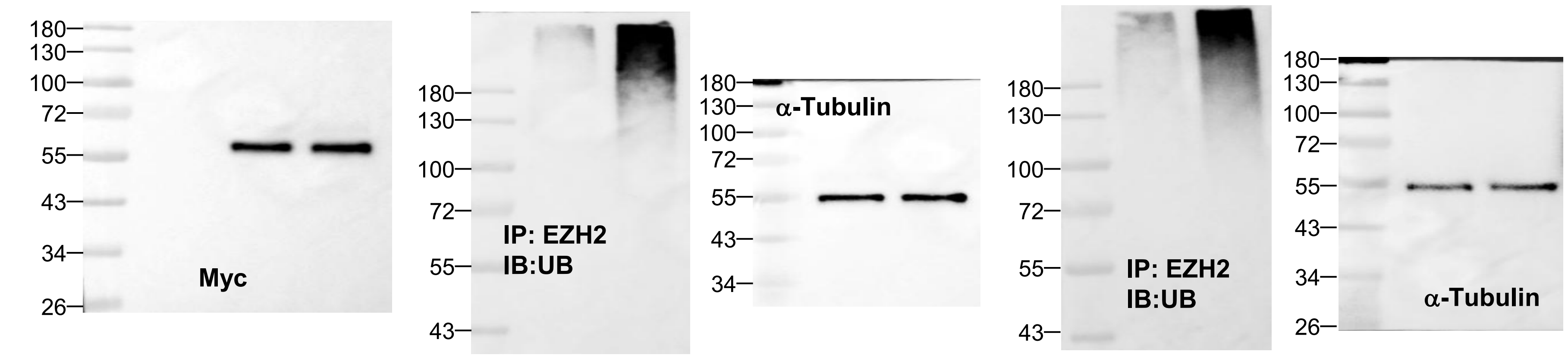

Fig.3P

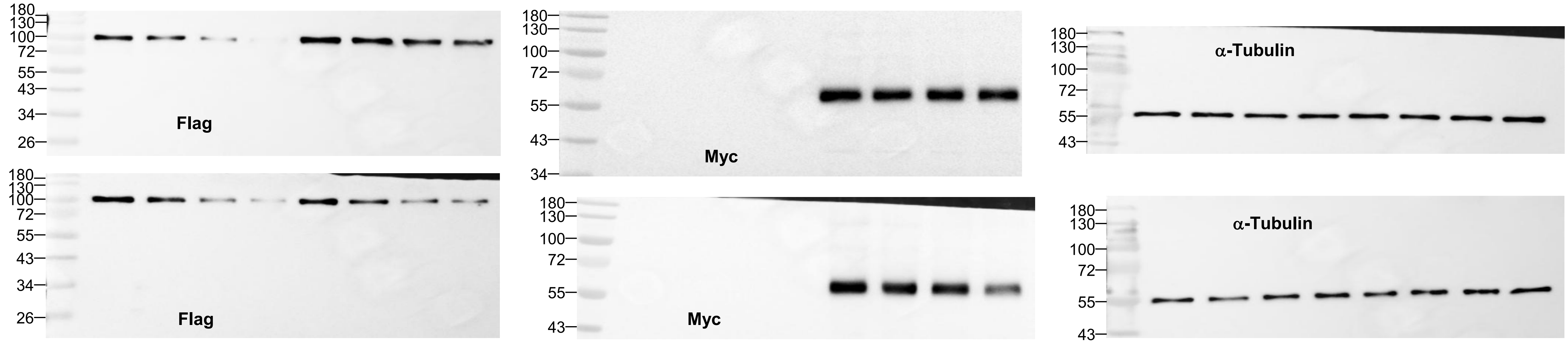

Fig.3Q

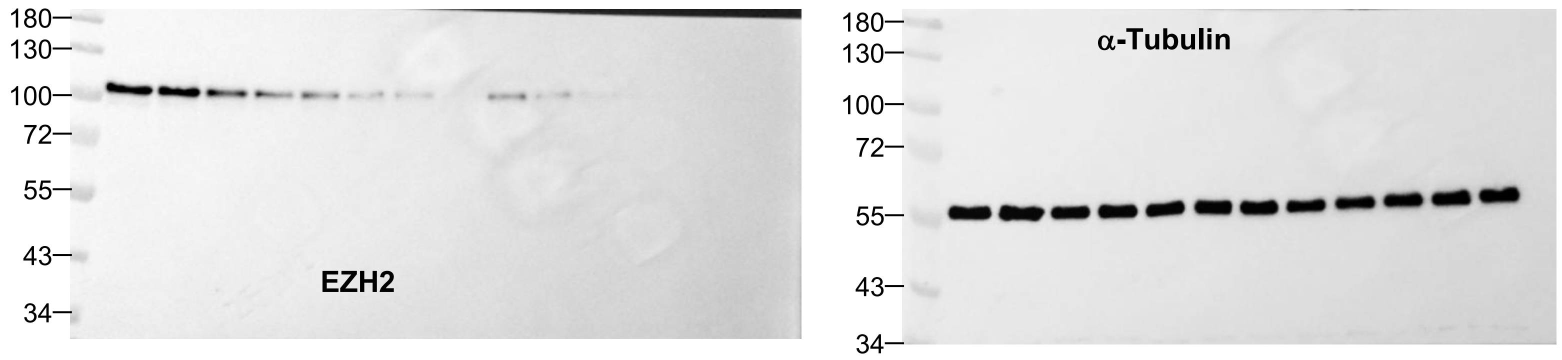

Fig.3R

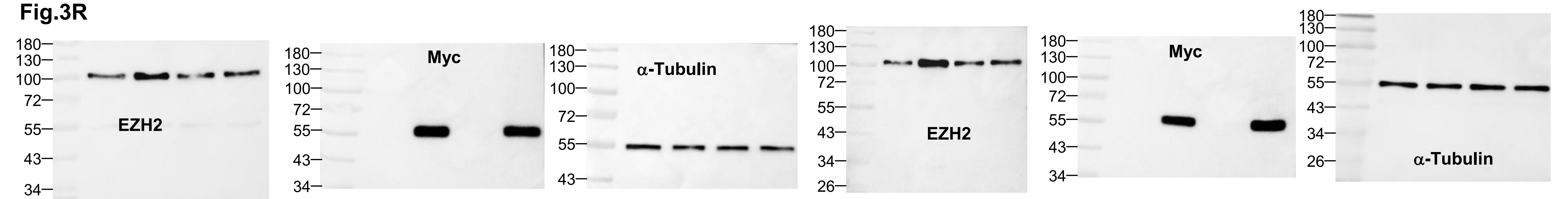

Fig.4A

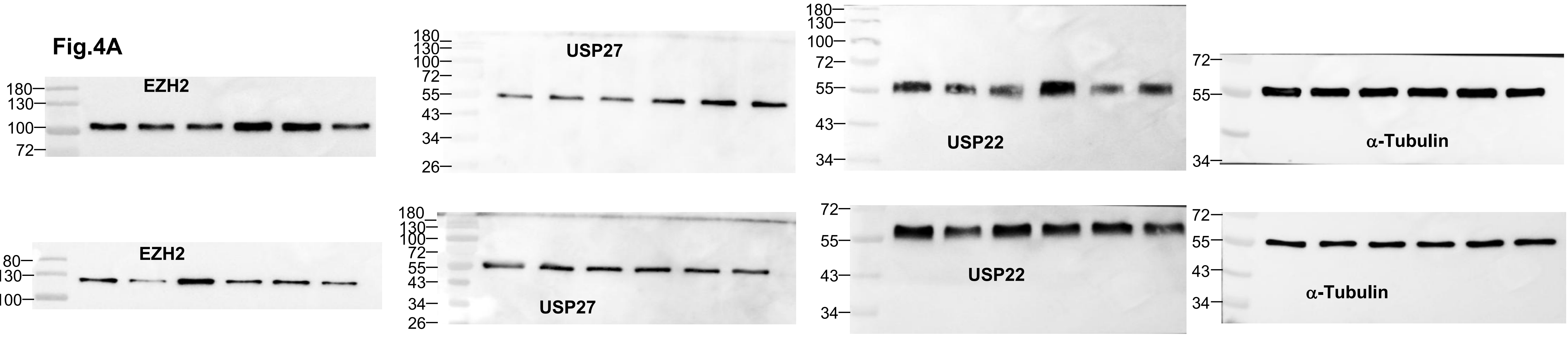

Fig.4B

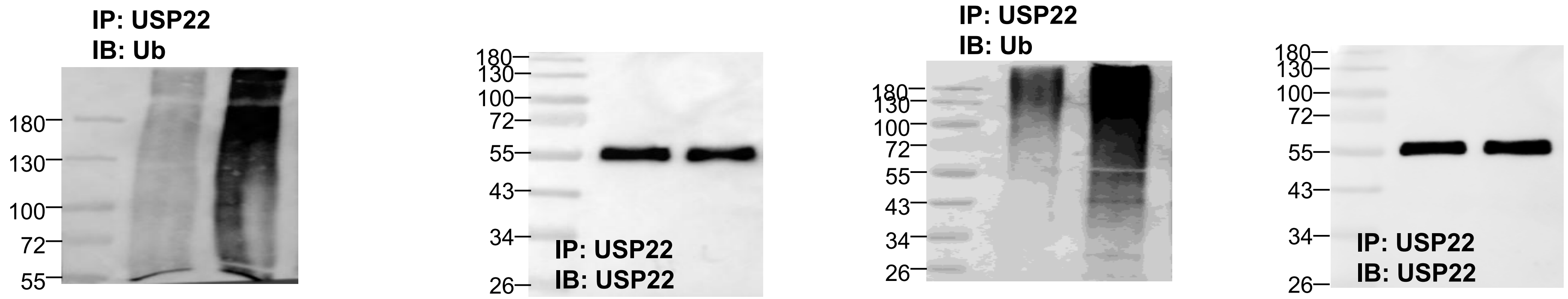

Fig.4C

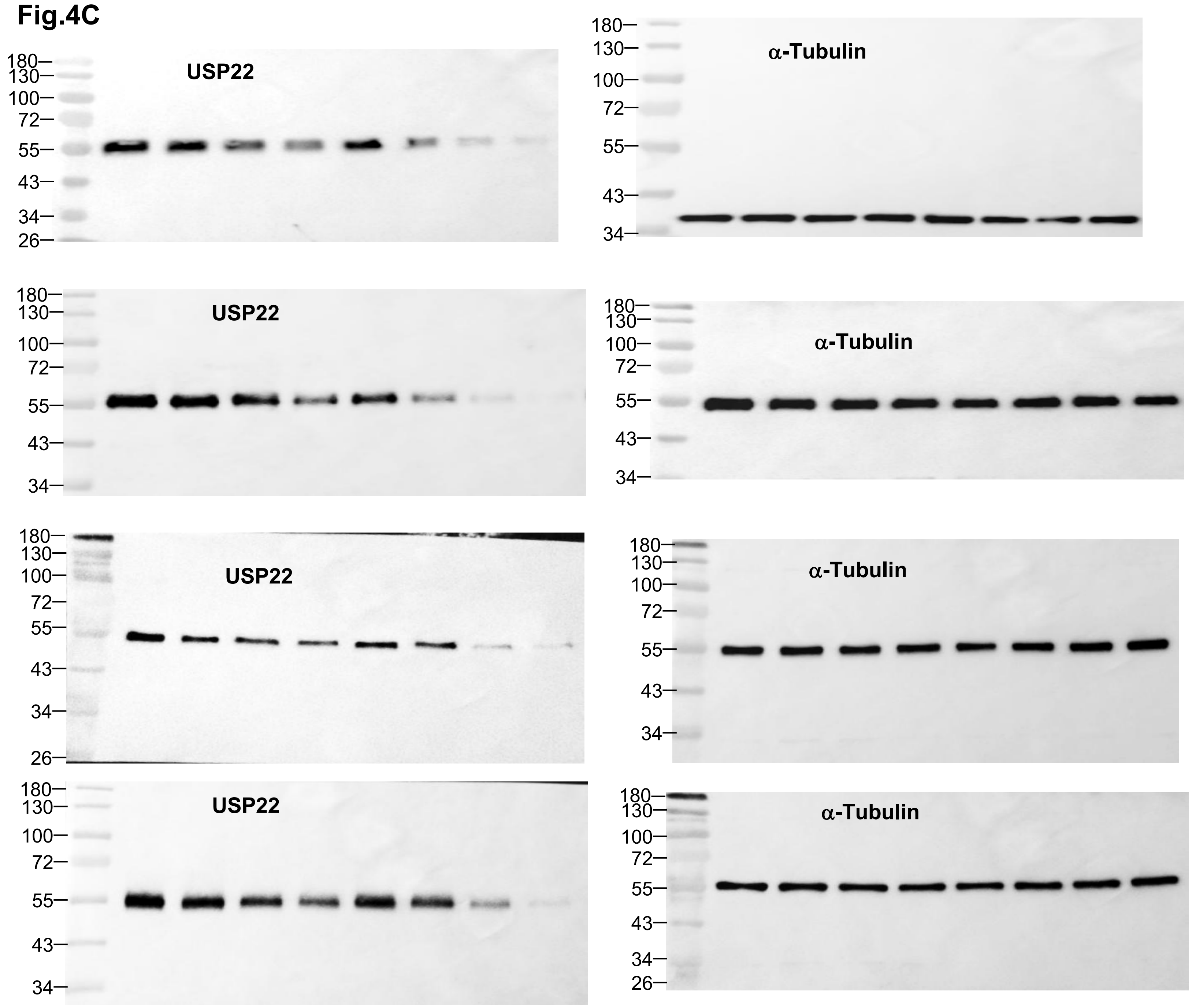

Fig.4D

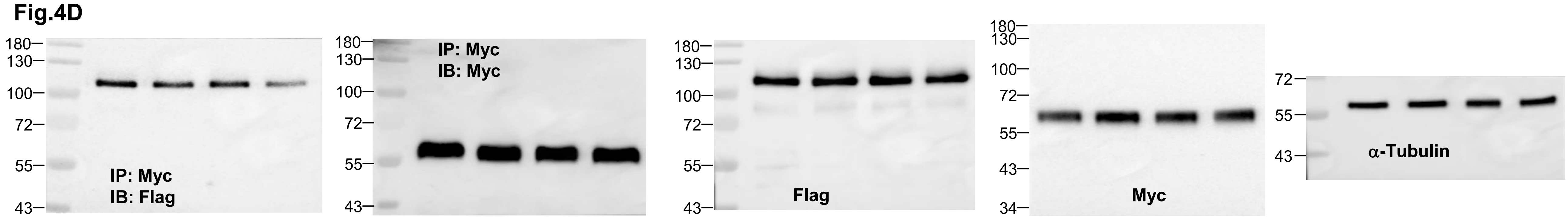

Fig.4E

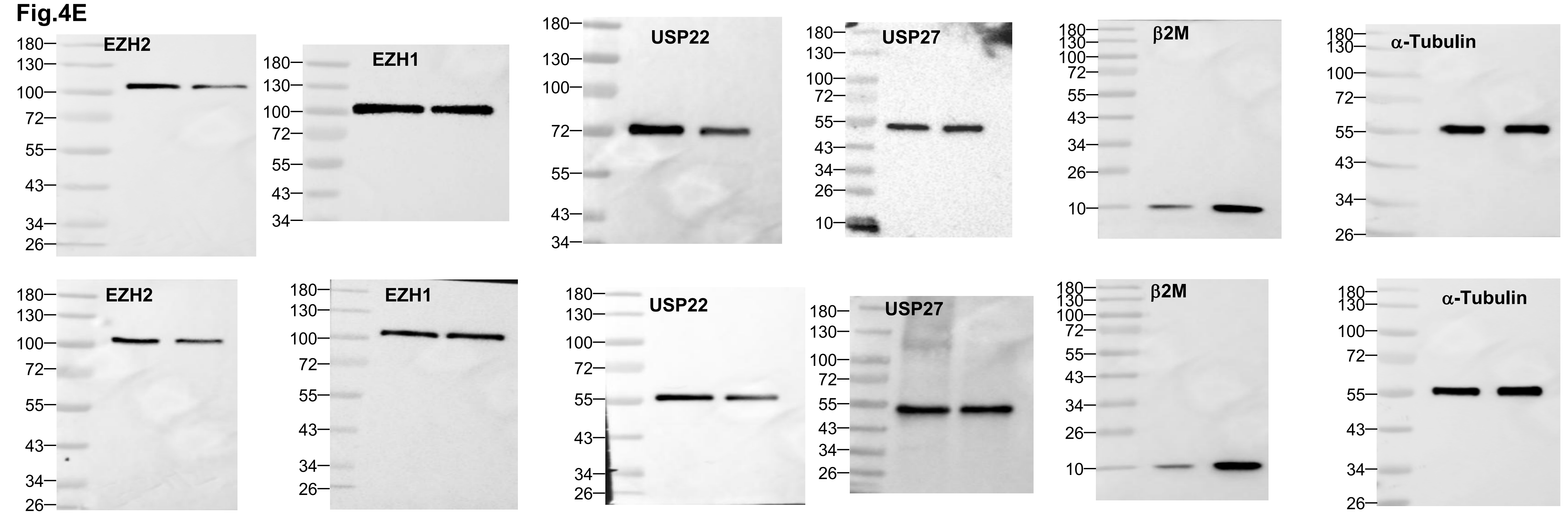

Fig.4F

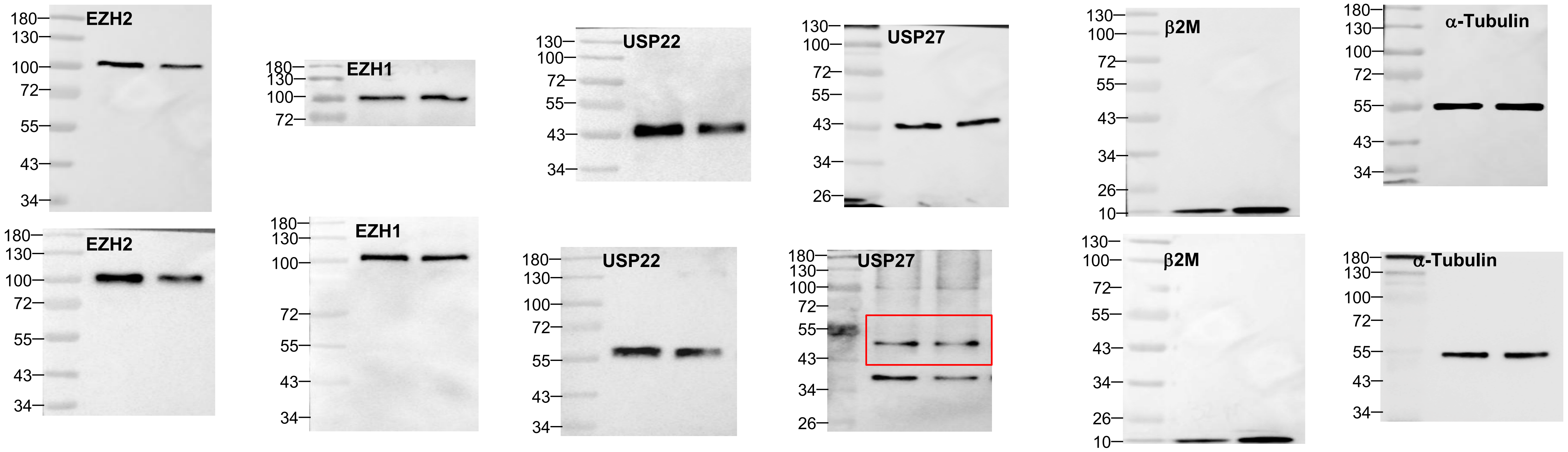

Fig.4I

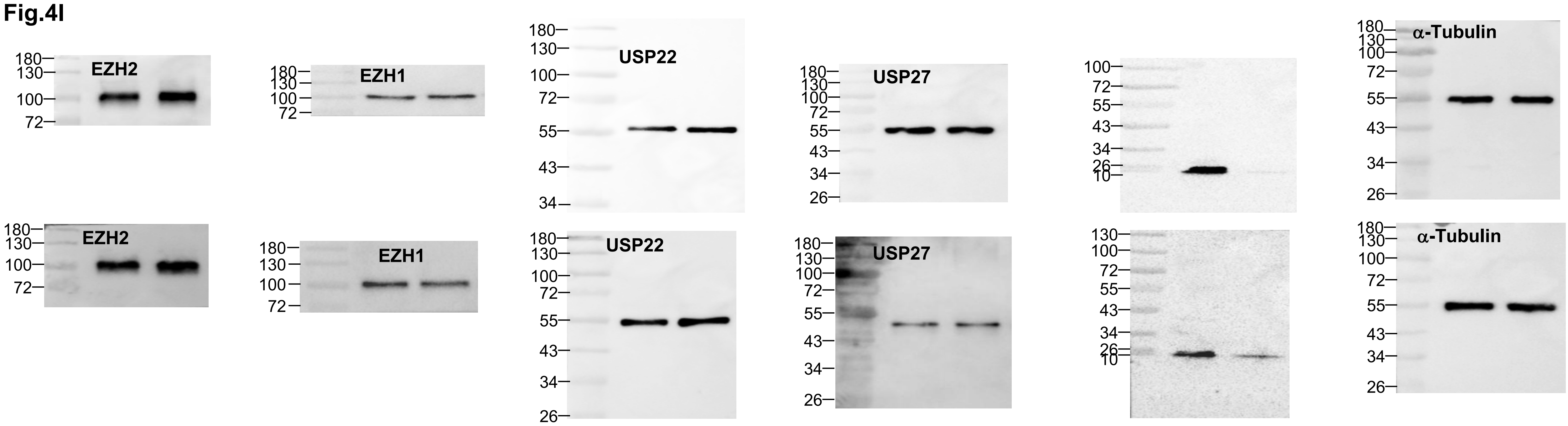

Fig. S1A

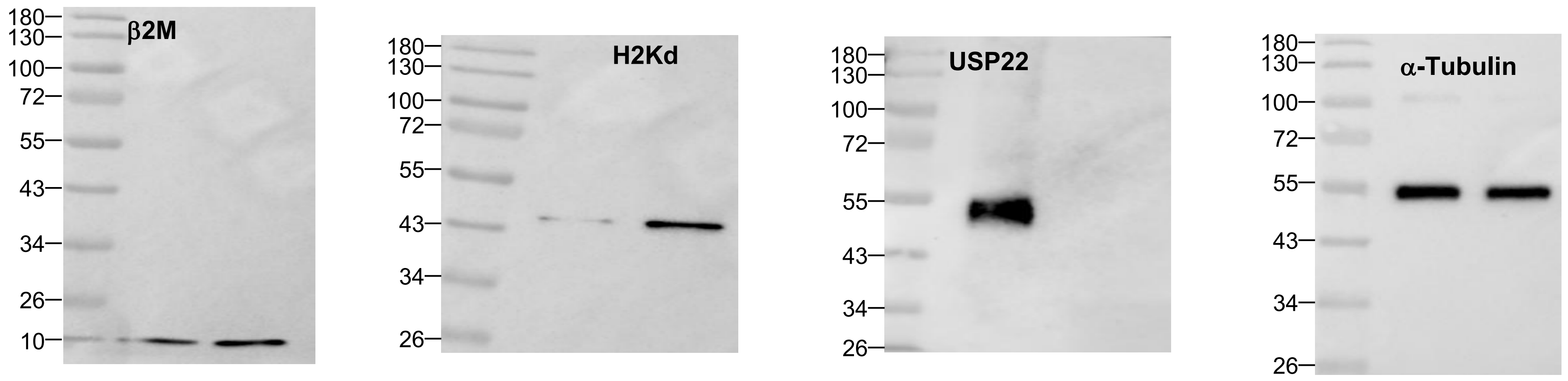

Fig. S1D

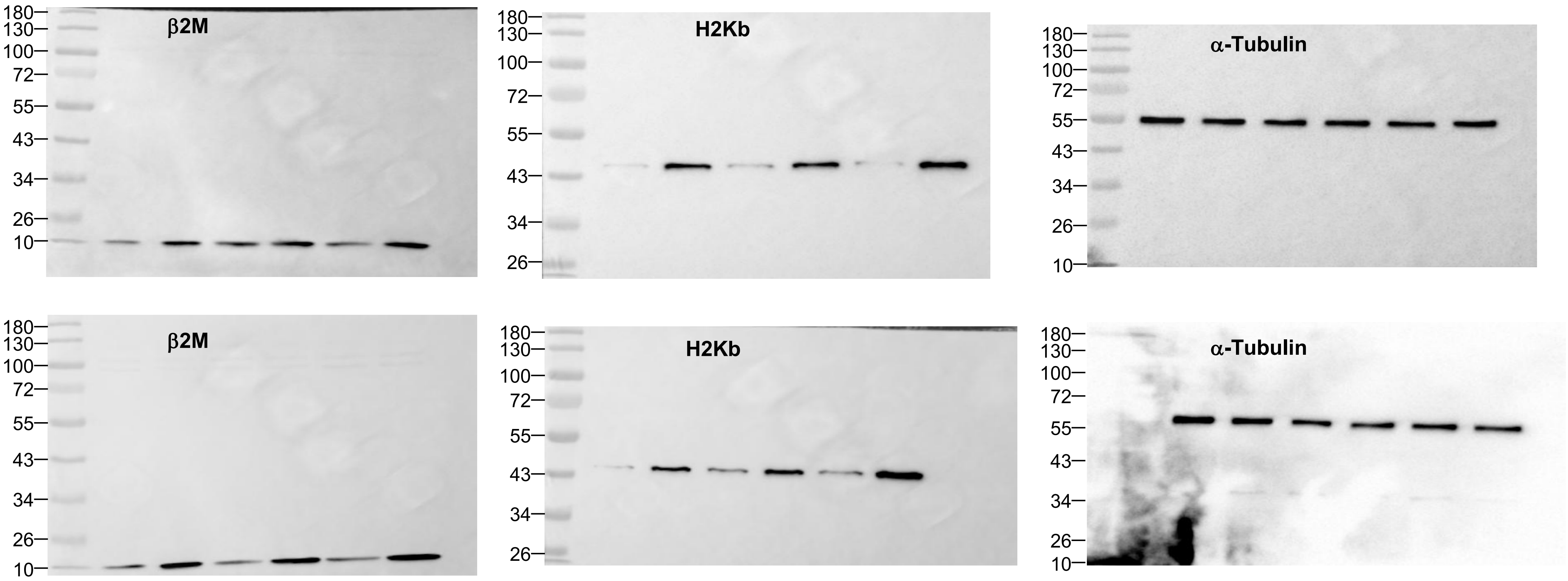

Fig. S1G

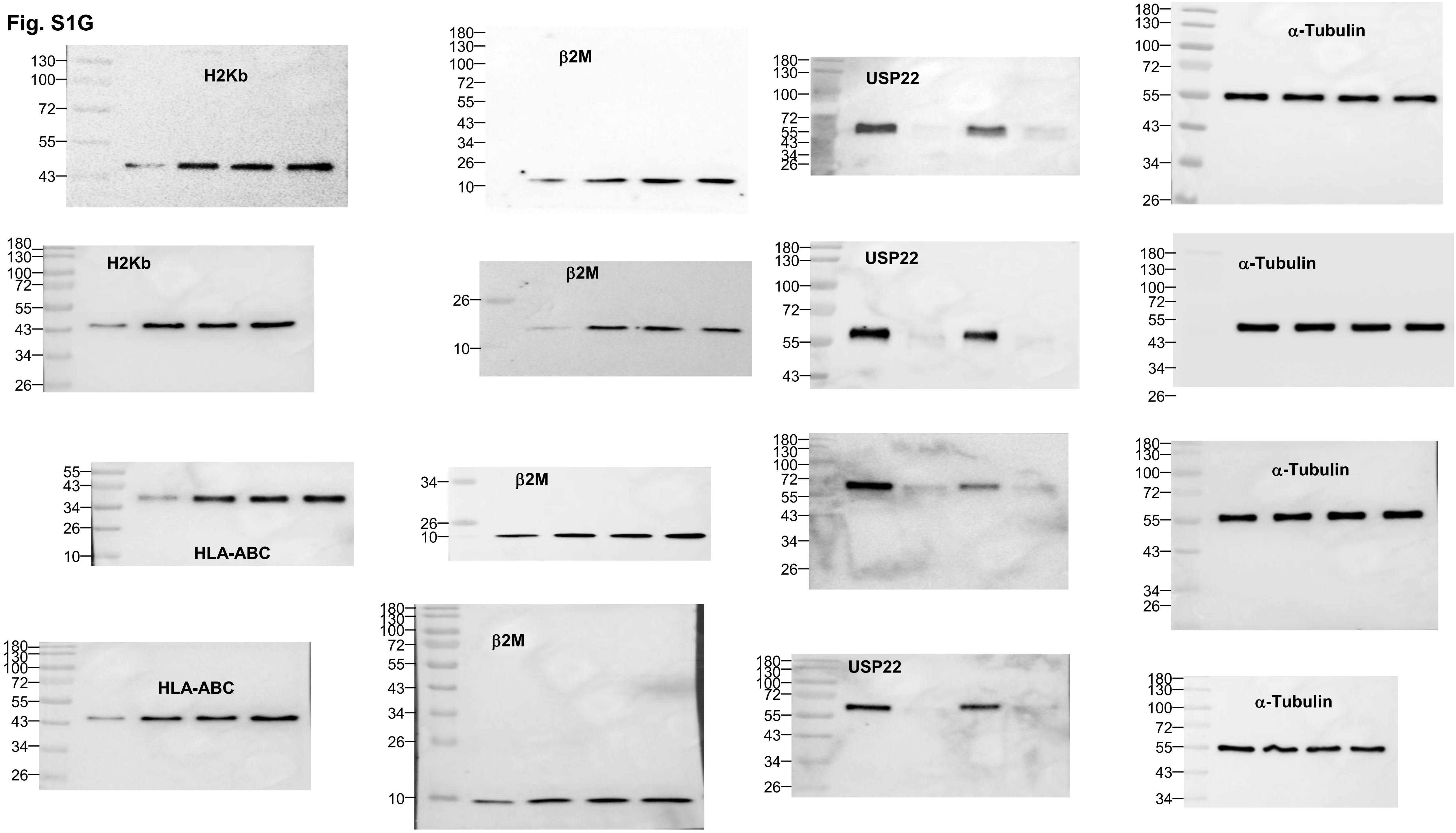

Fig.S7A

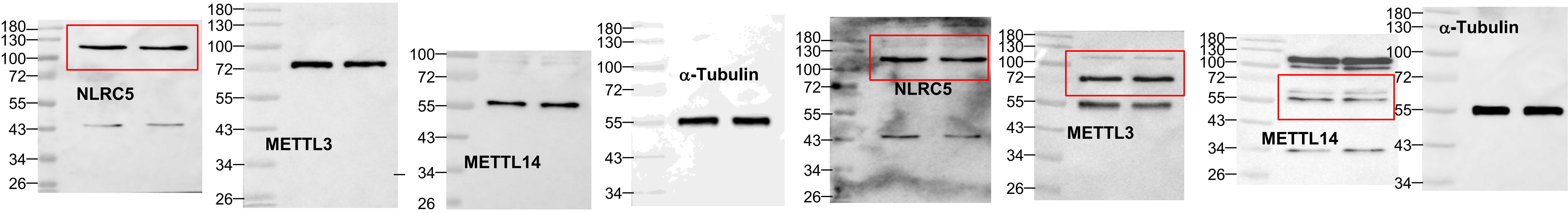

Fig. S7D

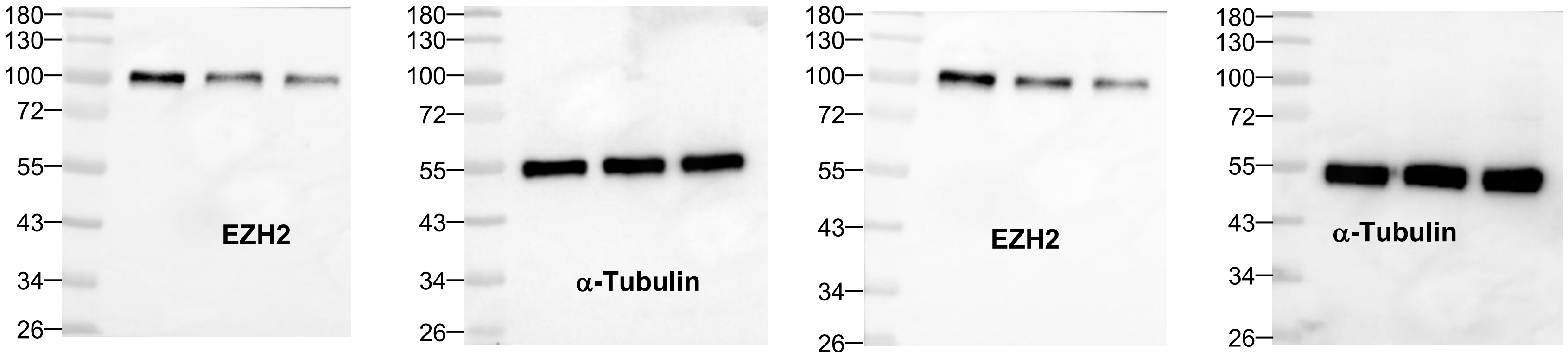

Fig.S7E

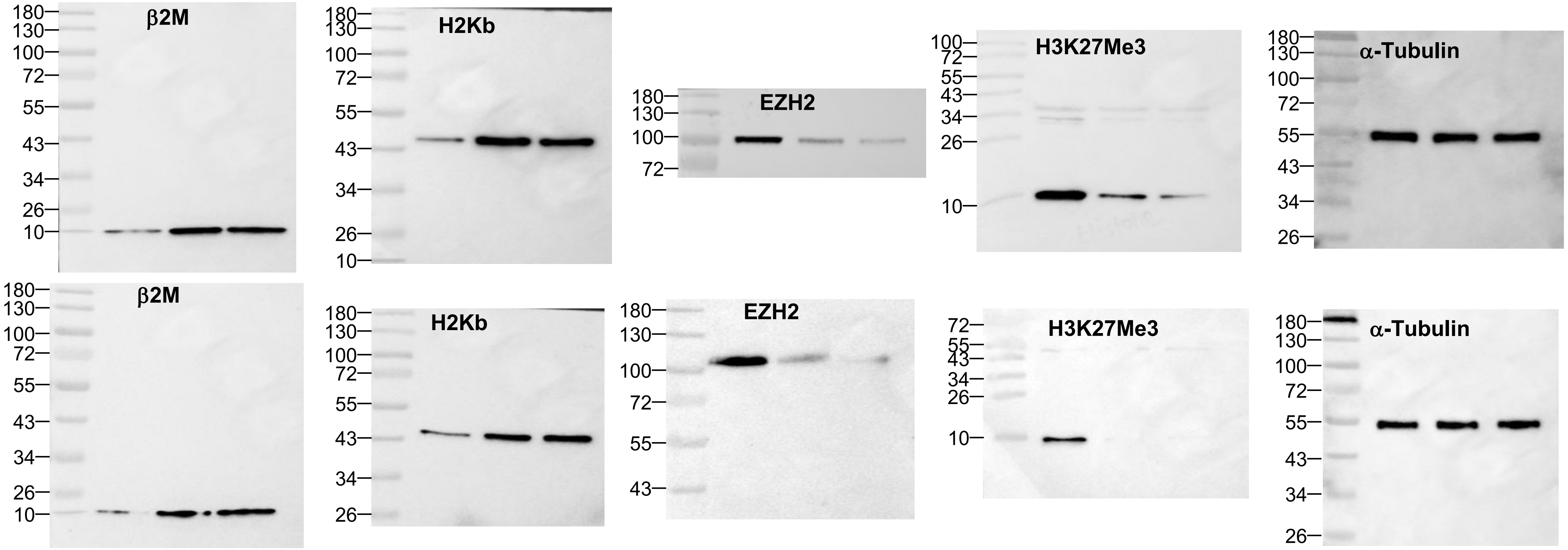

Fig.S8B

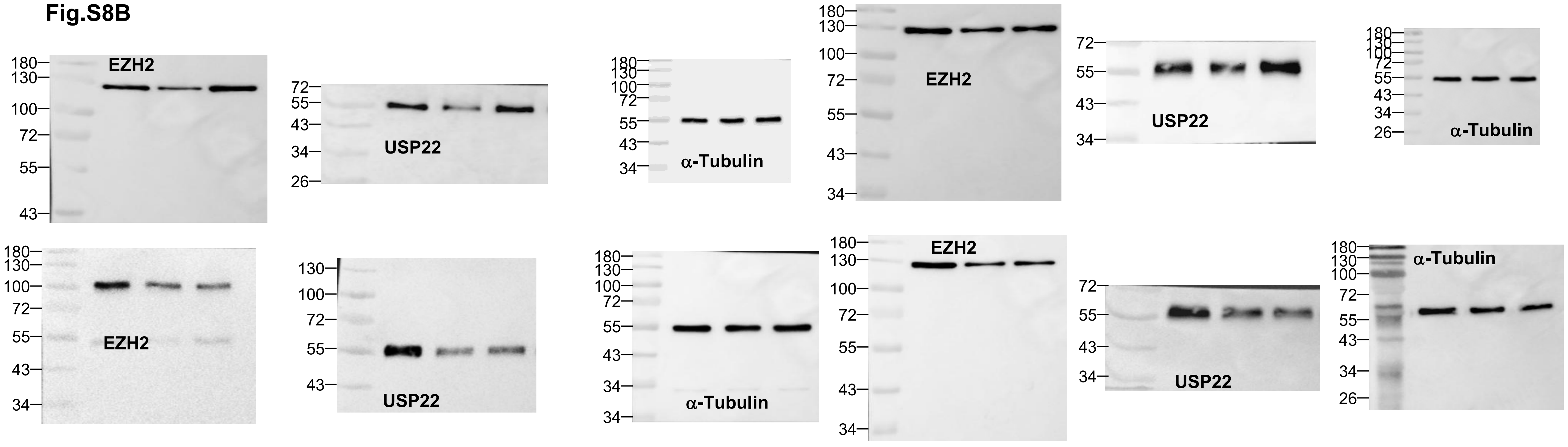

Fig.S8C

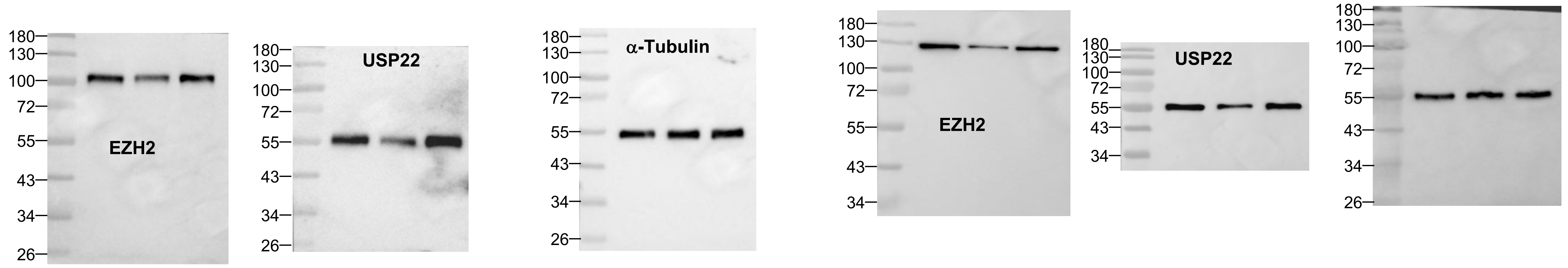

Fig.S8E

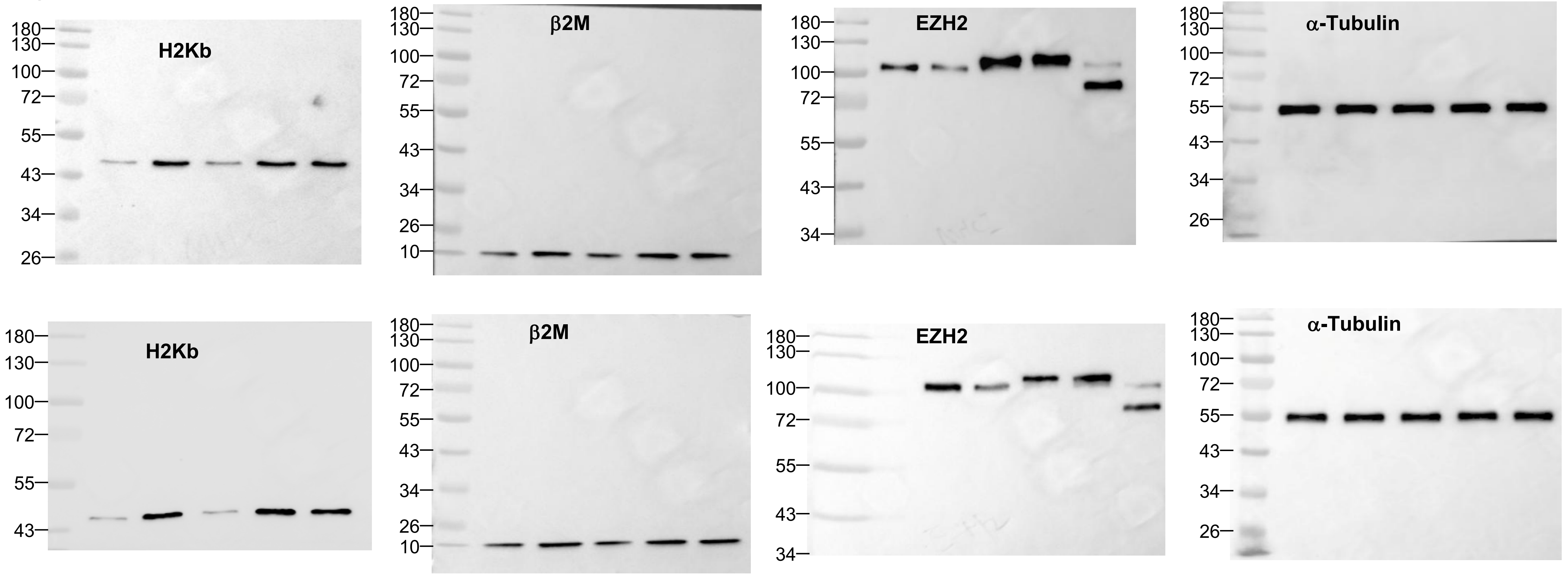

Fig. S14C

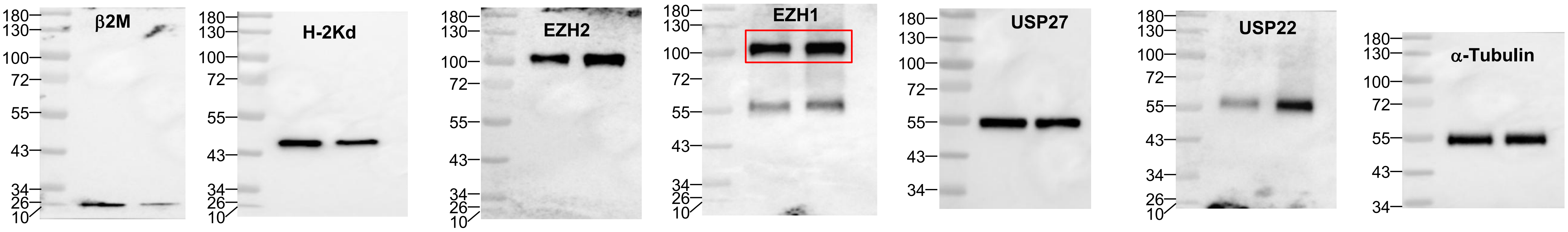

Supplement: Unedited blot and gel images [file jci-136-193162-s316.pdf]
